# An enhanced national-scale urban tree canopy cover dataset for the United States

**DOI:** 10.1038/s41597-025-04816-0

**Published:** 2025-03-24

**Authors:** Lucila M. Corro, Kenneth J. Bagstad, Mehdi P. Heris, Peter C. Ibsen, Karen G. Schleeweis, Jay E. Diffendorfer, Austin Troy, Kevin Megown, Jarlath P. M. O’Neil-Dunne

**Affiliations:** 1 U.S. Geological Survey, Geosciences and Environmental Change Science Center, Denver, CO 80225 USA; 2 Hunter College, Urban Policy & Planning, New York, NY 10065 USA; 3 Forest Inventory and Analysis, U.S. Forest Service, Rocky Mountain Research Station, Riverdale, UT 84405 USA; 4 College of Architecture and Planning, University of Colorado Denver, University of Colorado Denver, Denver, CO 80202 USA; 5 Geospatial Technology and Applications Center, U.S. Forest Service, National Forest System, Salt Lake City, UT 84138 USA; 6 Spatial Analysis Laboratory, Rubenstein School of Environment & Natural Resources, University of Vermont, Burlington, VT 05405 USA

**Keywords:** Urban ecology, Forest ecology

## Abstract

Moderate-resolution (30-m) national map products have limited capacity to represent fine-scale, heterogeneous urban forms and processes, yet improvements from incorporating higher resolution predictor data remain rare. In this study, we applied random forest models to high-resolution land cover data for 71 U.S. urban areas, moderate-resolution National Land Cover Database (NLCD) Tree Canopy Cover (TCC), and additional explanatory climatic and structural data to develop an enhanced urban TCC dataset for U.S. urban areas. With a coefficient of determination (R^2^) of 0.747, our model estimated TCC within 3% for 62 urban areas and added 13.4% more city-level TCC on average, compared to the native NLCD TCC product. Cross validations indicated model stability suitable for building a national-scale TCC dataset (median R^2^ of 0.752, 0.675, and 0.743 for 1,000-fold cross validation, urban area leave-one-out cross validation, and cross validation by Census block group median year built, respectively). Additionally, our model code can be used to improve moderate-resolution TCC in other parts of the world where high-resolution land cover data have limited spatiotemporal availability.

## Background & Summary

Urban areas contain heterogenous mixes of land cover types, which are driven by and influence complex social-ecological processes^1–3^. The spatial dynamics of urban trees and forests directly affect multiple urban ecosystem services, such as cooling^4,5^, pollution mitigation^6^, access to green space^7^, water regulation^8^, carbon sequestration^7^, and noise mitigation^9^. Many analyses in urban systems rely on the use of remotely sensed products depicting tree canopy cover (TCC). However, the accuracy of these products to represent the size and shape of urban forest patches varies by geography^7,10^.

Increasing the resolution, consistency, accuracy, and availability of multi-city to national-scale land cover and TCC data is of great interest to researchers, policy makers, and urban planners. Multi-city to continental approaches quantifying urban phenomena for hundreds of cities simultaneously have become quite prominent in the past decade, for example, examining land cover drivers of urban heat in Europe^11^ and tree canopy–derived ecosystem services in U.S. cities^12,13^. Some multi-city studies have been completed using high-resolution land cover data to analyze distributions of ecosystem services^14^, but due to limited accessibility and standardization of the various high-resolution datasets, most recent work incorporating high-resolution data has focused on individual cities or regions. The greater precision provided by higher-resolution data is critical for multi-city to continental-scale studies as finer-grained data can often provide better estimates of land cover or tree canopy-derived urban ecosystem services compared to coarser satellite data^10^.

However, researchers generally face tradeoffs between moderate resolution (e.g., 10- to 30-m) TCC products suitable for long-term monitoring and high-resolution (e.g., 1- to 3-m or finer) products typically available for limited spatiotemporal extents or produced by commercial providers (i.e., not in the public domain). In this study, we aim to bridge this gap, using limited-availability, high-resolution TCC data, more widely available moderate-resolution TCC data, ancillary data, and machine learning methods to substantially improve the quality of moderate-resolution TCC data in the United States.

Key moderate-resolution TCC products include those derived from the European Space Agency’s Copernicus Sentinel-2 satellite and the U.S. Landsat program. The U.S. National Land Cover Database (NLCD), produced by the interagency Multi-Resolution Land Characteristics consortium, is a national Landsat-derived dataset for the United States, and includes both thematic land cover and continuous TCC and impervious cover^15,16^. These data encompass the conterminous United States, coastal Alaska, Hawaii, Puerto Rico, and the U.S. Virgin Islands. The thematic and percent impervious data are available in 2- to 3-year intervals from 2001–2021 and percent TCC data are available annually from 2011–2021, both at 30-m resolution. Substantial work has improved these products and their time series^17,18^. Meanwhile, Sentinel-2 data offer higher spatial resolution than Landsat (10 versus 30 m), and support a growing number of global thematic and continuous land cover products^19^. However, Sentinel-2 products have a shorter time series (dating to 2017) than Landsat-derived NLCD products (dating to 2001). This shorter historical time series is a major limitation in monitoring long-term land cover changes. Moreover, although the 10-m Sentinel-derived data show improvements over recent Landsat sensors for continuous canopy metrics^20^, it remains unclear whether 10 m is an adequate spatial resolution for understanding fine-grained spatial patterns of land and tree cover in urban areas^21^.

Overall, studies highlight how urban developed areas have an inherent spatial pattern whose fine scale cannot adequately be captured by 30-m-resolution spatial grain^22^. Because canopy cover does not scale linearly, 30-m scale observations measure different phenomena than 10-m or 1-m observations, leading to one potentially substantial source of uncharacterized error when urban TCC maps are generated from only 30-m imagery^10,23^. An optimal resolution for urban areas mapping has not been quantified, but the consensus is the higher the spatial resolution, the better to capture the inherent heterogeneity^24^. Forest fragmentation pattern metrics calculated using high-resolution input data demonstrate improved spatial precision of pattern indices in complex heterogeneous forests, including both urban and non-urban forests^17^.

By contrast, high-resolution (e.g., 1- to 3-m) urban land cover and TCC products are increasingly available for individual cities, produced by government agencies^25^, academic researchers^26,27^, and private companies^28,29^. Consequently, there is no central repository for such data, and some are not publicly available. Along with uneven access, these datasets are inconsistent in their production methodology, metadata, time of measurement, and the land cover classification schema. Important advances are also being made in public high-resolution ( ≤ 5 m) regional-scale land cover and TCC products, such as the National Aeronautics and Space Administration’s (NASA) Carbon Monitoring System^30^, the Chesapeake Bay Program^31^, and National Oceanic and Atmospheric Administration’s (NOAA) high-resolution Coastal Change Analysis Program^32^. Recent work has produced a TCC dataset for U.S. urban areas at 2-m spatial resolution, but this dataset is less accurate than previously mentioned products and intended to support Census block-scale, but not pixel-scale, analyses^33^. Other recent examples include a nationwide 0.5-m TCC dataset for 472 cities in Brazil^34^ and global-scale 1-m tree canopy height data^35^. Such novel approaches currently remain constrained by limited spatial and/or temporal coverage. This reduces their value for multi-city monitoring over time, a limitation that can be addressed by pairing high-resolution TCC data with limited spatiotemporal coverage with moderate-resolution data with broad spatiotemporal coverage.

**Fig. 1:**
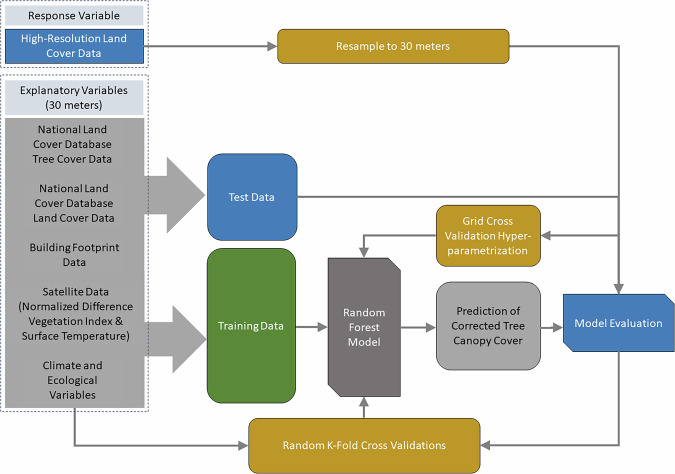
Conceptual diagram of analytical methods for corrected tree canopy cover modelling, incorporating the high-resolution land cover response variable, explanatory variables, random K-fold cross-validation for training data, grid search cross-validation for hyperparameter tuning, and continuous model evaluation.

In this paper, we develop an approach to substantially improve the quality of moderate-resolution TCC data through the use of high-resolution TCC data and other variables. The product described in this paper built on recent work using decision tree machine learning models to improve a moderate-resolution national TCC product, the NLCD TCC dataset, using NLCD land cover and TCC, high-resolution land cover data for 27 U.S. cities and counties, and moderate-resolution environmental correlate data describing urban form and climate^10^. Our work had three goals. First, we developed methods to further improve the performance of this previous U.S. urban TCC correction model using more urban areas that span wider gradients of climate and city sizes, using random forest models (Fig. 1). Second, we produced an enhanced moderate-resolution (i.e., 30-m), single-year (circa 2011) national urban TCC product that can be used as a stand-alone data product or incorporated into future NLCD TCC products to generate time series data across U.S. urban areas. Specifically, as NLCD products continue to evolve, these methods could be integrated into the future NLCD TCC production process within urban areas (similar work could investigate the possibility to improve TCC data for non-urban forests, using different predictor variables). Third, we provided model code to enable replication of the approach in the United States or other parts of the world where high-resolution land cover data can be combined with coarser resolution TCC data (e.g., Landsat or Sentinel-derived) and additional explanatory variables to produce an enhanced urban TCC product. Such data can support improved modelling of various urban socio-environmental phenomena that rely on an understanding of TCC and its changes over time.

## Methods

We applied random forest models to TCC data derived from high-resolution land cover products, along with additional explanatory variables to model urban TCC for 71 U.S. urban areas at 30-m resolution (Fig. 1). We then validated the model and applied its predictions to produce an enhanced urban TCC dataset for all urban areas in the conterminous United States. We describe the data used, how we defined and selected urban areas, the random forest model, and validation approaches below. Throughout this paper, we refer to comparisons between (1) native (i.e., unaltered) NLCD TCC data, (2) upscaled TCC data derived from high-resolution data as described below, and (3) enhanced TCC data, the output of our random forest model.

### Data sources

We used two different sources of high-resolution land cover data for 71 U.S. urban areas as a response variable in the random forest model. The high-resolution data used in this paper cover a range of geographies from small towns to the nation’s largest cities, as well as countywide data for several urban or suburban counties, and some data covering multi-county urban areas. For simplicity and consistency, we refer to these all as urban area datasets. High-resolution land cover data for 39 urban areas came from the U.S. Environmental Protection Agency (EPA) EnviroAtlas^25^ with a spatial resolution of 1 m. Data for the remaining 32 urban areas was sourced from the University of Vermont’s (UVM) Spatial Analysis Laboratory with a spatial resolution of 1 m or less^36^. Formal accuracy assessments for UVM New York City and Philadelphia tree canopy data had 98% and 97% user’s accuracy, respectively. While accuracy assessments were completed only for selected cities, because a similar procedure was used to produce data for all cities, including manual correction, tree cover data for other cities are expected to have similar levels of accuracy. Prior to running the random forest model, we resampled the categorical 1-m land cover data to represent 30-m continuous upscaled TCC data and reprojected all data into a common coordinate reference system (ESRI:102039). Any 1-m pixel of tree canopy, woody wetland, and/or orchard was considered “tree canopy” and aggregated to 30 m to estimate percent TCC. Given our focus on urban TCC, we clipped all high-resolution datasets to the U.S. Census Bureau’s 2020 urban area boundaries^37,38^. For five counties – Los Angeles and Sonoma, California, Anne Arundel and Montgomery, Maryland, and Jefferson, West Virginia - this meant that we split a single county-level dataset into multiple polygons aligned with Census urban areas. EPA data typically extend well beyond the boundaries of a single city, whereas UVM data typically cover smaller extents matching individual city boundaries. Although data for 27 cities and counties similarly analyzed by Heris *et al*.^10^ were clustered in the Northeast and Midwestern United States, our high-resolution data provided coverage for an additional 23 urban areas from other parts of the United States, spanning wider gradients of climate, city size, and city age. We reached a total of 71 urban areas as the sample size in this study because (1) there were some duplicate cities/urban areas provided by both EPA and UVM data and (2) some of the original county/multicounty high-resolution datasets were split into multiple urban areas when clipping them using Census urban area boundaries. These high-resolution data covered a total area of 143,273 km^2^, which represents about 52.3% of Census urban areas.

We used a total of 14 explanatory variables (Fig. 1) that act as biophysical and structural drivers of urban tree canopy in our machine learning model, with all datasets assembled for each urban area, aligned, and snapped to a common 30-m raster grid. To represent high temporal resolution (i.e., annual) changes in urban structure, we used (1) thematic land cover, and continuous (2) impervious surface, (3) tree canopy data, and (4) tree canopy standard error, all from NLCD for map year 2011 (2019 NLCD release)^39^. Other structural influences on tree canopy included building footprint data for (5) total building footprint coverage per cell, (6) number of buildings that intersect each cell, and (7) area of the average building per cell^40^. We sourced building data from Microsoft, which were developed using aerial imagery from varying time intervals, so are not associated with a specific year^40^. Additionally, we incorporated (8) Census block group-scale data on median year built of structures to incorporate the effect of neighborhood age on TCC. Collectively, these structural parameters represent how urban form can either increase (through plantings) or decrease (through mortality) TCC^41–43^. Six additional explanatory variables represent biophysical and structural drivers of urban tree canopy, including remotely sensed data for (9) surface temperature and (10) Normalized Difference Vegetation Index (NDVI), both derived from cloud-free summer Landsat 8 imagery for the years 2013–2015, mean urban area climate data from PRISM Gridded Climate Data^44^ including (11) average high temperature of the month of August (°C), (12) lowest temperature for the month of January (°C) and (13) annual precipitation (mm), and (14) EPA level II ecoregions for each urban area^45^. These data represent biophysical and climatic controls on tree growth and function.

### Urban areas definition

**Fig. 2:**
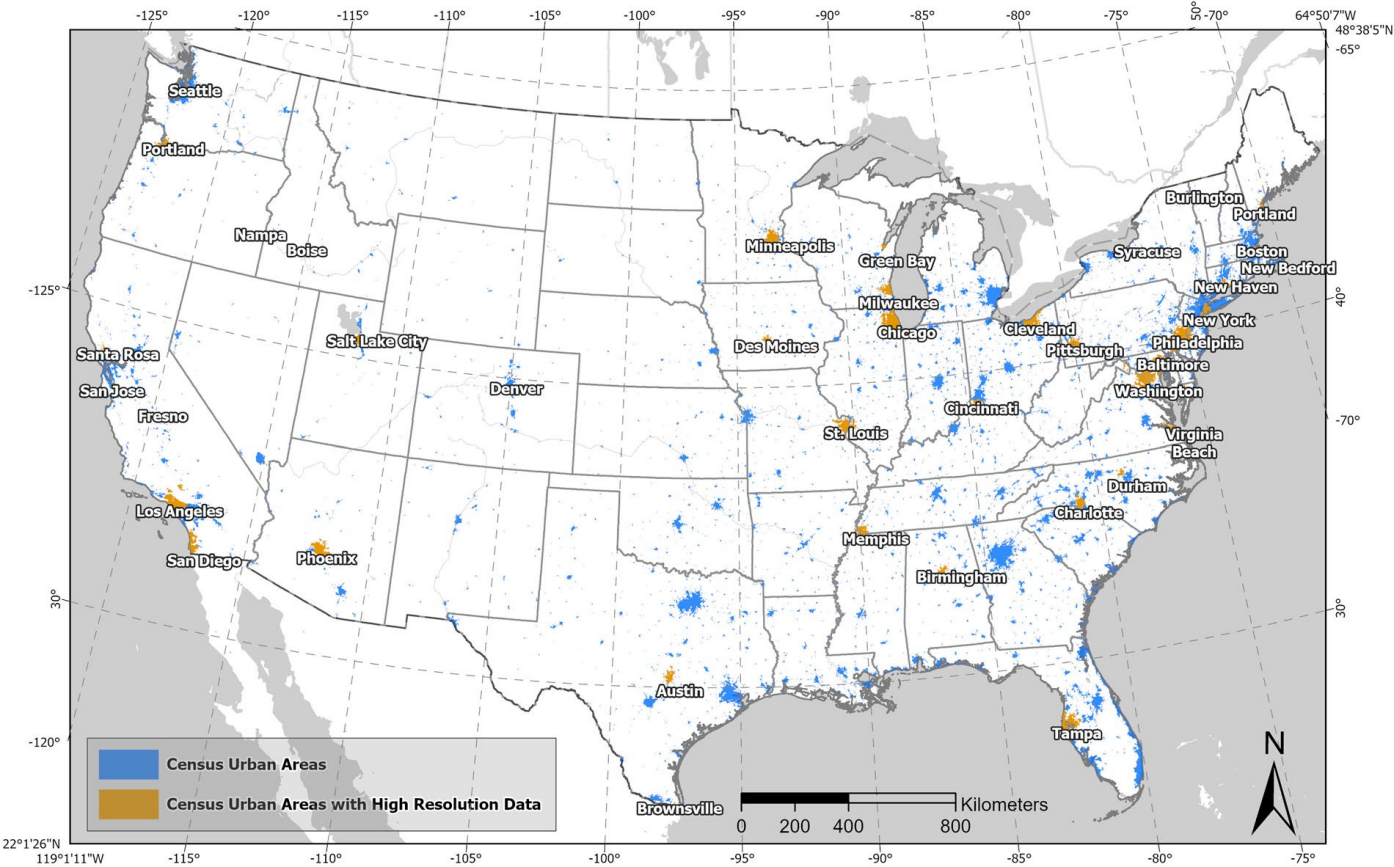
Distribution of urban areas across the contiguous United States, differentiated by standard Census Urban Areas (273,844 km^2^) and those with high-resolution data available (143,273 km^2^). Some small urban areas are not visible at the national scale, and due to the clustering of some urban areas, not all 71 urban areas are labelled on this map.

The final urban TCC dataset follows the boundaries of the Census 2020 Urban Areas Dataset, an area covering 273,844 km^2^, or about 3.6% of the conterminous United States (Fig. 2)^38^. The Census Urban Areas Dataset provides a nationally consistent definition of urban form, incorporating both population density and land use characteristics, enabling us to avoid potential discrepancies that might arise from using multiple, locale-specific definitions.

We used the updated Census urban areas dataset developed following the 2020 Census, which redefines urban areas, maintaining a focus on population density and developed land while incorporating several key changes^38^. The Bureau now defines urban areas as regions with a population of at least 2,500, a noteworthy reduction from the previous threshold of 50,000. Another important change is the introduction of a housing unit density threshold, replacing the previous impervious surface criterion for defining urbanized regions. The Bureau has also added a proximity criterion, which designates areas with at least 385 housing units per square mile near urban areas as part of an adjacent urban areas. These modifications offer a more nuanced perspective on urbanization, encompassing not only dense city centers but also less dense, yet substantially urbanized suburban and exurban regions.

### Machine learning algorithms and tuning

We developed random forest models and predictions using the Python ‘sklearn’ package version 1.0.2 and the ‘statsmodels’ package version 0.13.2^46,47^. To assess the model’s predictive performance, we allocated 70% of the data for training and 30% for testing. Our models built on existing random forest and decision tree models for urban TCC^10^; we used a common hyperparameter tuning workflow to improve random forest model predictions and include additional urban areas^48^. To optimize our random forest model, we used both Random Search and Grid Search cross-validation methods to create and test a series of models with varying hyperparameters to optimize model predictions. We then selected the optimal hyperparameters based on the results of the hyperparameter tuning to develop our best random forest model. We assessed models during tuning using the accuracy metrics Root Mean Squared Error and the proportion of variance (R^2^).

## Data Records

**Fig. 3:**
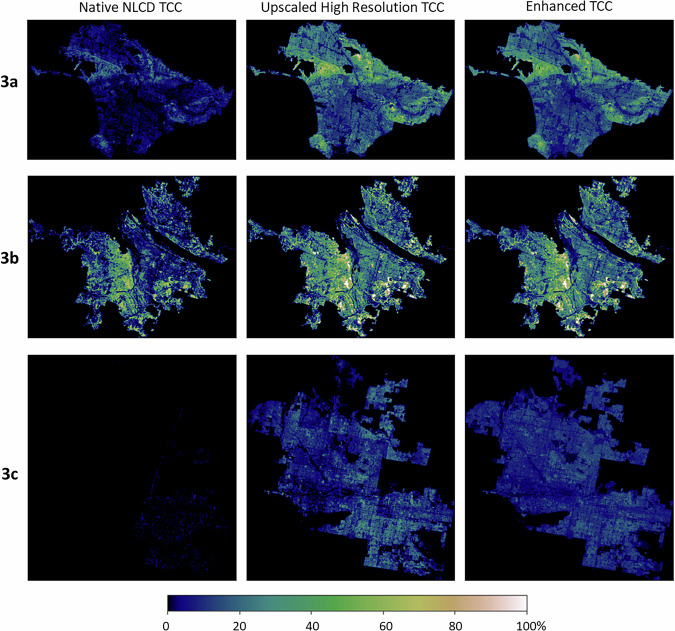
Tree canopy cover (TCC) distribution in (3a) Los Angeles, California, (3b) Portland, Oregon, and (3c) Phoenix, Arizona, showing native National Land Cover Database (NLCD) tree canopy cover (left), upscaled high-resolution tree canopy cover (center), and enhanced tree cover using the random forest model (right).

The final data product is an enhanced 30 m resolution raster dataset for urban TCC for all Census Urban Areas within the conterminous U.S., represented by values from 0–100, with a nominal 2011 date (i.e., built using NLCD map data year 2011, high-resolution land cover data built using imagery from 2004–2016, and ancillary data described above). The 2020 Census Urban Areas dataset, and our TCC data product which covers it, includes a total area of 273,844 km^2^. As an example, Fig. 3 shows the native 2011 NLCD TCC dataset, upscaled high-resolution TCC data, and our enhanced (predicted) NLCD TCC product for the urban areas of Los Angeles, CA, Portland, OR, and Phoenix, AZ. Similar figures for all 71 urban areas are included as supplementary information (Supplemental File 1). Our data are available as a U.S. Geological Survey data release at 10.5066/P13LECKC^49^. Included in the data release are a cloud optimized GeoTiff, all supplemental files, training data, python code, and a metadata XML^49^.

## Technical Validation

### Model performance

**Fig. 4:**
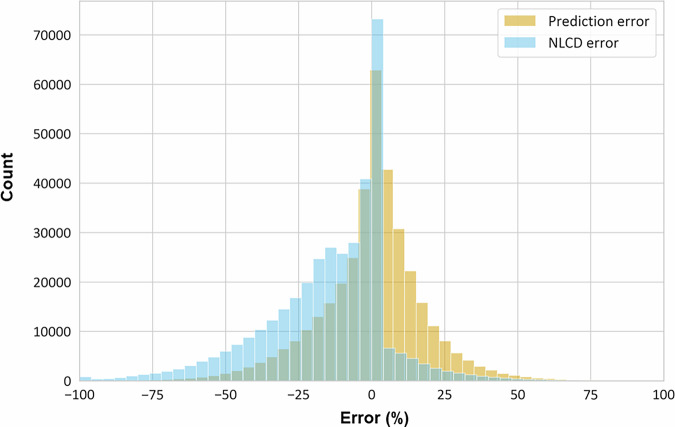
Summary of the distribution of error in National Land Cover Database (NLCD) tree canopy cover (TCC) and our Predicted TCC (deviation by % when native NLCD TCC are compared to upscaled high-resolution TCC data versus enhanced TCC).

**Fig. 5:**
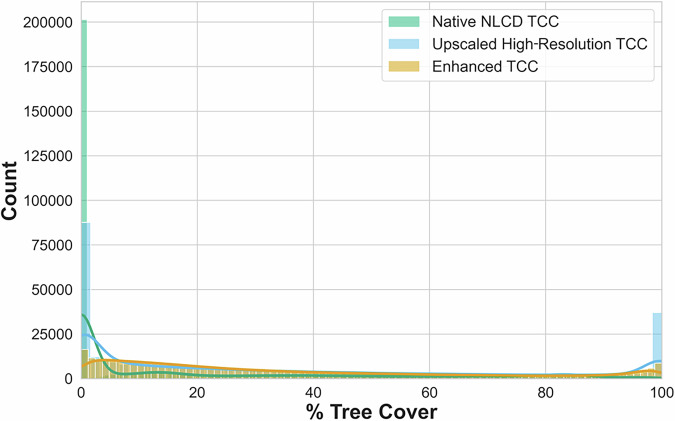
Comparative distributions of tree canopy cover (TCC) data from three data sources: (1) native National Land Cover Database (NLCD) tree canopy data (blue); (2) upscaled high-resolution tree canopy data (green); and (3) enhanced tree canopy data (red).

We evaluated our model’s performance using R^2^, Root Mean Squared Error (RMSE) and Mean Absolute Error (MAE). Our enhanced TCC model’s overall R^2^ value of 0.747 showed that it explained variation in TCC relatively well. Additionally, our model substantially normalized the distribution of error (the difference between upscaled TCC derived from high-resolution data and native NLCD TCC), Fig. 4). Our model consistently improved TCC estimates relative to the native NLCD TCC dataset, because NLCD’s 30 m resolution inherently misses finer-grained tree canopy that is detected by higher-resolution data, which we upscaled. Heris *et al*. (2022) previously evaluated how climate and urban structure influence the inherent underestimates in NLCD TCC^10^. Except at extremely low and high values, our modelled TCC values (smoothed orange line), were generally closer to the high-resolution values (green line) than the uncorrected NLCD TCC (blue line; Fig. 5).

At the level of individual urban areas, R^2^ values ranged from 0.292 (Phoenix-Mesa, AZ) to 0.813 (Portland, ME); 52 of 71 urban areas had R^2^ values of 0.6 or greater (Supplemental File 2 and in the U.S. Geological Survey data release^49^). The native NLCD TCC product underestimated TCC in all urban areas – by as little as 3.5–3.7% in Shannondale, West Virginia, and Durham, North Carolina, by 13.4% on average across the urban areas, and by as much as 29.4% in Forestville, California. Our TCC model added TCC to correct these underestimates (3.6–5.1% added TCC in Durham and Charlotte, North Carolina, 12.5% on average across the urban areas, and 20.7% and 24.7% for Tampa-St. Petersburg, Florida, and Forestville, California, respectively).

**Fig. 6:**
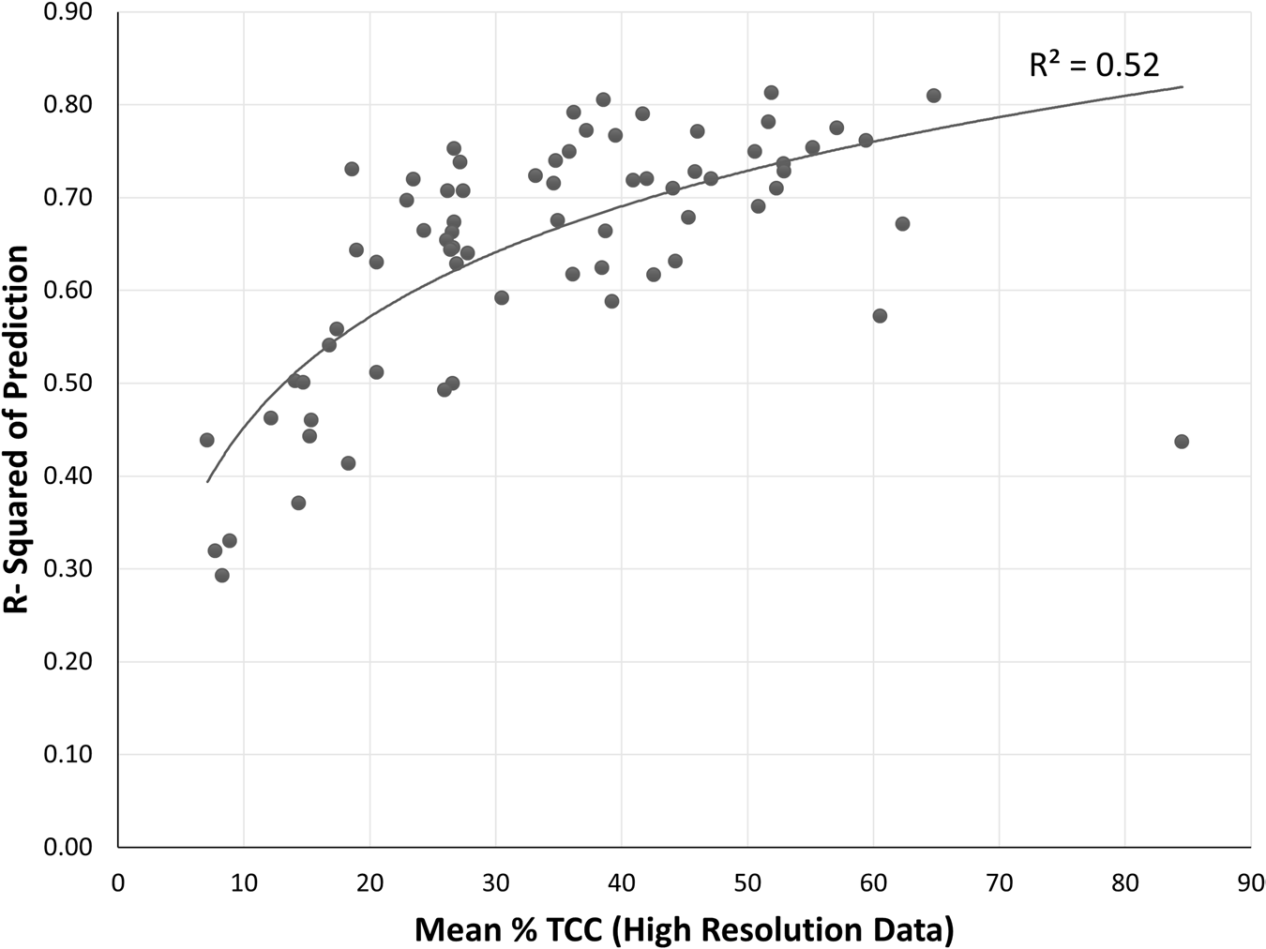
Scatter plot of coefficient of determination (R^2^) values for each urban area versus their respective mean high-resolution tree canopy cover (TCC), with a quadratic line of fit.

**Fig. 7:**
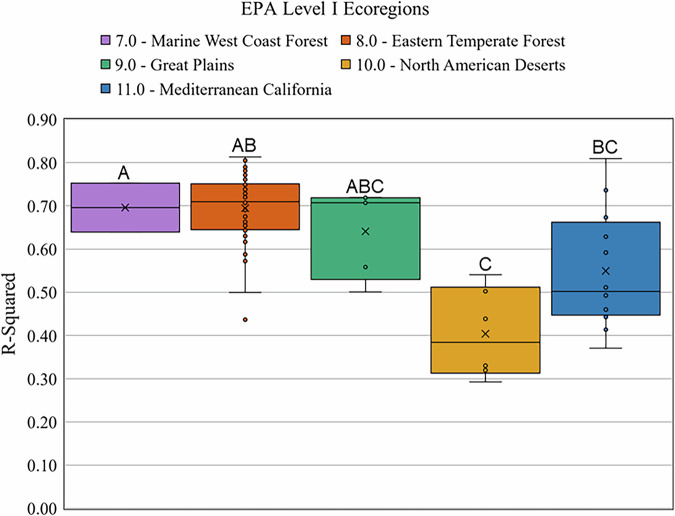
Boxplot depicting the distribution of coefficient of determination (R^2^) values across different U.S. Environmental Protection Agency (EPA) Level I Ecoregions. The boxes represent the interquartile range (IQR) of the R^2^ values, the horizontal line within each box indicates the median, and the ‘x’ marks represent the mean. Outliers are shown as individual points outside the whiskers, which extend to 1.5 times the IQR from the box. Letters correspond to statistically significant differences in mean R^2^ values between the Ecoregions using the Tukey’s Honestly Significant Difference post-hoc analysis.

Model performance was generally better in urban areas with higher TCC (coefficient of determination (R²) = 0.52, Fig. 6). Three small humid-region urban areas with extensive forested cover within or just outside the cities had TCC above 60% but R² values below 0.7 - Williamsburg, Virginia; Shady Side-Deale, Maryland; and Shannondale, West Virginia. In fitting a quadratic relationship to Fig. 6, we assumed that the relationship between TCC and R² for these three small cities is atypical and was not indicative of national-scale patterns. By contrast, urban area-level model R² was weaker in areas with the least TCC, typically found in the arid regions of the western United States. Using Analysis of Variance (ANOVA) with Tukey’s Honestly Significant Difference post-hoc analysis, we found statistically significant differences in R² values by EPA Level I ecoregions (p < 0.01, Fig. 7), with urban areas in more arid ecoregions tending to have lower R² values. However, urban area R² values had negligible relationships with climate variables, including mean annual precipitation, January minimum temperature, and August maximum temperature, with R² values for linear regression models of 0.040, 0.004, and 0.014, respectively.

Prediction error (difference between upscaled and native NLCD TCC versus enhanced and native NLCD TCC) was substantially reduced – to 0.9% on average – compared to the existing error in the native NLCD TCC dataset – 13.4% on average – although it was overestimated by 2.1–3.2% in four urban areas and underestimated by 2.0–7.1% in 15 urban areas. When evaluating prediction errors using Root Mean Square Error (RMSE) and Mean Absolute Error (MAE), our enhanced TCC model consistently outperformed the native NLCD TCC product. Urban area RMSE values for the enhanced TCC ranged from 8.3% to 24.7%, substantially lower than the native NLCD TCC’s RMSE range of 13.0% to 37.0%. Similarly, MAE values for the enhanced TCC varied between 5.2% and 17.6%, compared to the native NLCD TCC’s MAE range of 7.1% to 29.9% (Supplemental File 2 and data release^49^). On average across all urban areas, the enhanced TCC reduced RMSE by 32% and MAE by 31%, indicating improved accuracy and precision.

In summary, while our model fits were generally good for most urban areas, there was a more consistent pattern of underestimation than overestimation in the predictions, as indicated by the negative prediction error for many urban areas. While the enhanced TCC predictions underestimate tree canopy, we have more effectively addressed the underestimations inherent in the native NLCD TCC data, as indicated by the substantial reductions in RMSE and MAE.

Four temporally dynamic explanatory variables derived from NLCD and two from Landsat imagery (i.e., NDVI and surface temperature) were the most influential predictors of TCC (Table 1). These six explanatory variables had a collective importance of 0.85, with the largest contributions from NLCD TCC (0.38) and NDVI (0.16). Other explanatory variables had importance values of 0.04 (Census block group year built, which will change slowly over time in neighborhoods experiencing changes in their building stock), 0.06 (collective value for three long-term climate average variables plus static EPA ecoregions) and 0.05 (collective value for three building datasets for which we lack temporally dynamic data). When rerunning the model without the building variables, the R^2^ value decreased slightly from 0.747 to 0.728, indicating a minimal reduction in the model’s explanatory power. Variable importances for the model without building variables remained consistent relative to the model with the building variables (e.g., NLCD TCC: 0.40, NDVI: 0.18, surface temperature: 0.10).

**Table 1:** Variable importance for each explanatory variable in the model.

Variable	Importance with Building Data	Importance without Building Data
NLCD^a^ Tree Canopy Cover (TCC)	0.38	0.40
Normalized Difference Vegetation Index	0.16	0.18
NLCD TCC Standard Error	0.09	0.09
NLCD Impervious	0.08	0.08
Surface Temperature	0.08	0.10
NLCD Land Cover	0.06	0.06
Median Year Built	0.04	0.04
Total Building Footprint Coverage	0.02	—
Average Area of Buildings Intersecting Each Cell	0.02	—
Average January Low Temperature	0.02	0.02
Average Annual Precipitation	0.02	0.02
Number of Unique Buildings Intersecting Each Cell	0.01	—
EPA^b^ Level II Ecoregion	0.01	0.01
Average August High Temperature	0.01	0.01

^a^NLCD: National Land Cover Database.

^b^EPA: Environmental Protection Agency.

The low importance of static building datasets and minimal impacts of their exclusion implies that our model predominantly relies on variables that can be readily updated for different years (e.g., NLCD, NDVI, surface temperature, median year built) and for which the use of static data is appropriate (multi-decade climate averages and ecoregions). This indicates that our model can be appropriately used to produce time series of enhanced TCC data by using updated versions of its most influential variables. Further, while temporally dynamic building data are to our knowledge not yet available in the United States, their recent release in the Global South portends the likely greater availability and accuracy of dynamic building data in the future^50^.

### Cross validation

We used randomized K-fold cross-validation using the Python ‘sklearn’ package version 1.0.2^47^ to measure model performance and validate the accuracy of the predicted TCC data. This process meant that the division of the data into K folds was performed randomly for each repetition, mitigating potential bias associated with non-random division and enhancing the robustness of model validation^51^. We assessed the performance of the model in each of the K iterations by computing the adjusted R^2^ score for each iteration prediction. The average performance across all K iterations provided a more robust estimate of the model’s ability to predict TCC accurately compared to a single random train-test split. We did not perform spatial cross validation because recent work indicates it lacks a robust theoretical basis and standard cross validation approaches often have less bias than spatial cross validation^52^.

In addition to a random K-fold cross validation, we performed a similar cross validation using individual urban areas (*k = *71). In each fold, a single urban area was left out as the validation set, and the model was trained on the remaining 70 urban areas. This procedure was iteratively performed 71 times, ensuring that each urban area was used as a validation set once. This method ensures that during training, the model is never exposed to data from the urban area being used for validation, providing a rigorous evaluation of its generalization capability across different urban regions.

Finally, to address the possibility that older neighborhoods may contain larger and more mature trees^41^, i.e., greater TCC, we performed a cross validation on unique Census block group-scale median year built (1939 to 2016; *k = *76). For each fold, one unique median year-built subset is held out for validation, while the remaining 75 subsets are used for training. This process is repeated 76 times, with each median year-built subset serving as the validation set once.

**Fig. 8:**
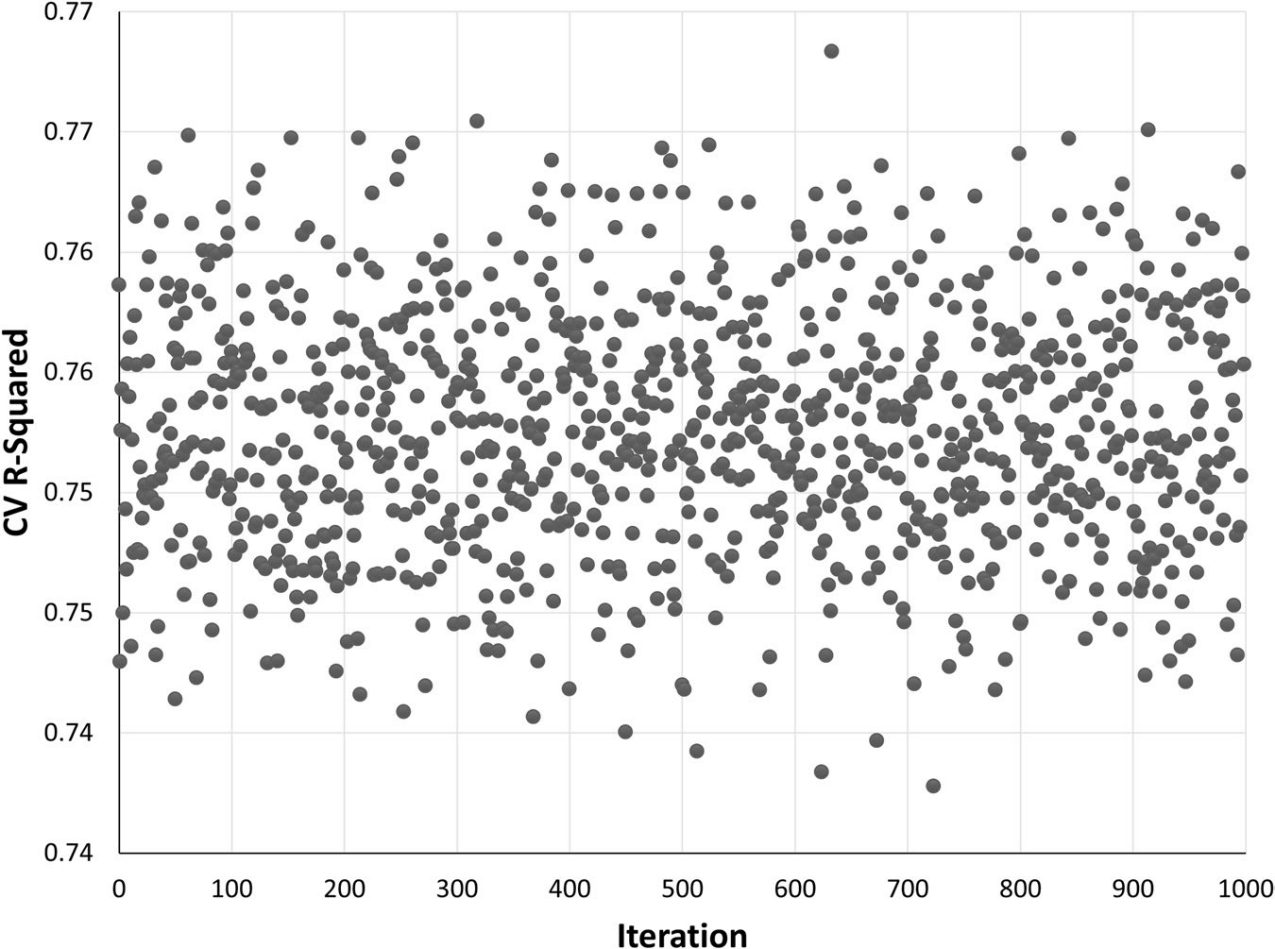
Scatter plot representing the results from a k = 1,000-fold cross validation (CV). Each point indicates the coefficient of determination (R^2^) value obtained from a single fold of the cross validation. The y-axis shows variation in model performance (R^2^ values) across the 1,000 iterations.

The 1,000-fold random cross-validation results indicated a high degree of stability in predicting outcomes from diverse data splits. Across the 1,000 iterations, R^2^ values ranged from 0.738 to 0.768, with a mean R^2^ of 0.753 (Fig. 8). Such consistency indicated that the model exhibits strong generalization capabilities when applied to unseen data, making it reliable as a basis for producing a national-scale data product.

**Fig. 9:**
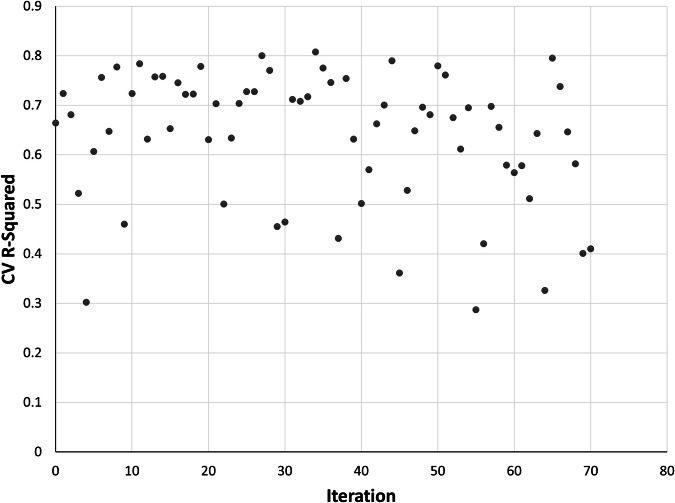
Leave-one-out cross-validation (CV) results of the 71-urban area tree canopy correction model, where each partition corresponds to a unique urban area. Each point indicates the coefficient of determination (R^2^) value.

R^2^ scores from the urban area leave-one-out cross-validation ranged from 0.287 to 0.808 when Charles Town-Ranson, West Virginia (Jefferson County urban area) and New York, New York (city) were left out of training, respectively (Fig. 9). The median value indicated that for a typical urban area, roughly 67.5% of the variation in the outcome was explained by the model. This exercise identified some cities that were relatively influential in the overall model, i.e., having city-specific models that performed well, but when holding out that city’s data and rerunning the model, had a reduced R^2^ value (primarily small cities in the eastern United States and northern California, but also Baltimore, Maryland; Durham North Carolina; Pittsburgh, Pennsylvania; and Seattle, Washington). Other cities’ R^2^ values increased during the leave-one-out cross validation, relative to the city-specific results. By leaving out data for these cities, model performance improved, which was the case in some arid cities with low initial R^2^ values (e.g., some cities in the intermountain west and southern California) but also in a few larger cities with higher initial R^2^ values (e.g., New York City, New York; Milwaukee, Wisconsin; Minneapolis-St. Paul, Minnesota-Wisconsin).

**Fig. 10:**
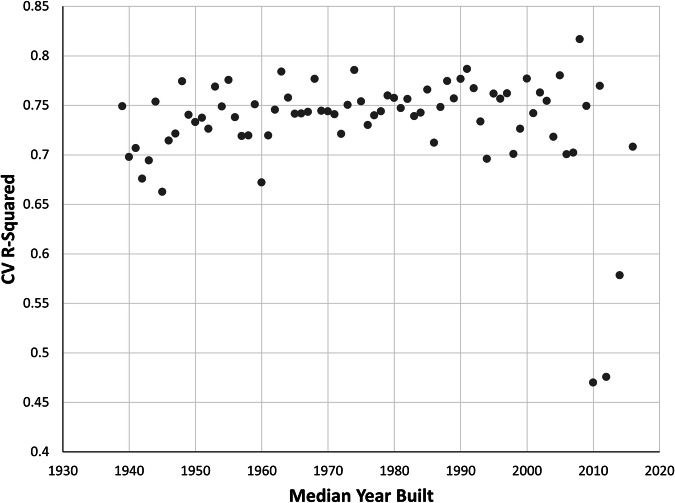
Cross-validation (CV) results across 76 iterations for different values of the median year built data by U.S. Census block group. Each point indicates the coefficient of determination (R^2^) value.

**Fig. 11:**
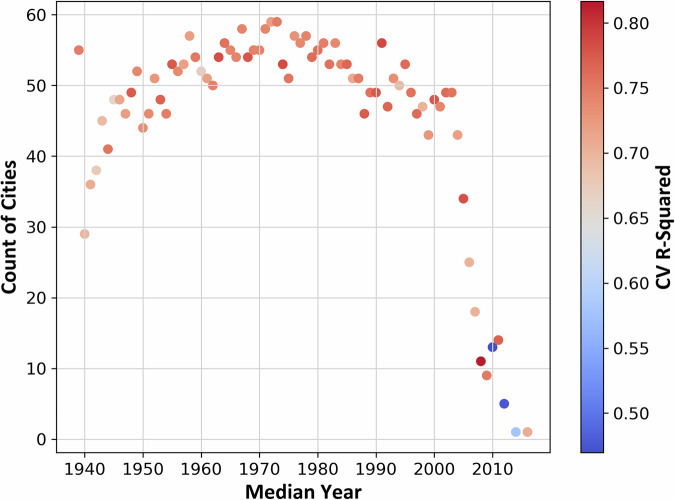
Scatter plot showing the number of cities with Census block groups having median year built each year, color-coded by cross-validation coefficient of determination (R^2^) values.

**Fig. 12:**
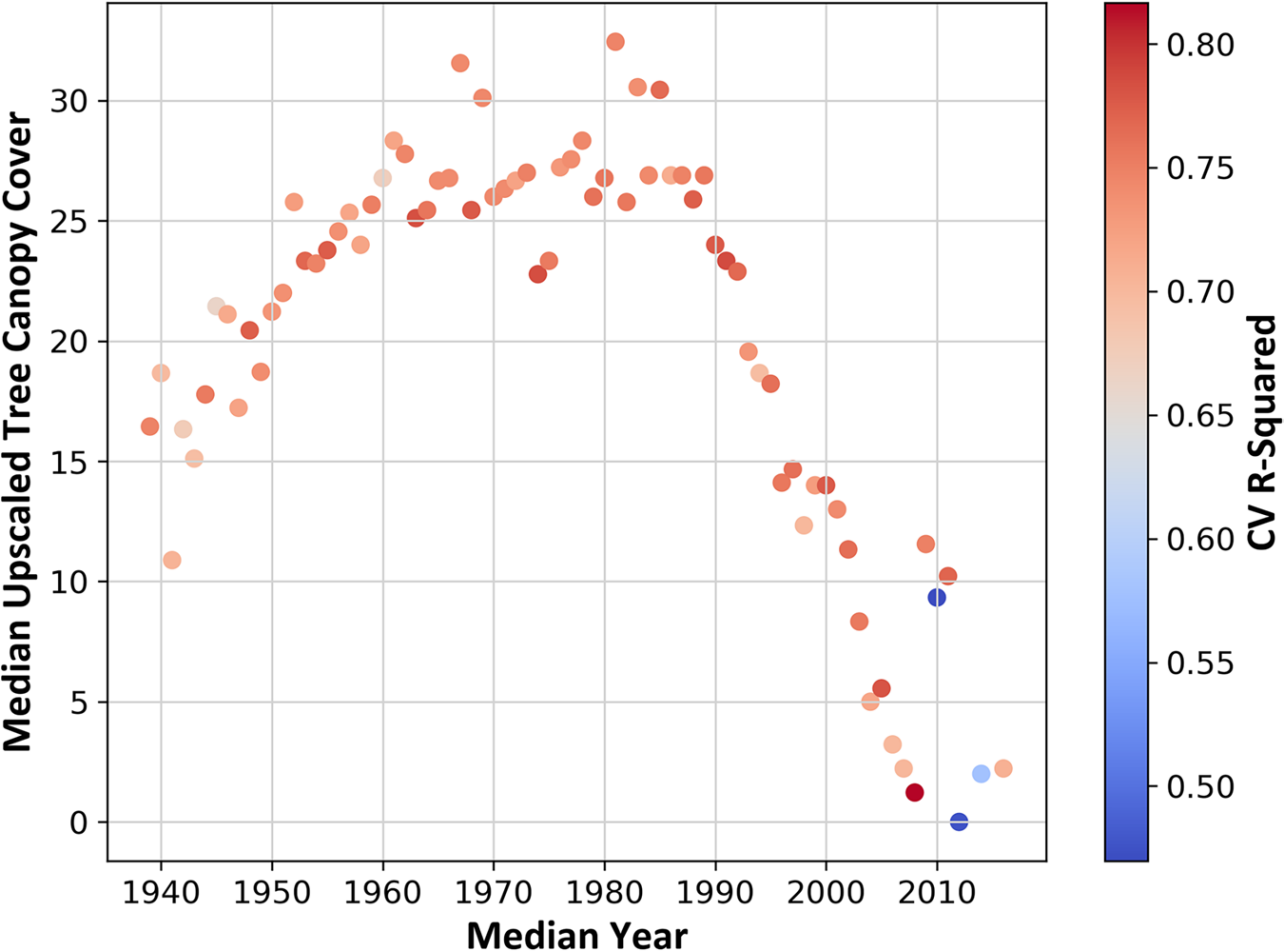
Scatter plot showing the median tree canopy cover (TCC) for each median year built, color-coded by coefficient of determination (R^2^) values obtained from cross validation.

The Census block group median year-built leave-one-out cross-validation produced relatively consistent R^2^ values (mean = 0.73), indicating that the model was able to consistently predict TCC with reasonable accuracy across various median Census block group year built, even when each specific year was excluded from the training set. Recent years had both some of the lowest values (2010, the lowest, at 0.470, as well as 2012 and 2014); 2008 had the highest R^2^ of 0.817 (Fig. 10). From the mid-2000s onward, the number of cities with median year-built Census block groups declined rapidly (Fig. 11), most likely in response to the instability in the housing market brought on by the Great Recession. With fewer cities represented in more recent years, median TCC was highly variable by median year built (Fig. 12).

In many cases, the few Census block groups within these more recent years are characterized by densely built areas with very little to no TCC. Our random forest model tended to underperform when predicting areas with minimal tree cover because of its difficulty in accurately predicting 0% TCC. The lack of trees within a small number of highly built Census block groups likely contributed to poorer model performance in 2012 and 2014, as random forests require a full range of input values to make accurate predictions, and these areas lack the variability in TCC that the model needed to learn effectively. Additionally, the 2011 map year NLCD TCC product is built using multiyear imagery for 2007–2011, with most data being from 2010. This may introduce some localized error in our model estimates for Census block groups with median year built around these years, for example, if 2007 imagery indicated TCC levels that changed substantially due to subsequent construction.

### Spatial autocorrelation

Positive spatial autocorrelation refers to the phenomenon where values of a variable observed at points near each other are, on average, more similar than those for points located more distantly from each other. In the context of large-scale geographic regression models, spatial autocorrelation can lead to biased parameter estimates, invalid standard errors, and ultimately Type 1 errors. This is because the number of reported samples will necessarily be higher than the number of spatially independent samples (independence is a requirement for Ordinary Least Squares regression), leading to inflation of test statistics in the absence of adjustment^53^. However, the degree of spatial autocorrelation in urban tree canopy can vary widely from one urban area to another due to factors including urban planning and zoning, demographic distribution, and environmental characteristics.

Spatial autocorrelation can be addressed by creating a spatial weights matrix that reflects spatial relationships specific to the area being analyzed, and subsequent application of spatial error models. Because our model aims to predict outcomes across a very diverse array of urban areas (Fig. 2), this would necessitate the development of a unique spatial weights matrix for each individual prediction area. Doing so would pose substantial challenges in terms of scalability and flexibility of the model, as each new prediction area would require the generation and validation of a new spatial weights matrix.

Moreover, the primary strength of random forest algorithms lies in their ability to handle large datasets with numerous variables and its robustness to overfitting, rather than in modelling spatial dependencies^31^. Being non-parametric, random forest models do not rely on assumptions about the distribution or structure of the data, including the independence of observations. This inherent flexibility allows them to better accommodate spatial autocorrelation by capturing complex spatial patterns and dependencies directly from the data, potentially mitigating the issues that biased parameter estimates and invalid standard errors commonly raise for parametric models. Finally, unlike when using traditional regression models, our focus is not on identifying which variables are most influential in predicting TCC or understanding the magnitude of their effects. Therefore, to maintain the model’s generalizability and applicability across a wide range of urban settings, and to ensure its scalability and feasibility, we did not include spatial error matrices and spatial error models from our random forest model^54–56^.

## Usage Notes

The purpose of the corrected TCC dataset described in this paper is to (1) enhance the accuracy and spatial distribution of mapped mean TCC within U.S.-wide urban areas and (2) do so in a way suitable for monitoring change over time, by using NLCD products as inputs. Our random forest model can be applied across more years than just the current 2011 dataset, and thus helps address key spatiotemporal coverage limitations of current high-resolution TCC data. Leveraging refined methodology and advanced algorithms, the methodology improves common inaccuracies found in urban areas for the NLCD TCC product, yielding a more precise depiction of the TCC in urban landscapes nationwide. By refining the spatial distribution of TCC, the dataset better reflects TCC variability and pattern, facilitating an improved understanding of the urban forest structure. Additionally, the augmented accuracy of the random forest-generated TCC provides critical support for various analyses quantifying both ecological processes and the ecological and socio-economic importance of urban forests, which contribute to sustainable urban planning and development. However, despite its strengths and spatial coverage, this dataset is not intended to accurately represent the precise locations of individual trees. Consequently, it should not be used to identify site-specific locations for planting individual trees. Its main strengths lie in the broad-level assessment and planning of urban forestry^57^, particularly in supporting time-series assessments, greatly contributing to the study and conservation of urban green spaces, and enhancing our ability to quantify and optimize the ecosystem services benefits provided by urban TCC.

The model code enables users to produce their own time series of urban TCC in regions within or outside the United States as new data become available. Users seeking to produce their own urban TCC datasets should be aware of the timing of all model inputs. The use of inputs that are temporally misaligned by more than a few years will increase the error of subsequent products. Greater error owing to temporal misalignment of inputs is most likely in urban areas or parts of urban areas that are being quickly developed or redeveloped, are affected by pest or disease outbreaks that may cause widespread tree mortality, or potentially in humid tropical to subtropical climates where longer growing seasons and greater water availability can lead to more rapid tree growth than in cooler and drier climates. We suggest that those reusing our code to produce time-series urban TCC datasets benchmark their data first, as we did, against the year(s) best matching underlying high-resolution TCC data, then proceed to compare modeled results to those of time-series moderate-resolution TCC products (i.e., the native NLCD TCC product, in our case).

## Supplementary information

Supplemental File 2

Supplemental File 1

## Data Availability

The code used to produce the data available is in the form of Jupyter notebooks. All code is publicly available at 10.5066/P13LECKC^49^.
