# Supplementary material for: An enhanced national-scale urban tree canopy cover dataset for the United States
Source: Sci Data. 2025 Mar 24;12:490. doi: 10.1038/s41597-025-04816-0 (PMC11933301; doi:10.1038/s41597-025-04816-0)
Supplement: Supplementary file 1 — Supplemental File 2 [file 41597_2025_4816_MOESM1_ESM.docx]

### Title

*An enhanced national-scale urban tree canopy cover dataset for the United States*

### Authors

Lucila M. Corro^1^, Kenneth J. Bagstad^1^, Mehdi P. Heris^2^, Peter C. Ibsen^1^, Karen G. Schleeweis^3^, Jay E. Diffendorfer^1^, Austin Troy^4^, Kevin Megown^5^, Jarlath P.M. O’Neil-Dunne^6^

**Affiliations**

1. U.S. Geological Survey, Geosciences and Environmental Change Science Center, Denver, CO 80225, USA

2. Hunter College, Urban Policy & Planning, New York, NY 10065, USA

3. Forest Inventory and Analysis, U.S. Forest Service, Rocky Mountain Research Station, Riverdale, UT 84405, USA

4. College of Architecture and Planning, University of Colorado Denver, University of Colorado Denver, Denver, CO, 80202, USA

5. Geospatial Technology and Applications Center, U.S. Forest Service, National Forest System, Salt Lake City, UT 84138, USA

6. Spatial Analysis Laboratory, Rubenstein School of Environment & Natural Resources, University of Vermont, Burlington, VT 05405, USA (deceased)

corresponding author(s): Lucila M. Corro ([lcorro@usgs.gov](mailto:lcorro@usgs.gov))

Supplemental File 1. The supplemental document provides a comprehensive overview of the results derived from the random forest tree canopy correction model across 71 urban areas in the United States. For each urban locale, a concise summary presents the outcomes, which are visualized through scatter plots comparing the predicted tree canopy cover (TCC) against the actual cover derived from high-resolution imagery and comparing the native National Land Cover Database (NLCD) ^1^ tree canopy cover against the actual cover derived from high-resolution imagery. To offer a deeper spatial context, the document includes maps illustrating the native NLCD tree canopy cover, the upscaled high-resolution derived tree canopy cover, and the newly predicted (enhanced) tree canopy cover.

Annapolis, Maryland (city)

Coefficient of determination (R-Squared) 0.6346

Root mean square error (RMSE): 21.3763

Mean absolute error (MAE): 16.4175


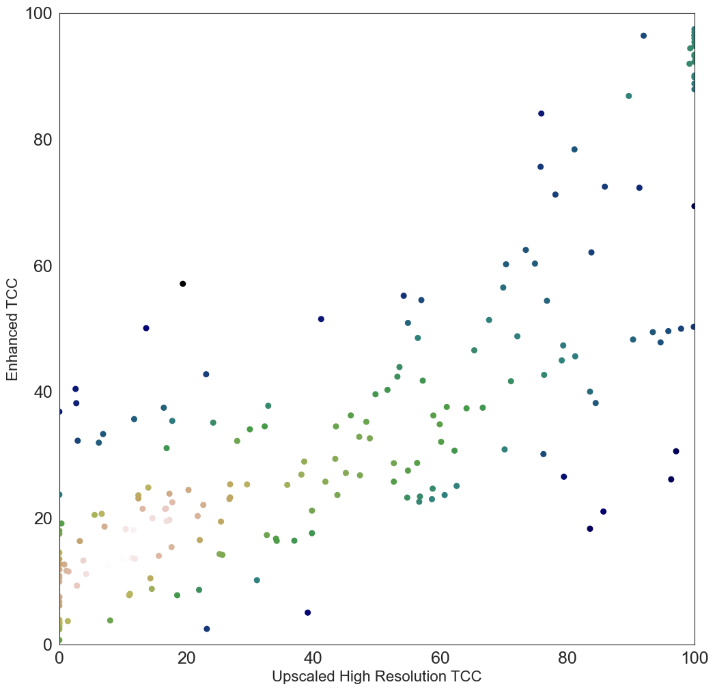

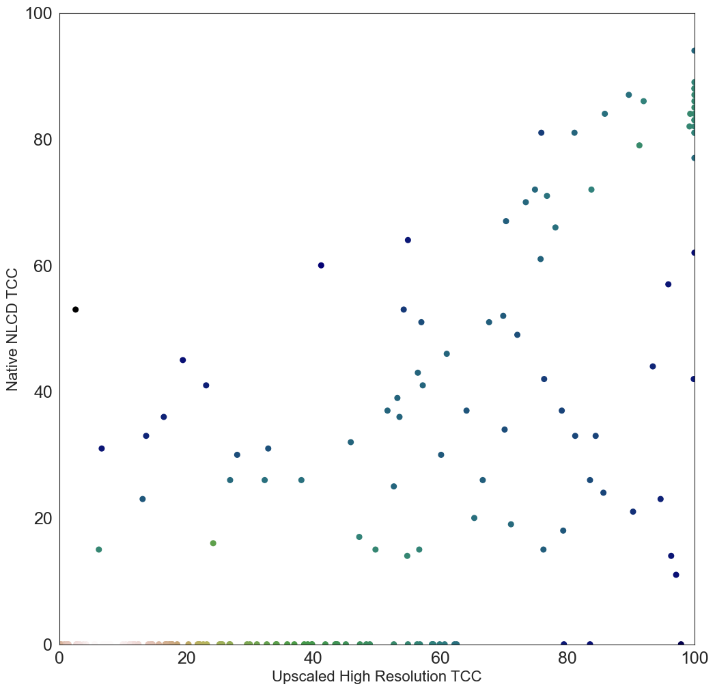


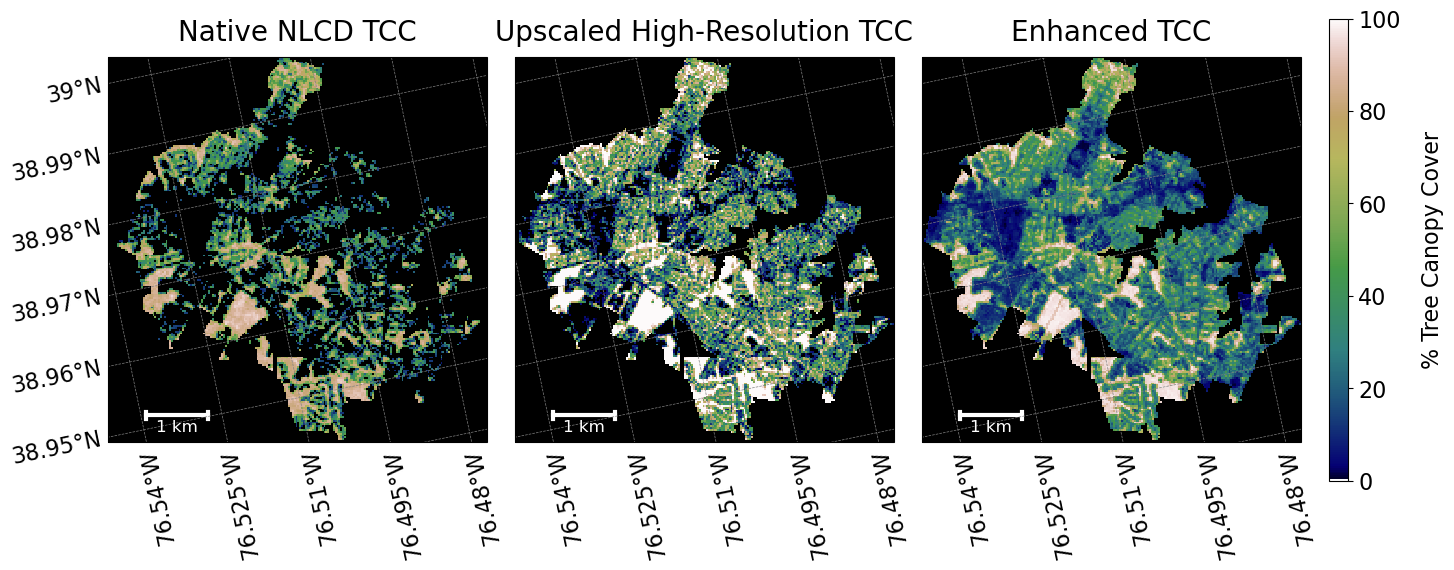


Anne Arundel County, Maryland (Baltimore urban area)

R-Squared 0.7299

RMSE: 19.9313

MAE: 14.3215


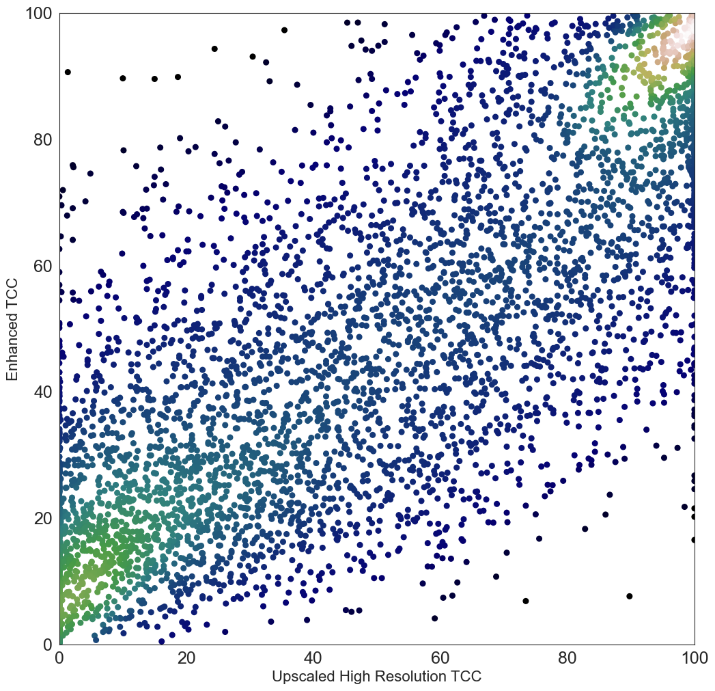

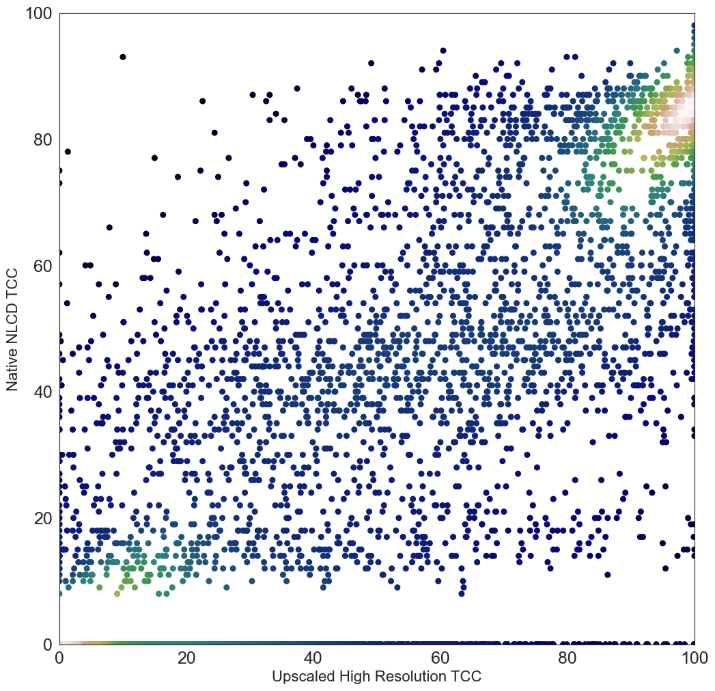


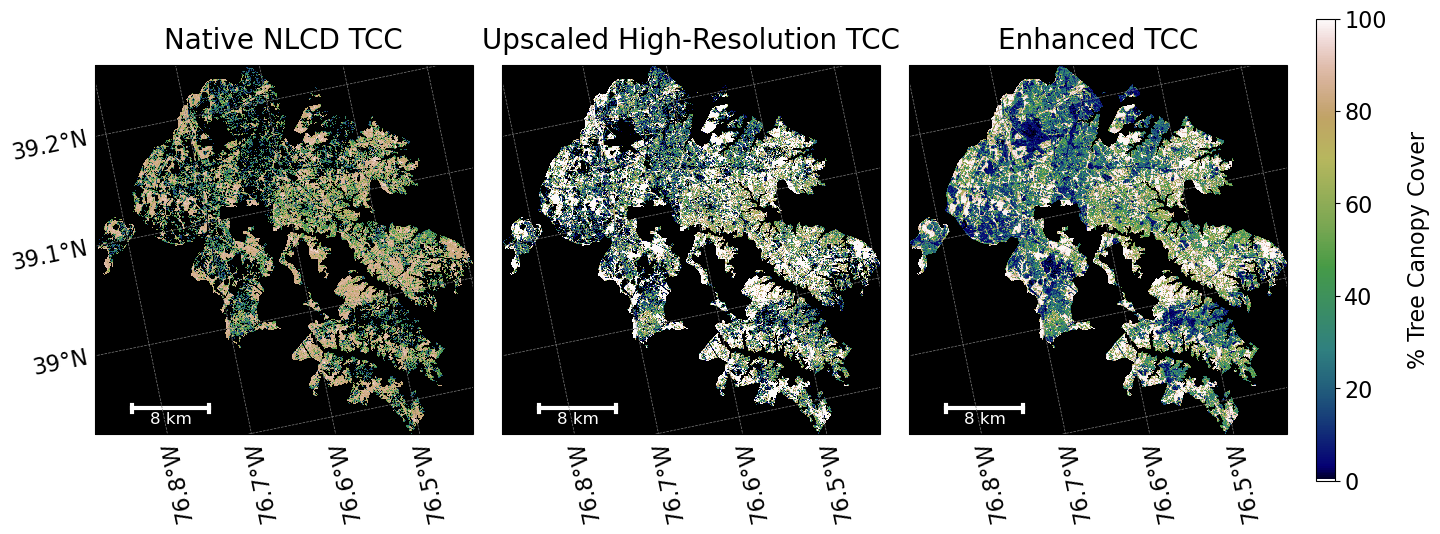


Austin, Texas (urban area)

R-Squared 0.7182

RMSE: 19.095

MAE: 14.0842


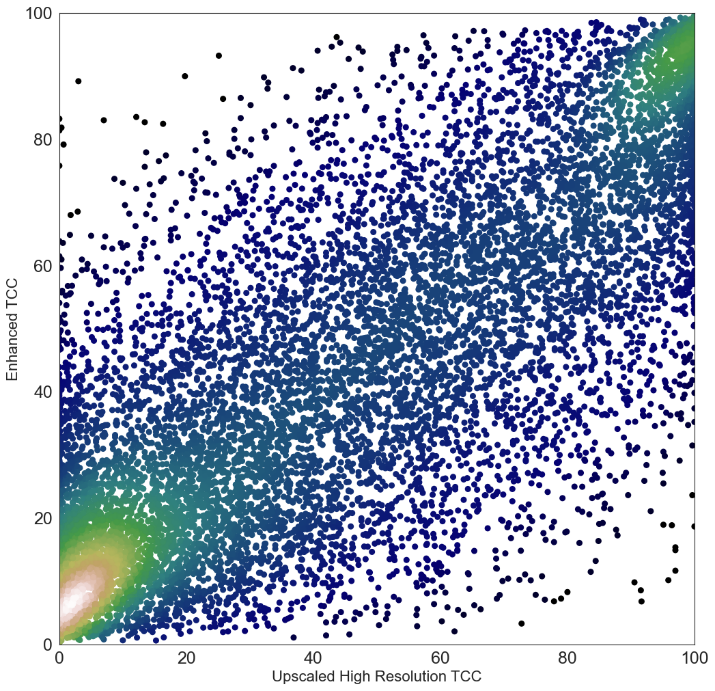

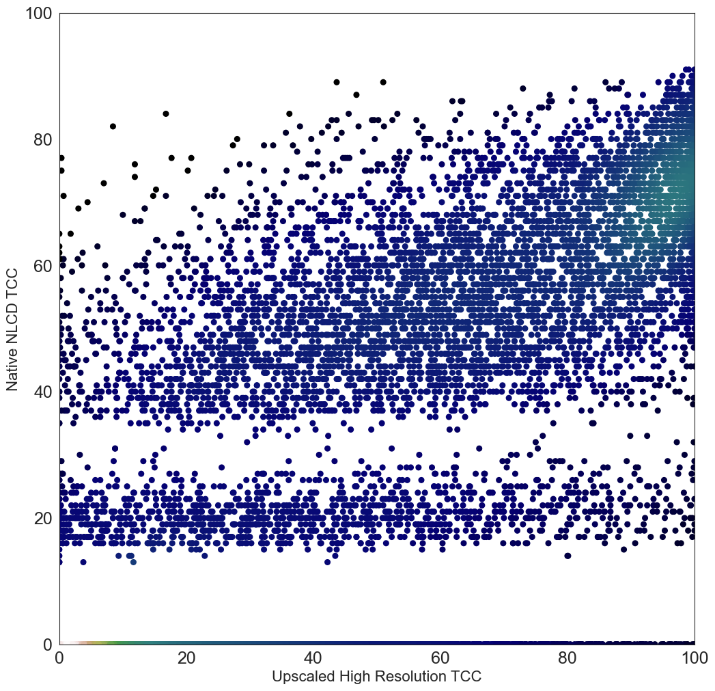


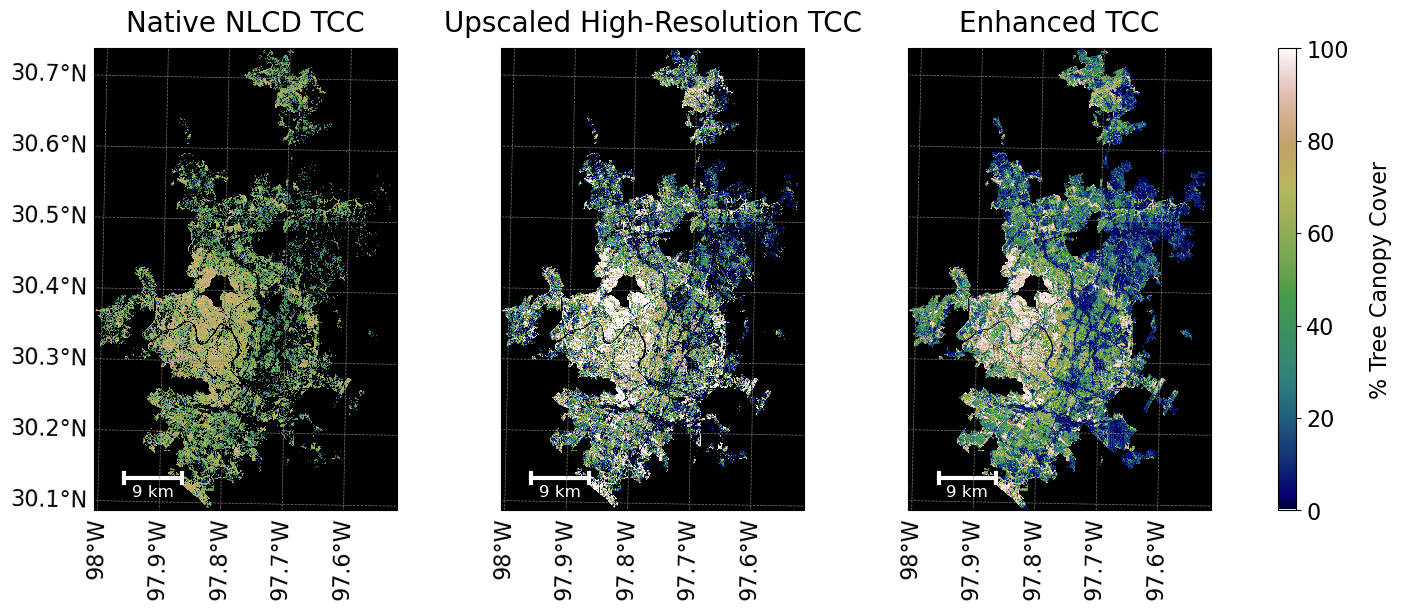


Avalon, California (urban area)

R-Squared 0.4822

RMSE: 16.9455

MAE: 12.9007


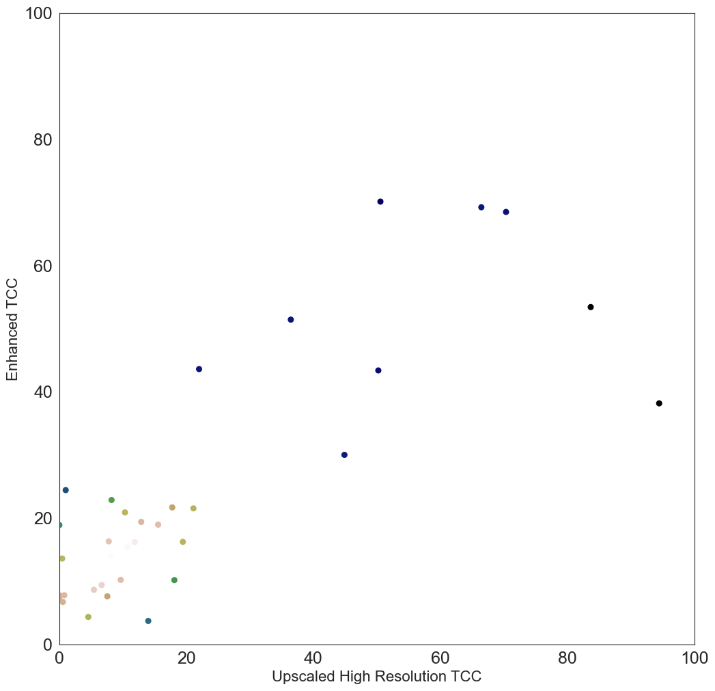

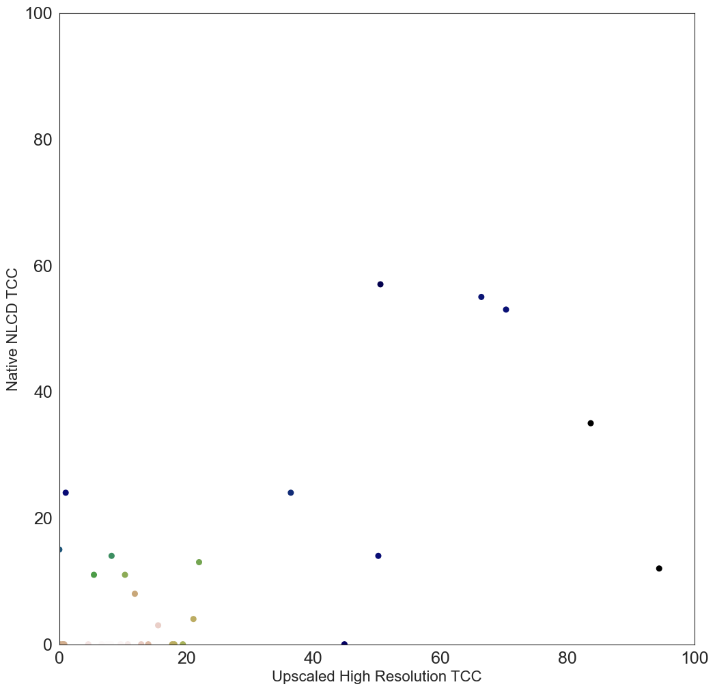


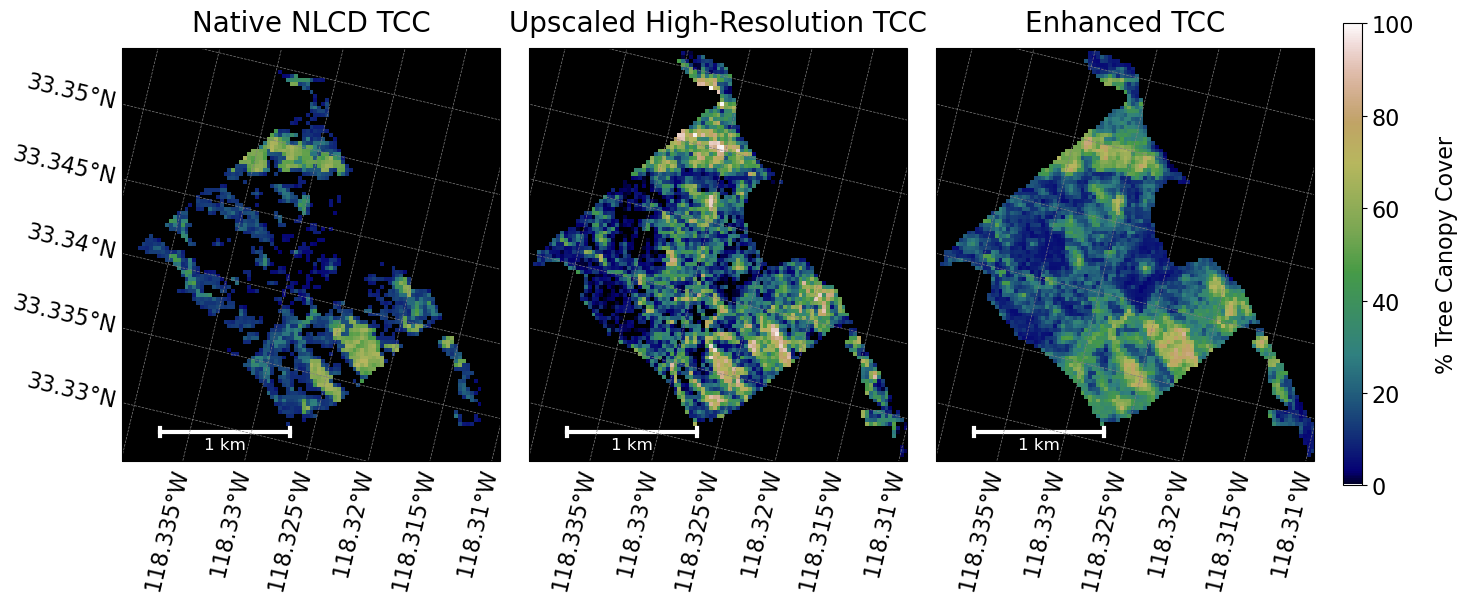


Baltimore, Maryland (City)

R-Squared 0.6644

RMSE: 18.1819

MAE: 12.8504


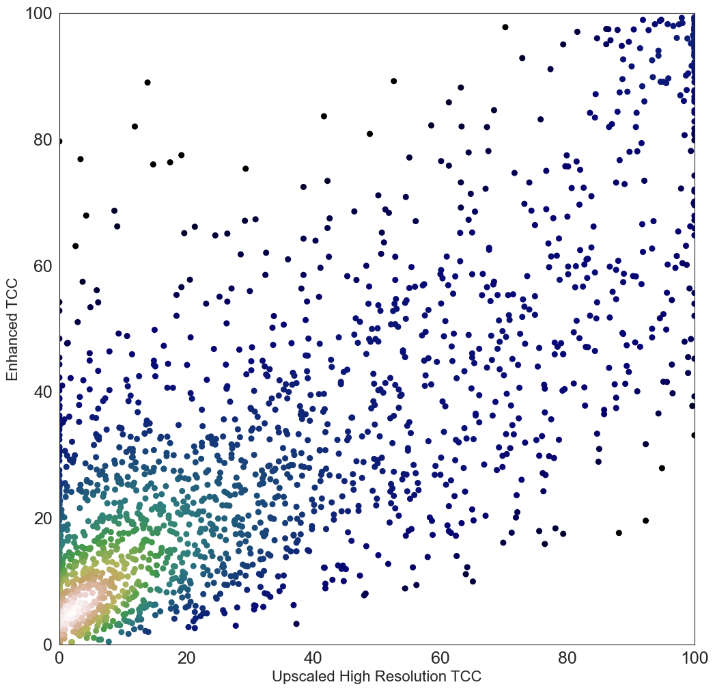

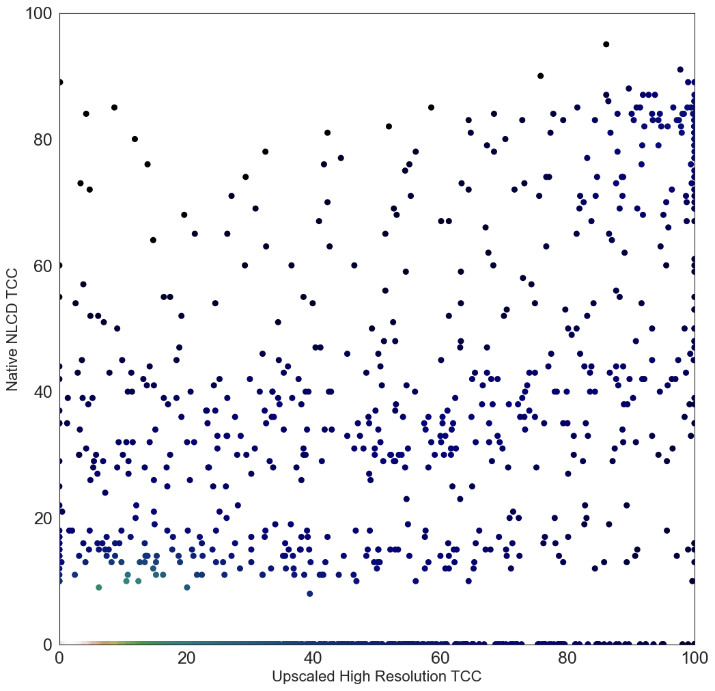


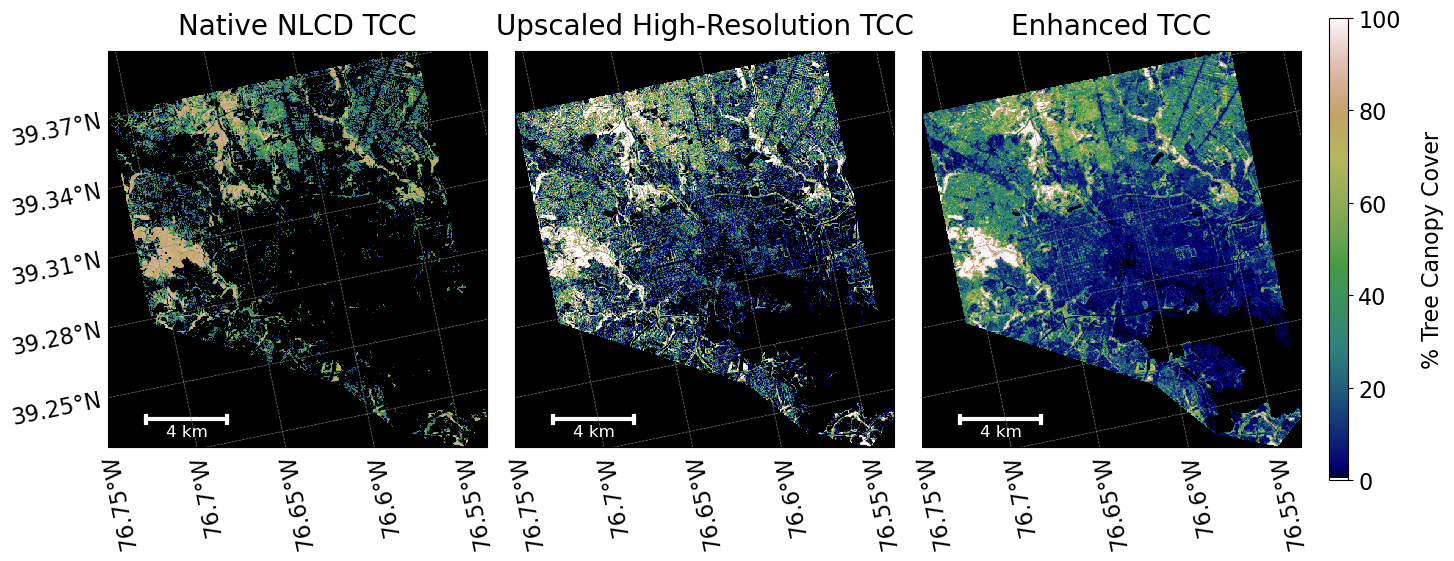


Baltimore, Maryland (county, urban area)

R-Squared 0.7097

RMSE: 20.1782

MAE: 14.5946


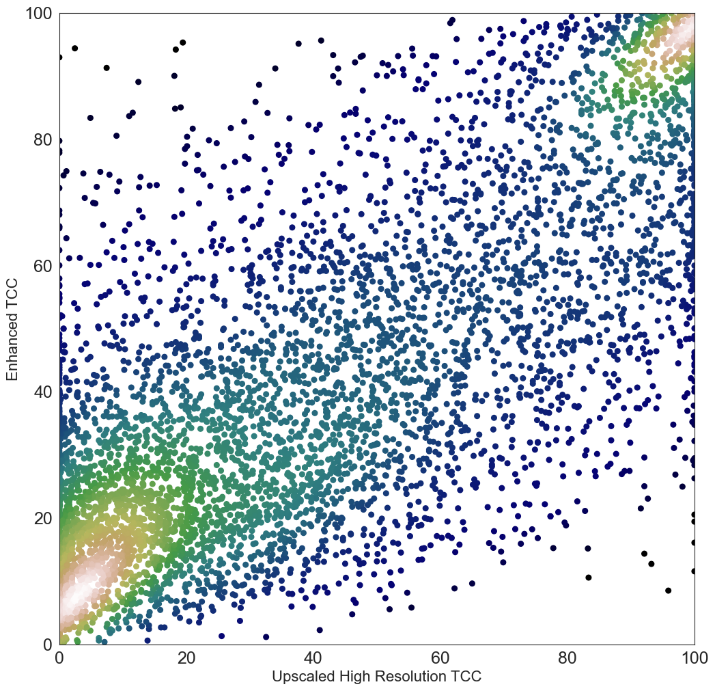

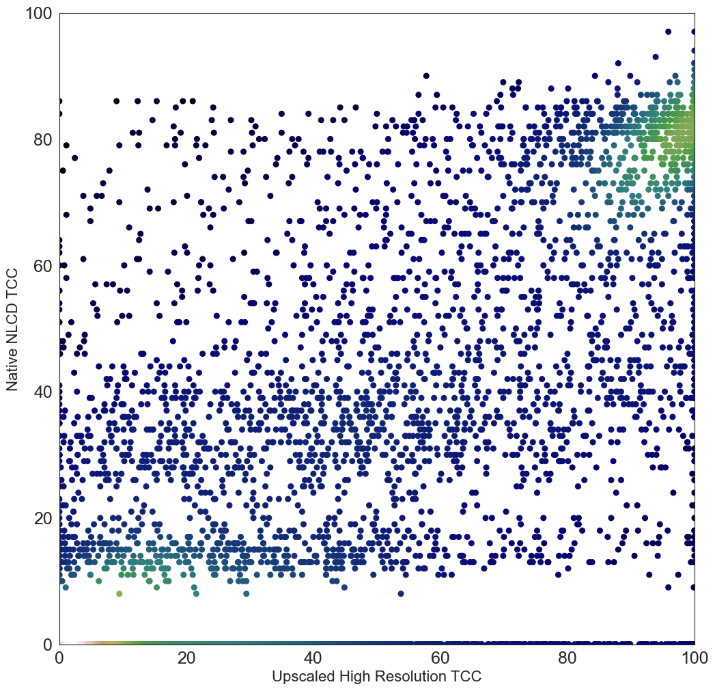


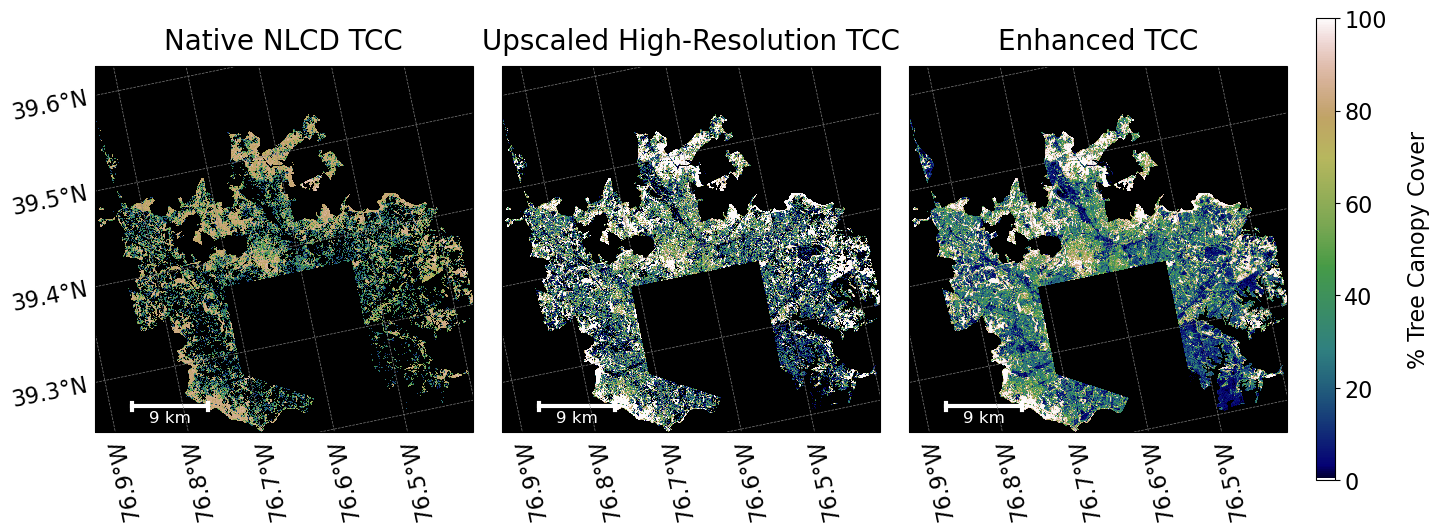


Baltimore, Maryland (urban area)

R-Squared 0.7291

RMSE: 20.4044

MAE: 14.6135


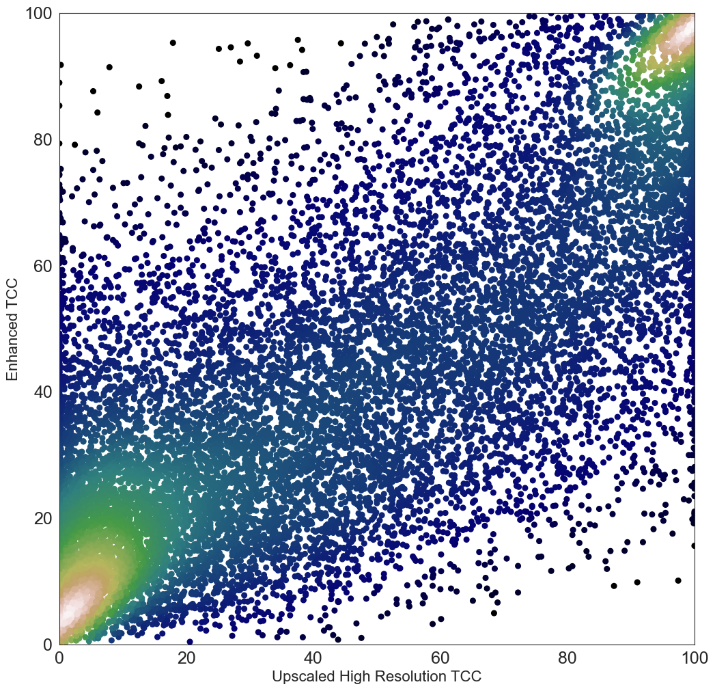

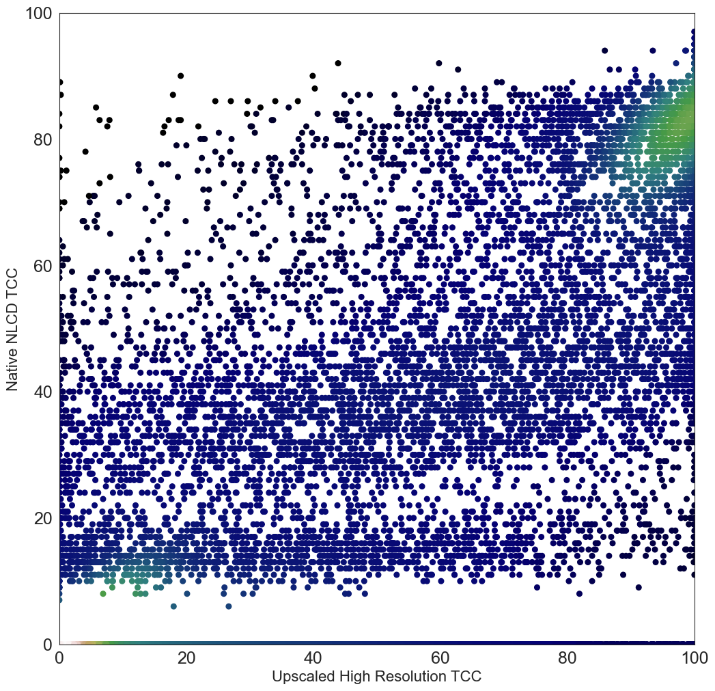


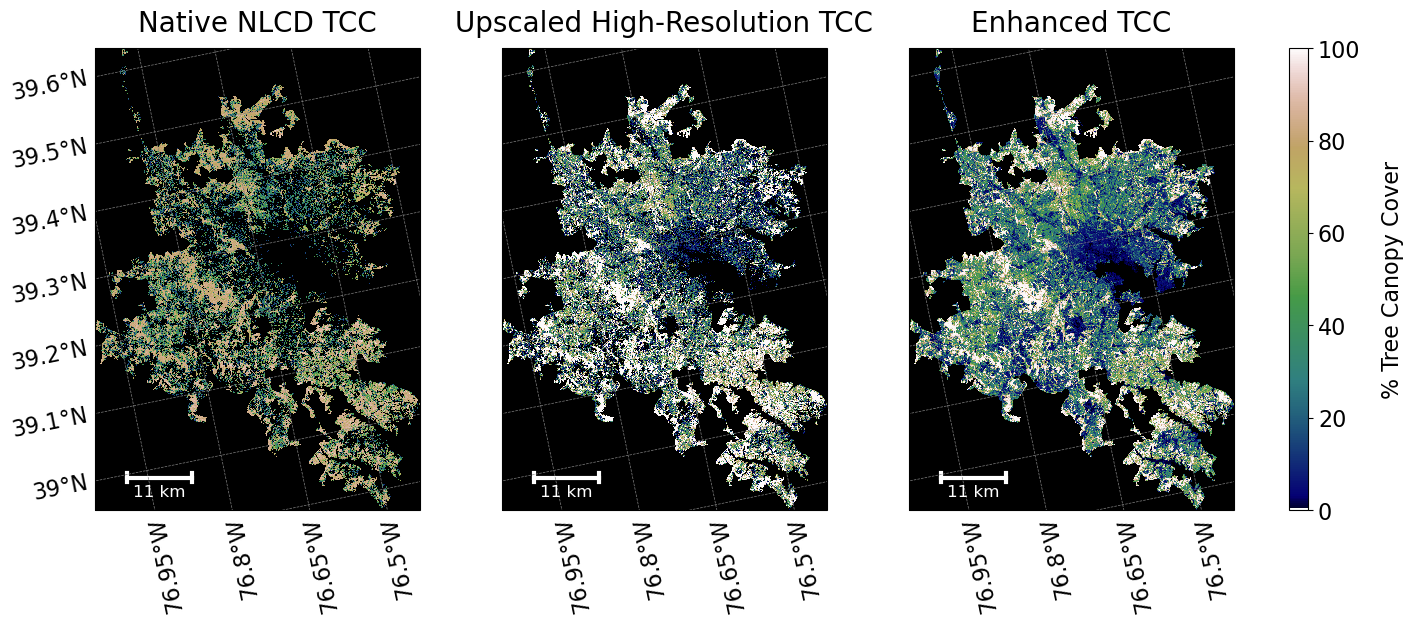


Berkeley County, West Virginia (Hagerstown, Maryland, urban area)

R-Squared 0.7916

RMSE: 17.5294

MAE: 12.543


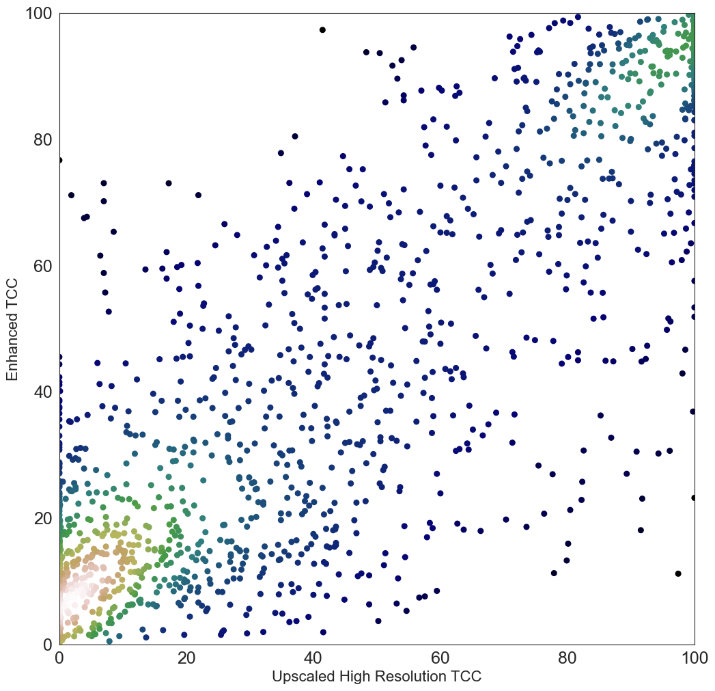

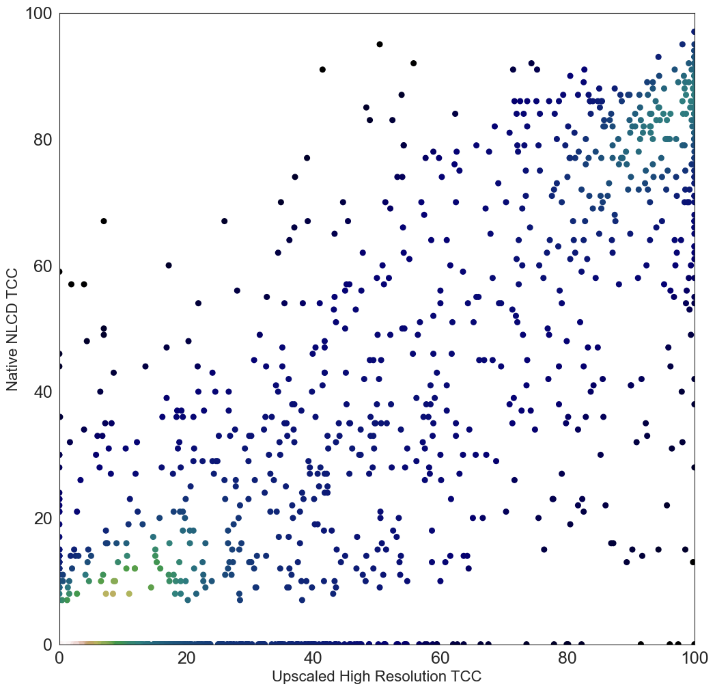


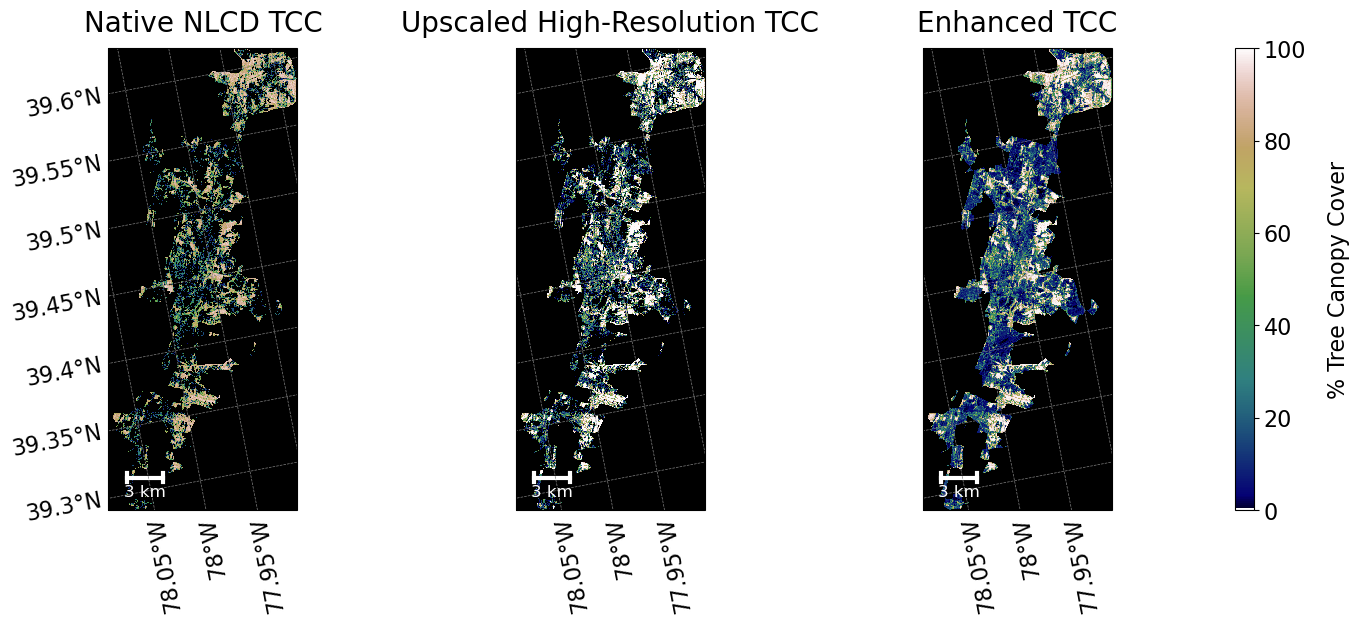


Birmingham, Alabama (urban area)

R-Squared 0.7536

RMSE: 19.8008

MAE: 13.7809


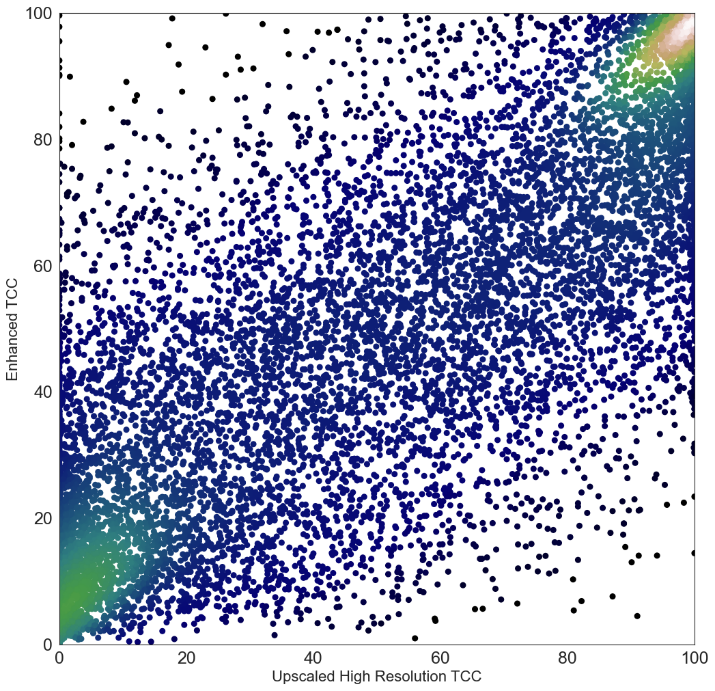

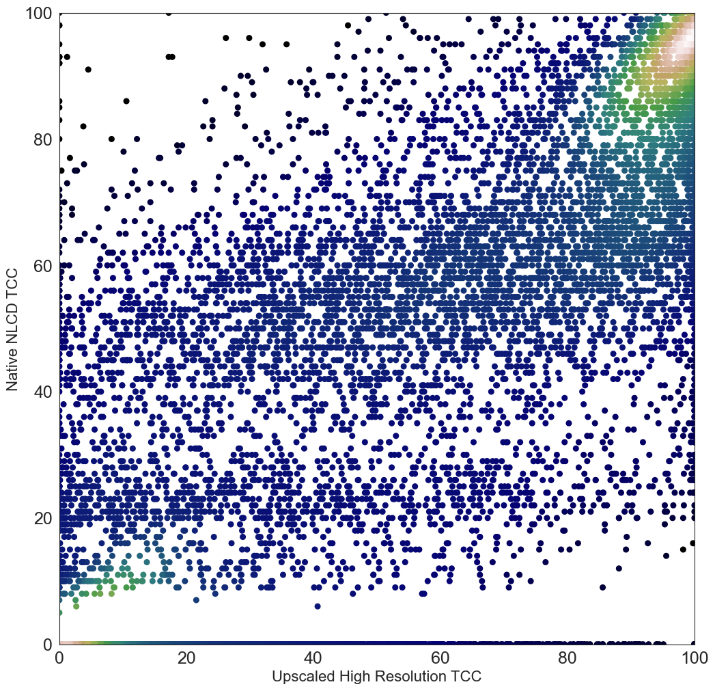


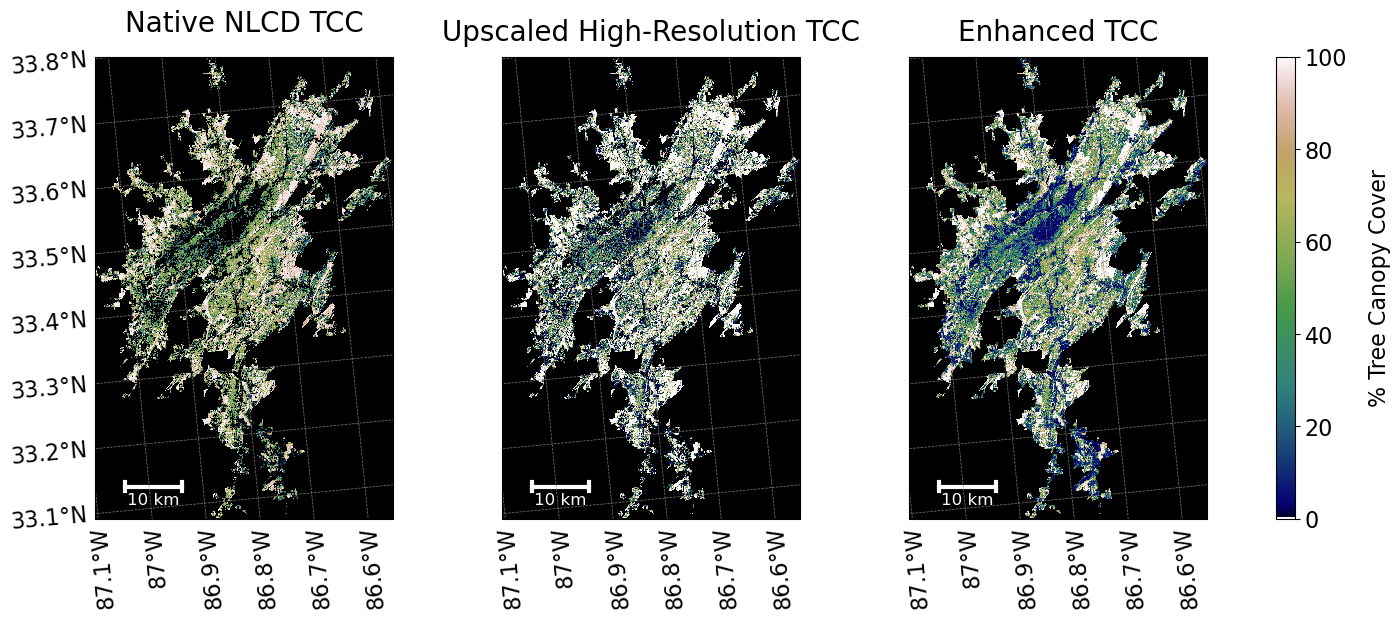


Boise, Idaho (city)

R-Squared 0.5349

RMSE: 12.8943

MAE: 8.924


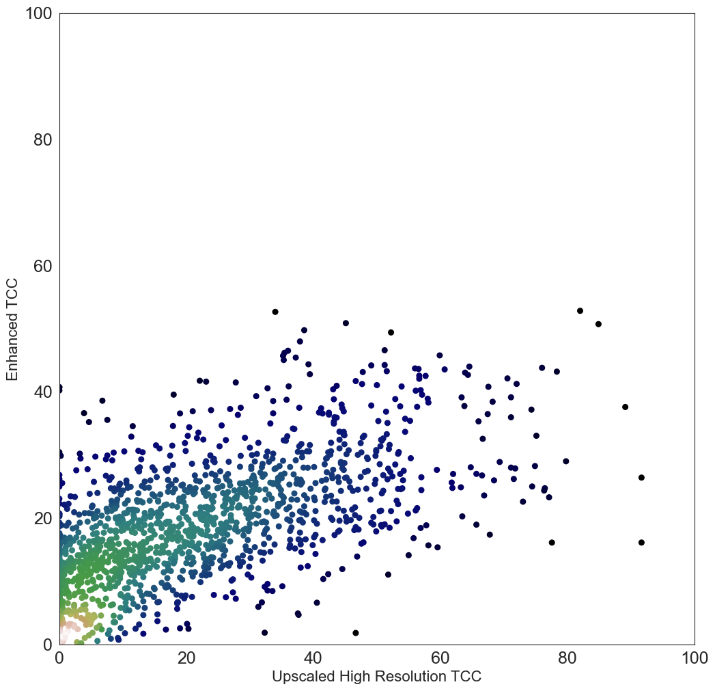

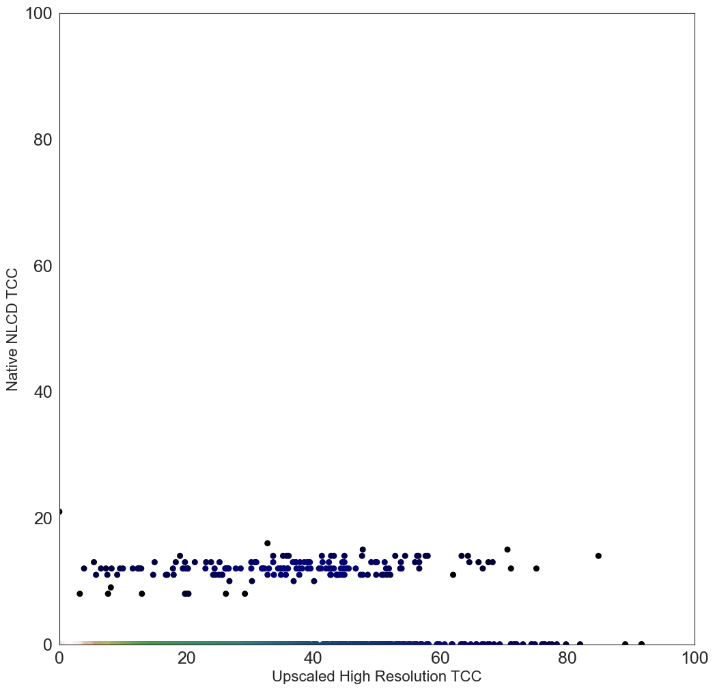


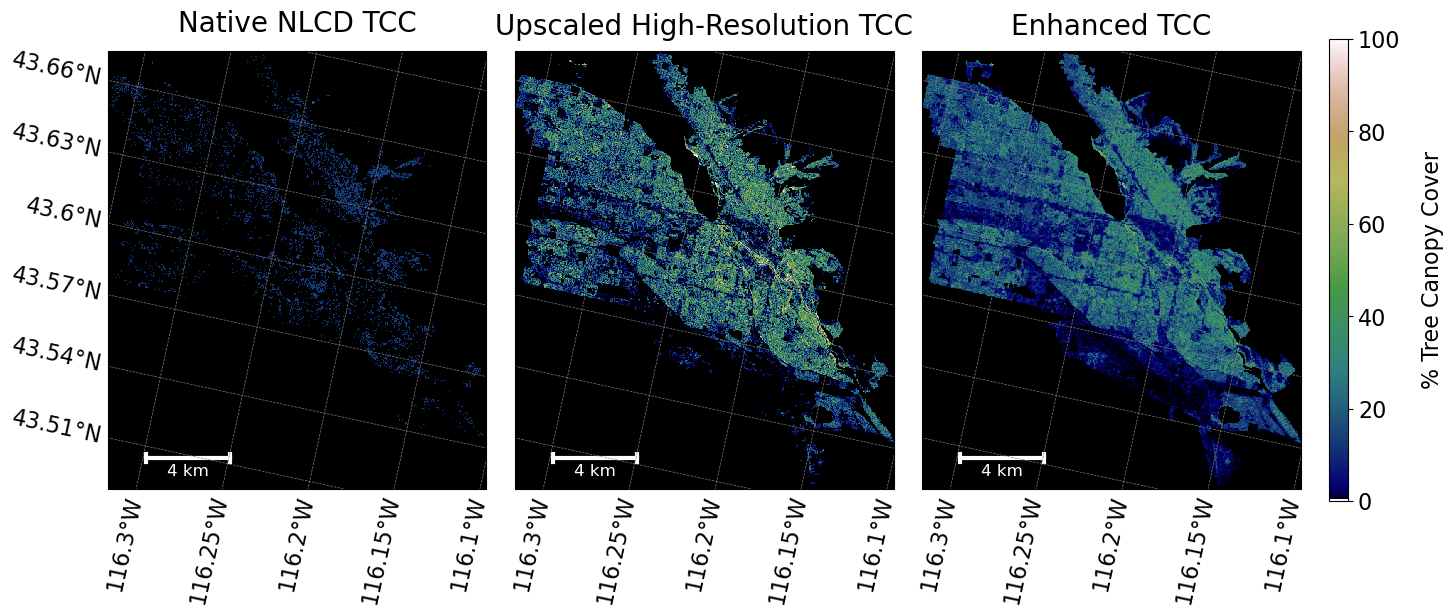


Boston, Massachusetts (city)

R-Squared 0.6479

RMSE: 17.5406

MAE: 12.4207


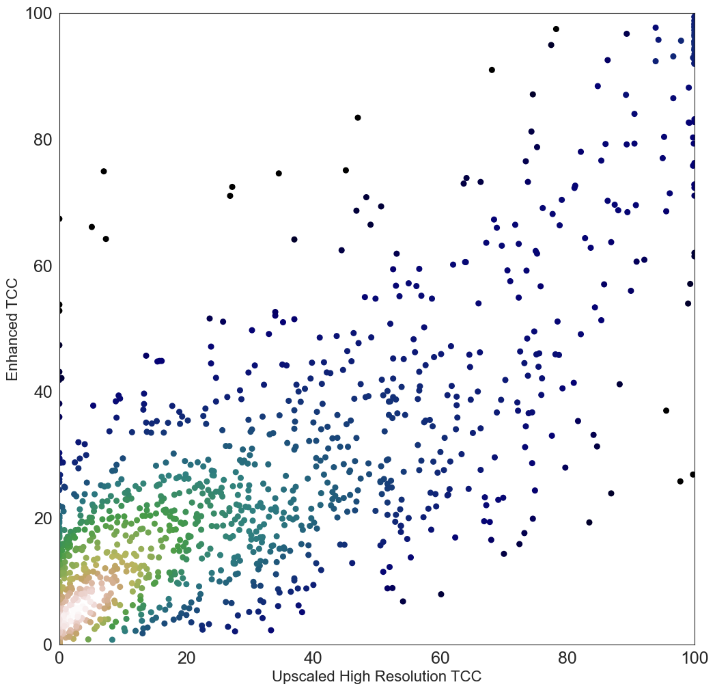

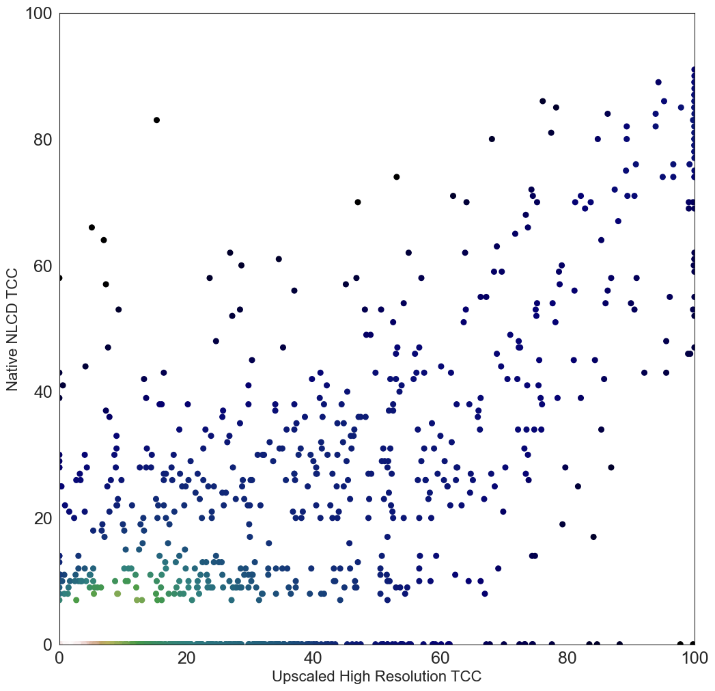


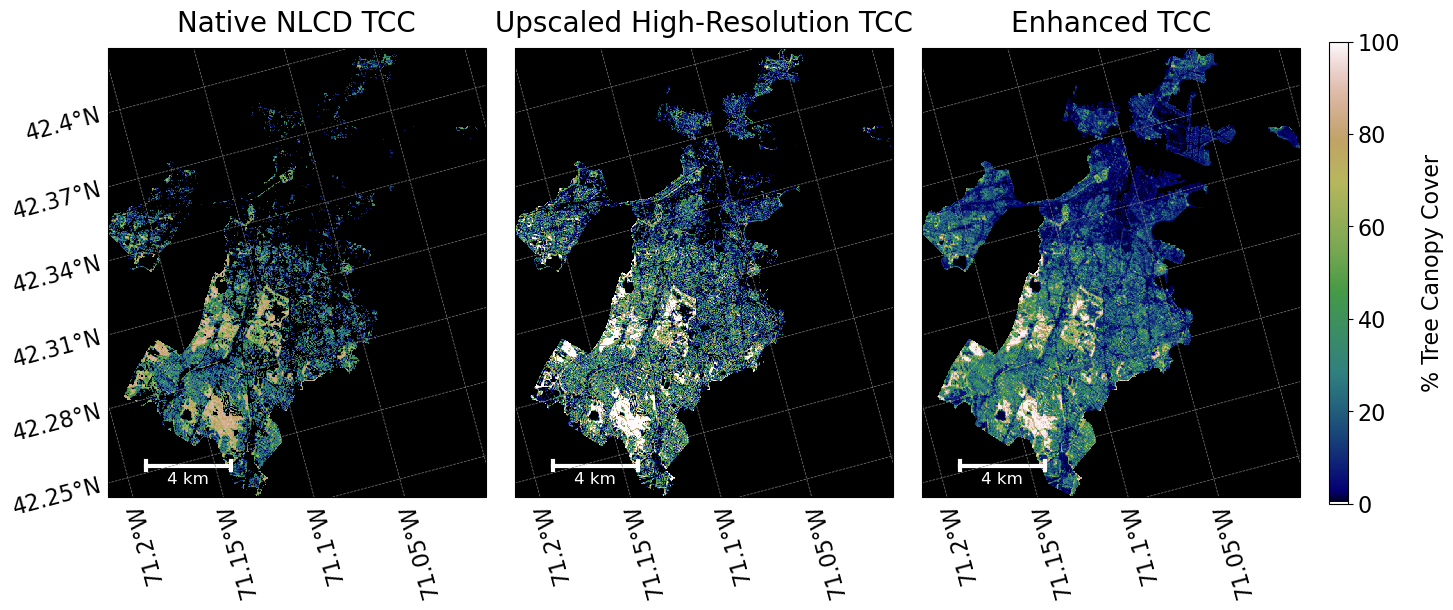


Brownsville, Texas (urban area)

R-Squared 0.5586

RMSE: 15.9994

MAE: 11.2125


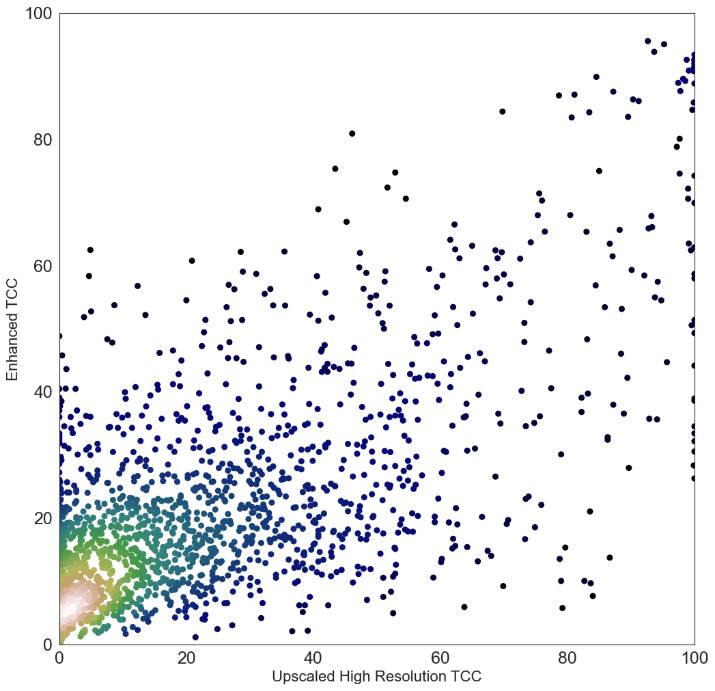

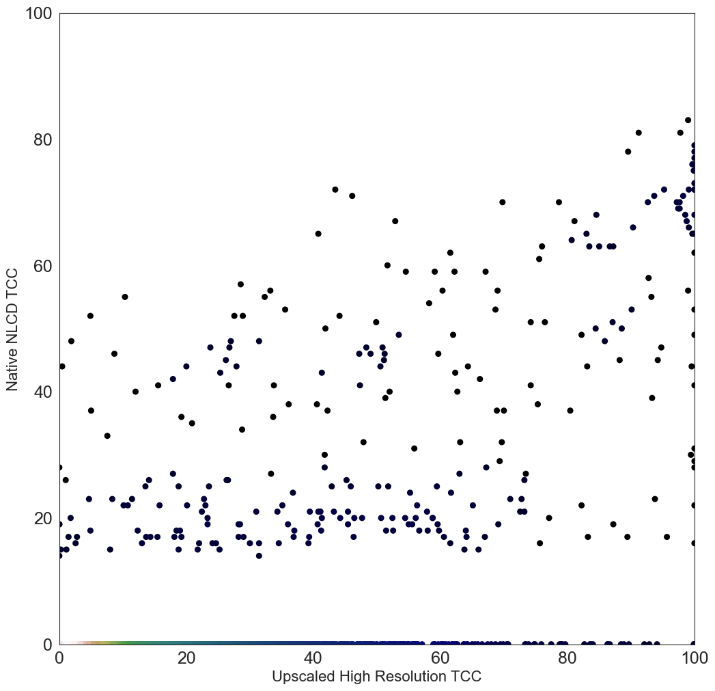


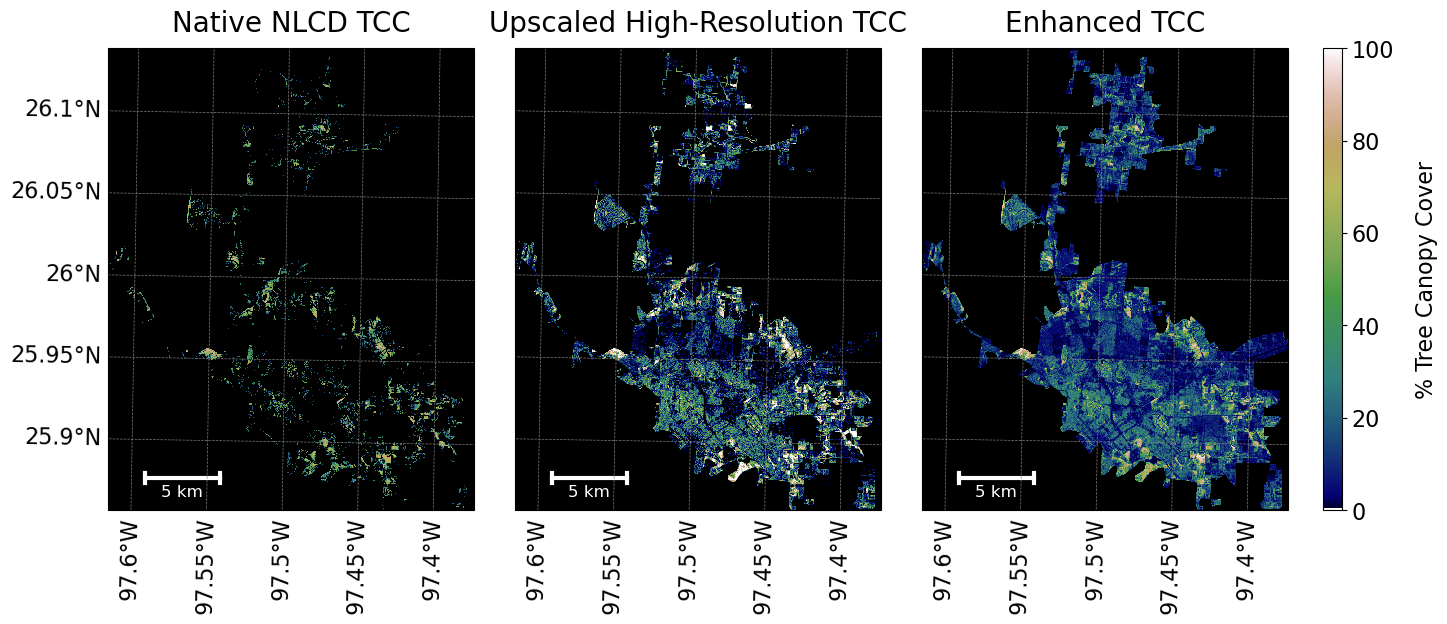


Burlington, Vermont (city)

R-Squared 0.5935

RMSE: 21.9462

MAE: 16.3795


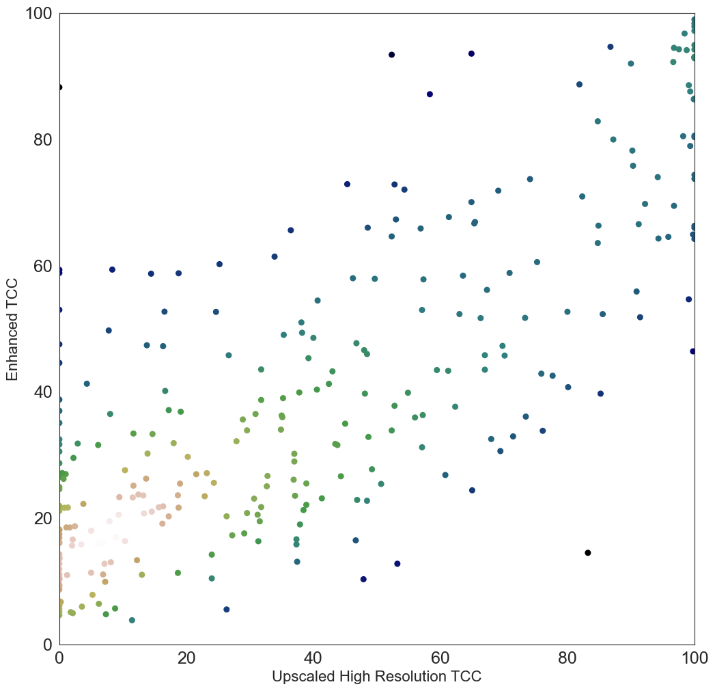

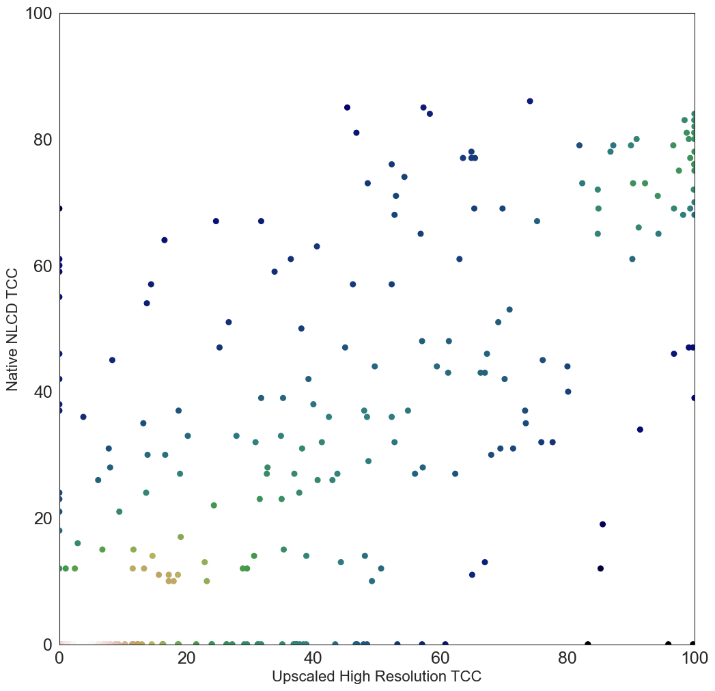


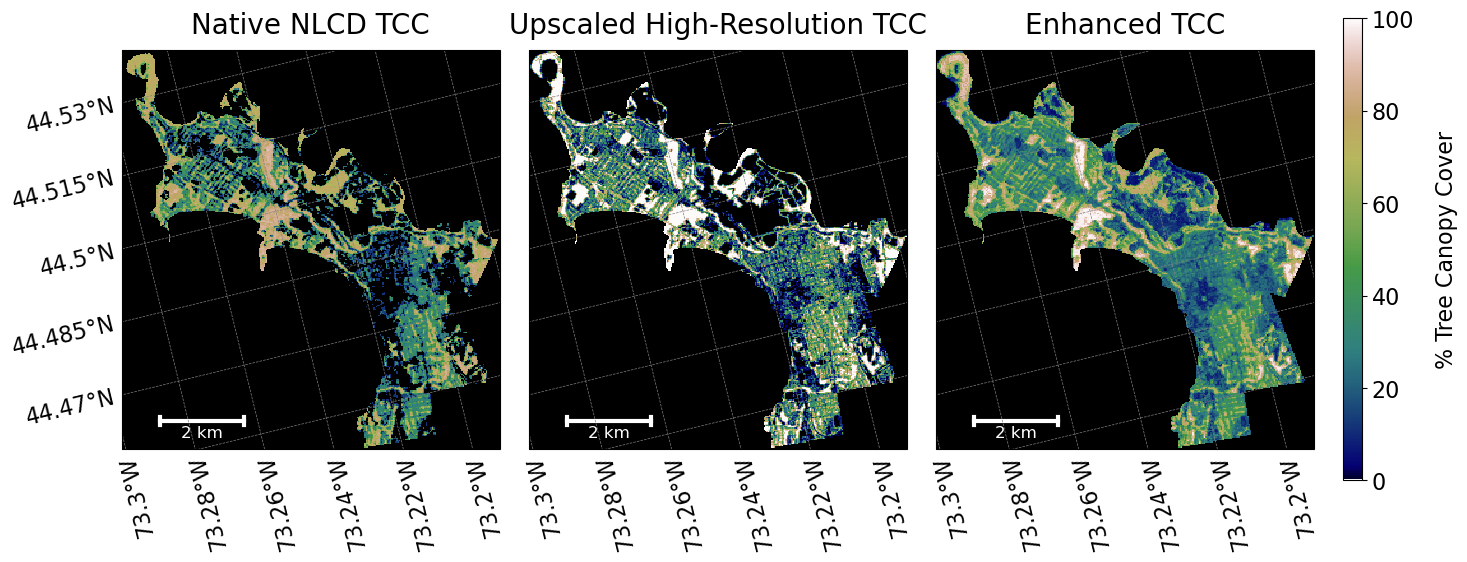


Cambridge, Massachusetts (city)

R-Squared 0.4972

RMSE: 18.6104

MAE: 13.2554


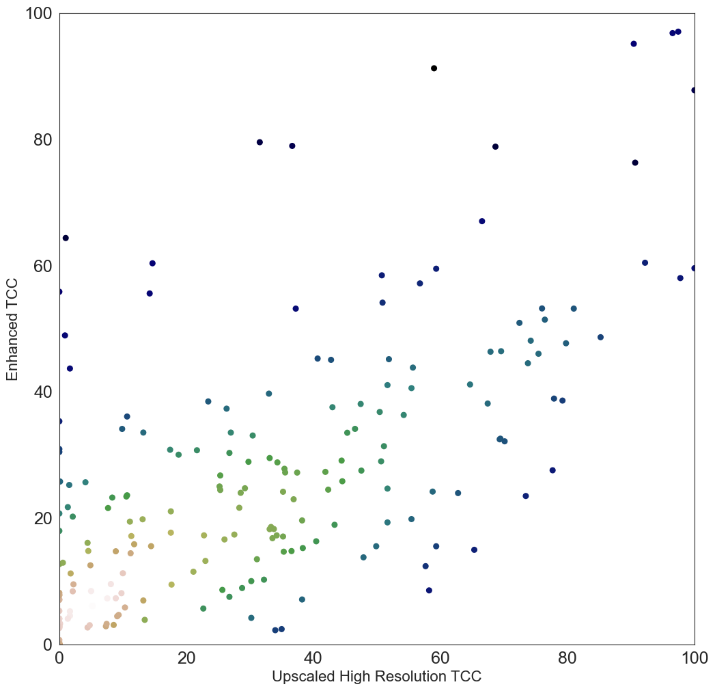

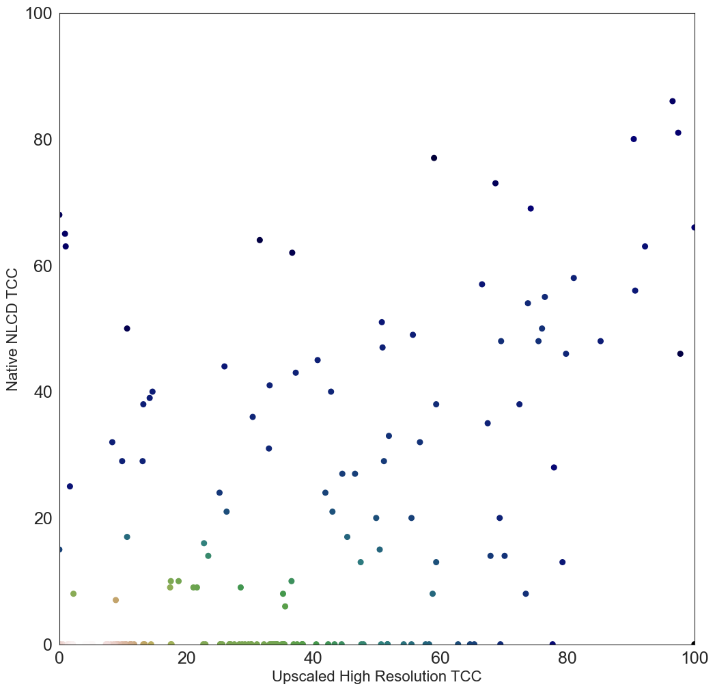


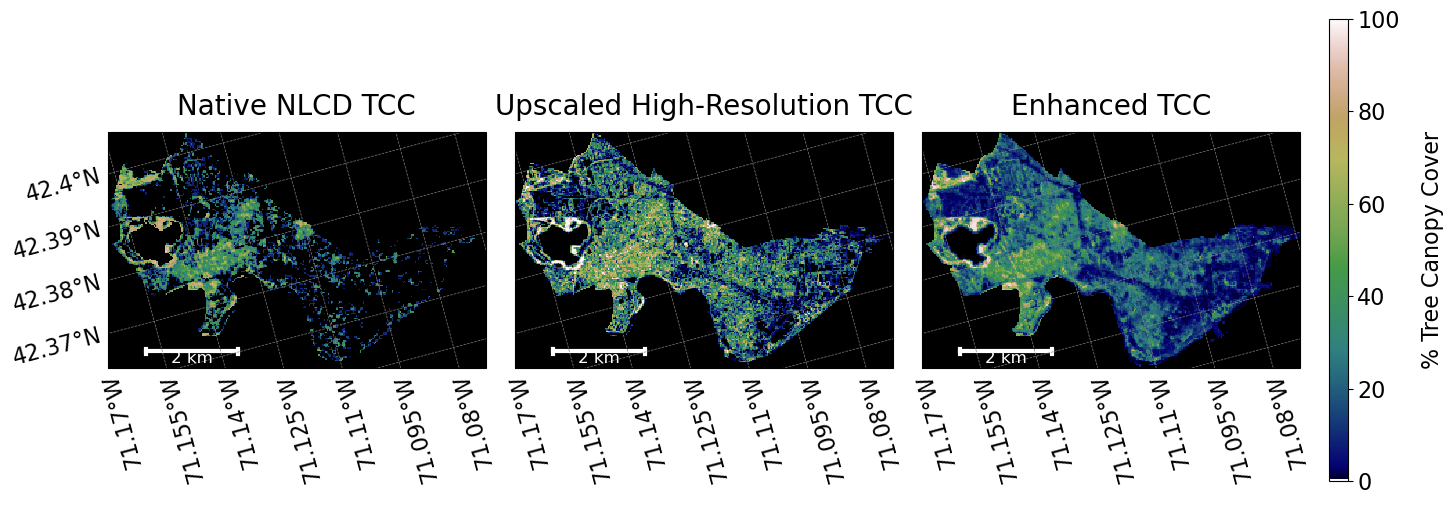


Charles Town-Ranson, West Virginia (Jefferson County urban area)

R-Squared 0.6981

RMSE: 17.2432

MAE: 12.1365


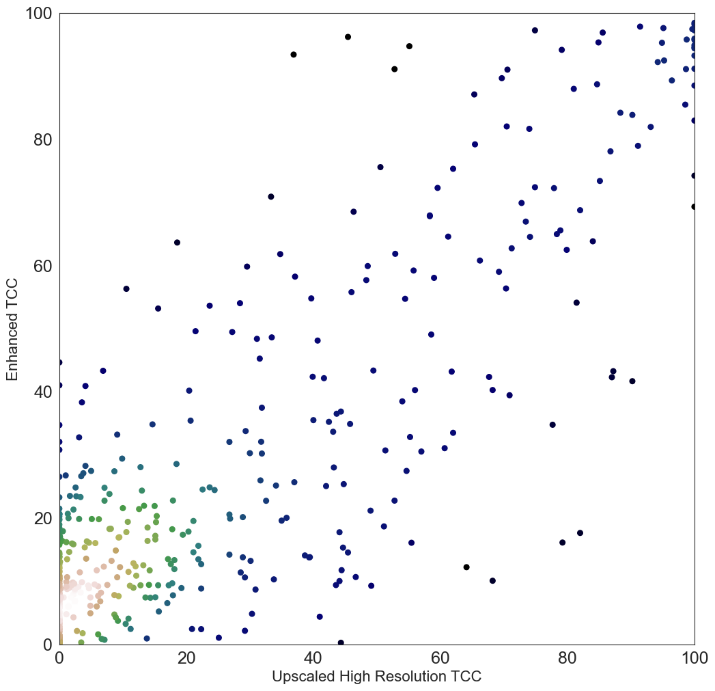

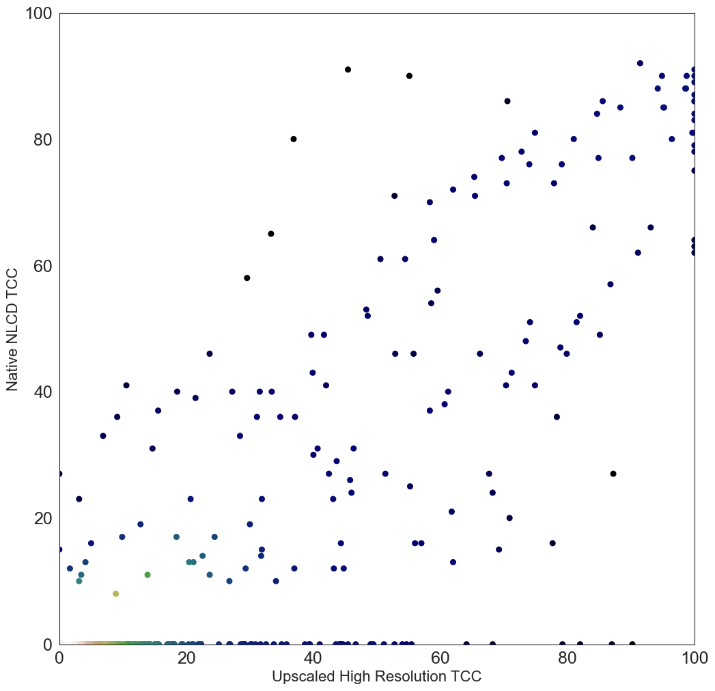


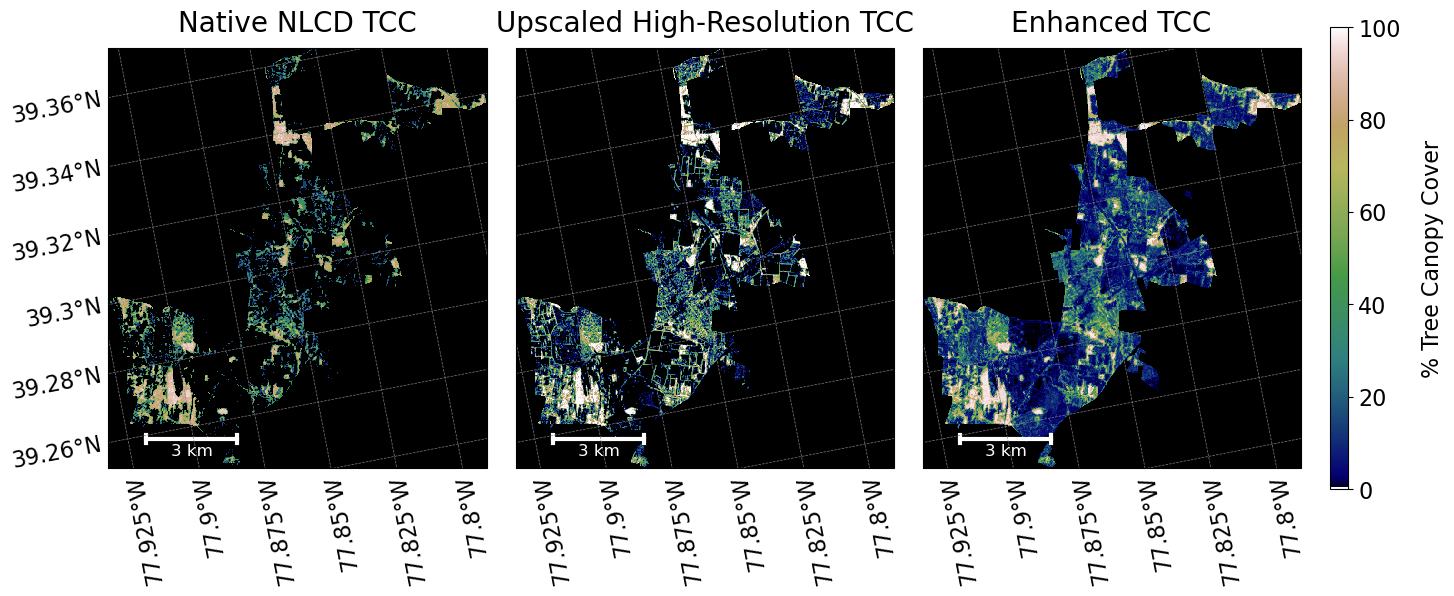


Charlotte, North Carolina (Mecklenburg County urban area)

R-Squared 0.72

RMSE: 20.3993

MAE: 14.7496


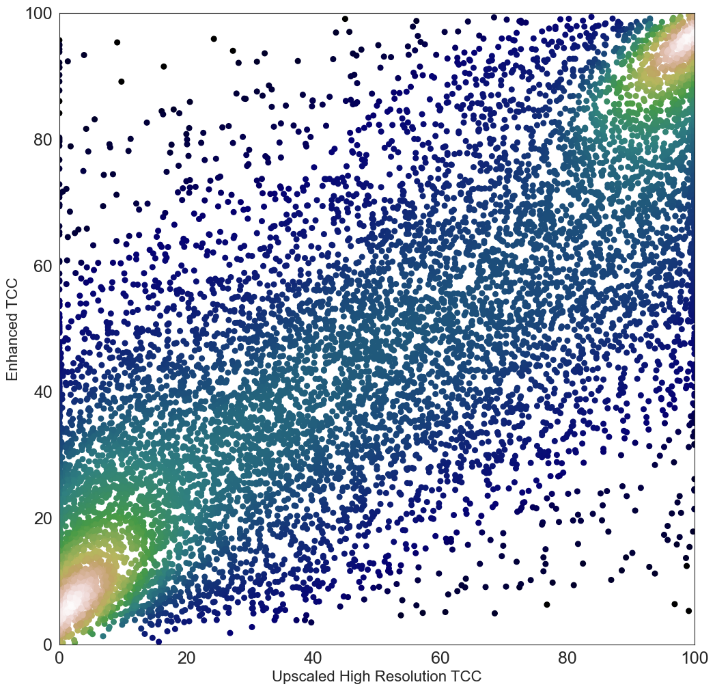

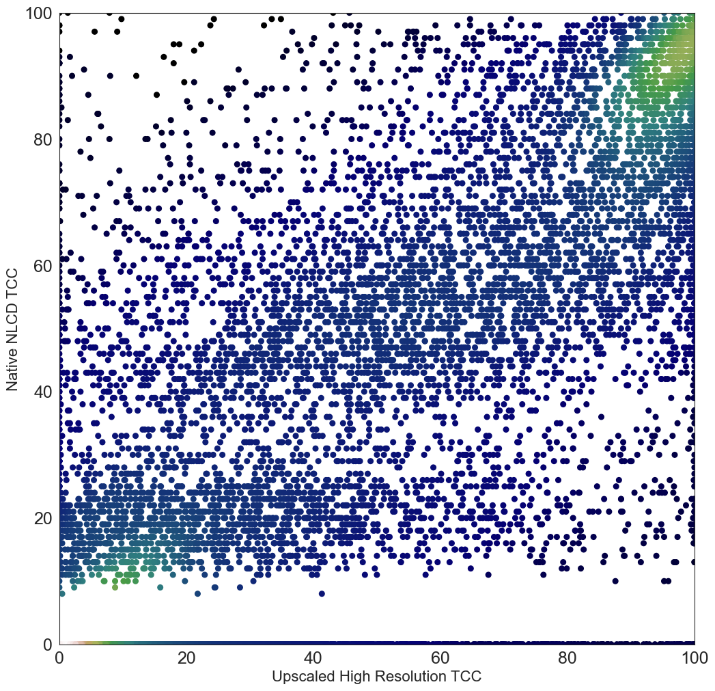


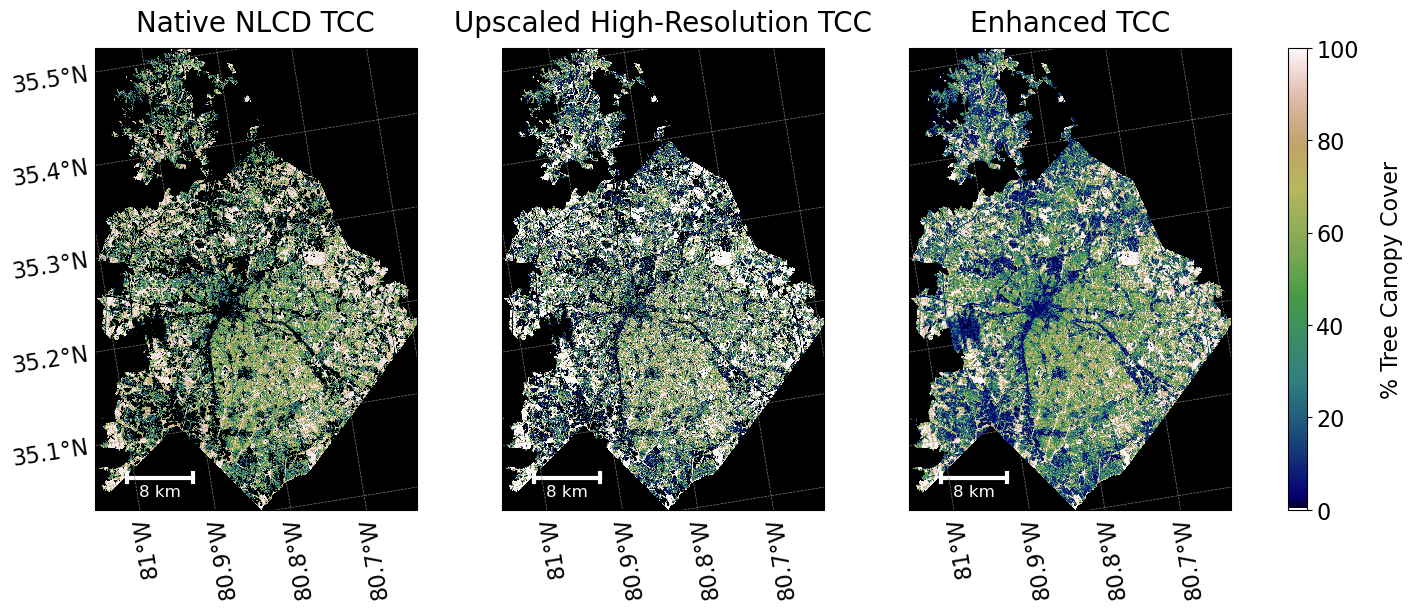


Chicago, Illinois-Indiana (urban area)

R-Squared 0.6547

RMSE: 17.7246

MAE: 12.5189


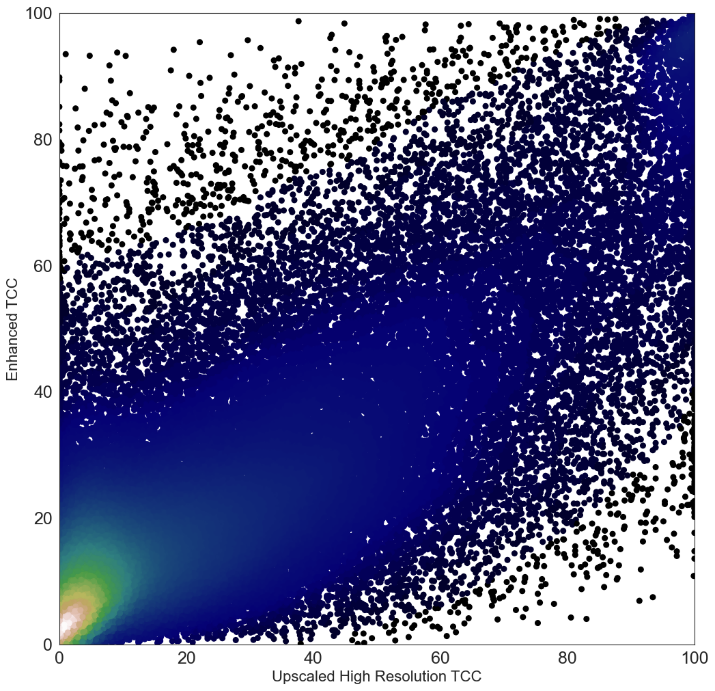

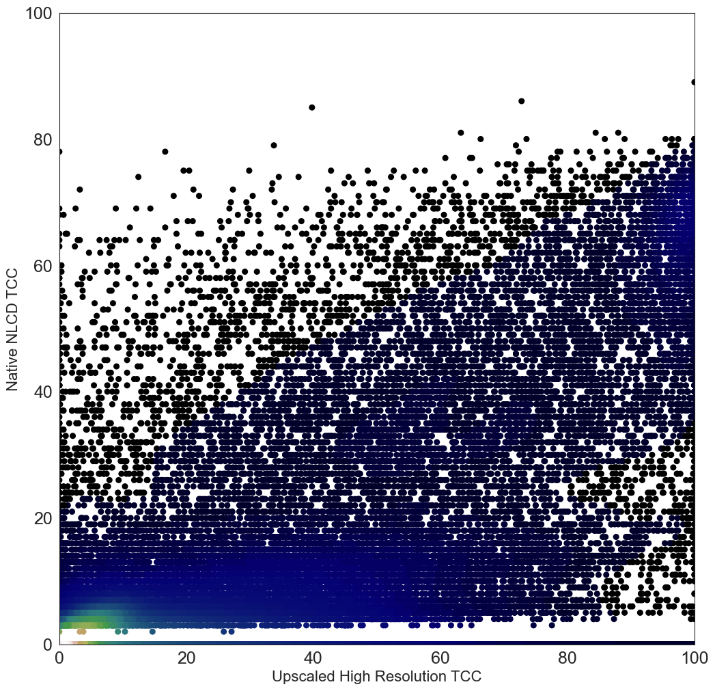


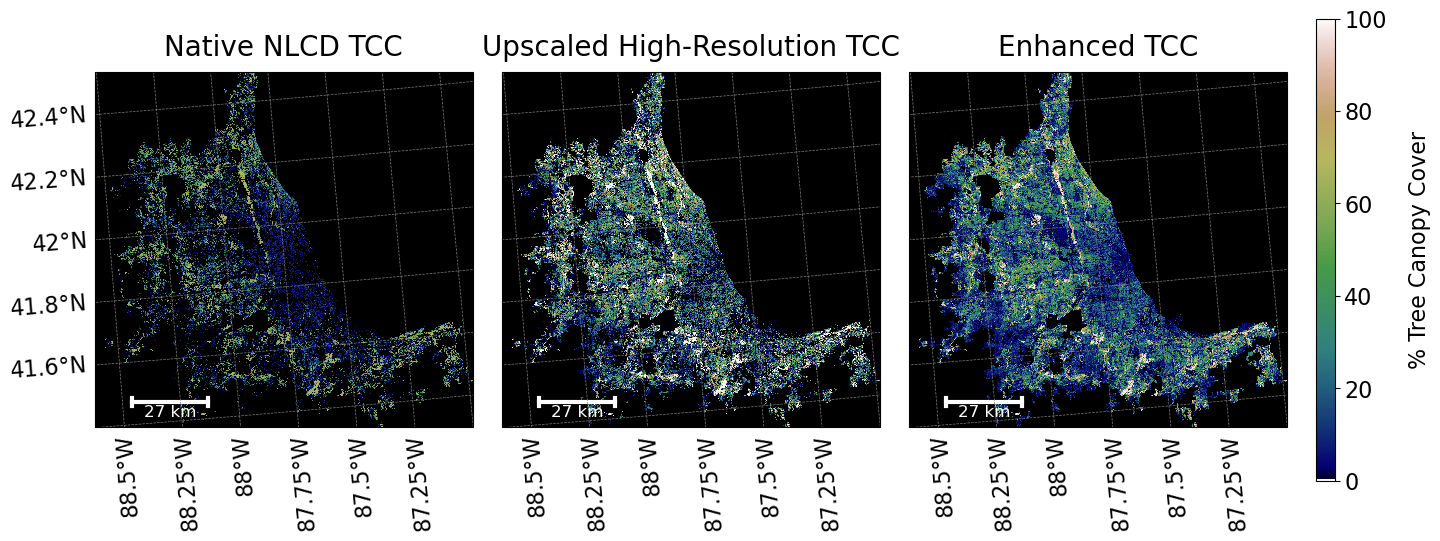


Cleveland, Ohio (urban area)

R-Squared 0.7734

RMSE: 17.5355

MAE: 12.391


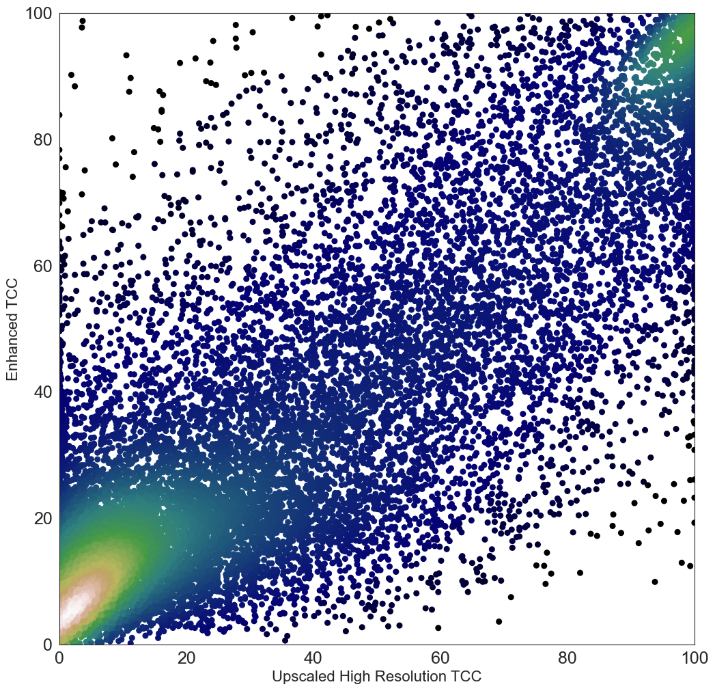

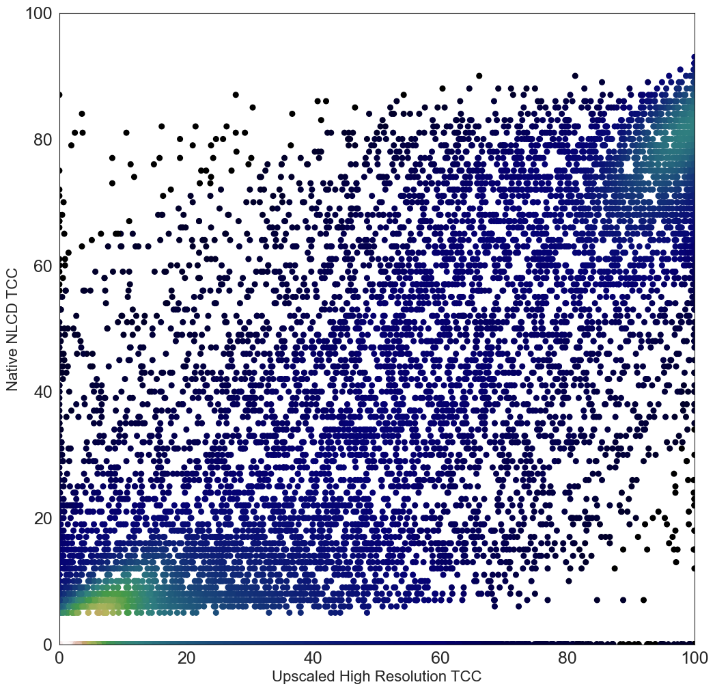


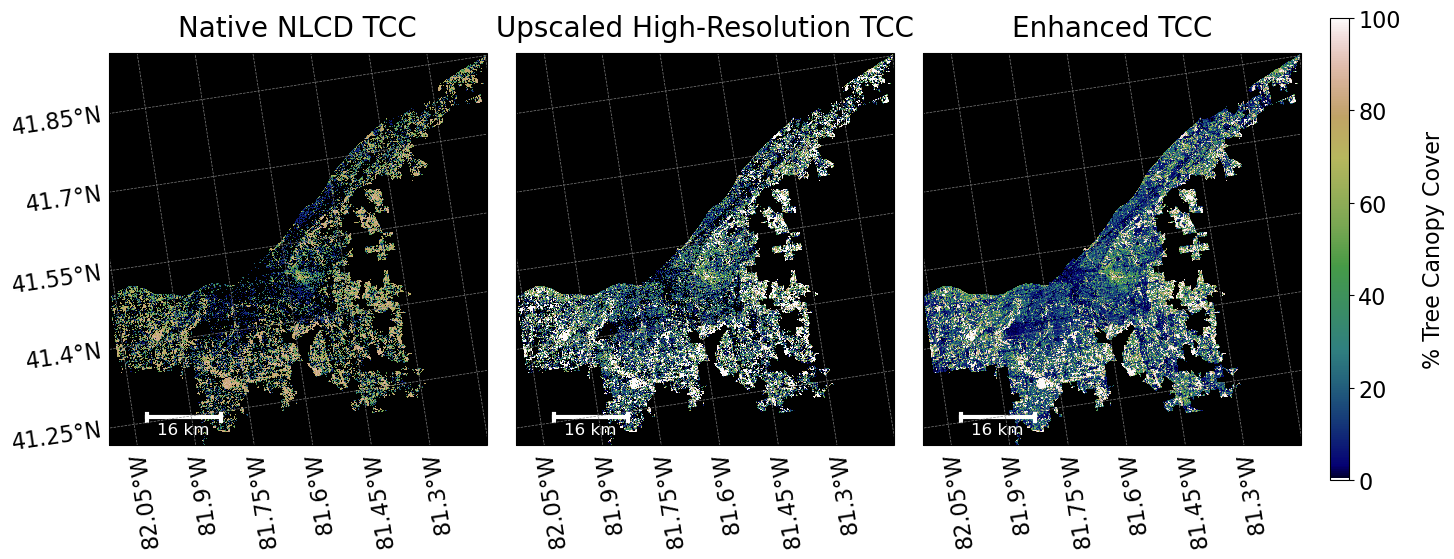


Cloverdale, California (urban area)

R-Squared 0.6799

RMSE: 17.152

MAE: 12.6117


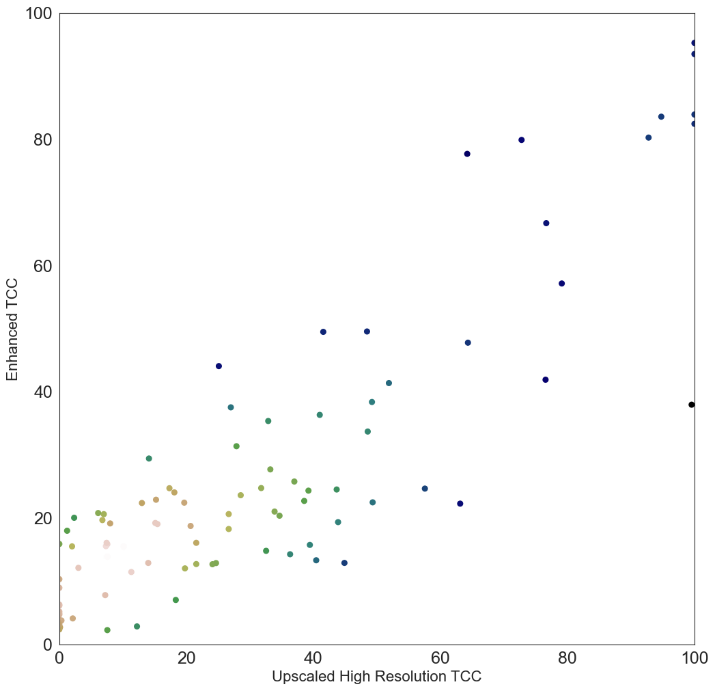

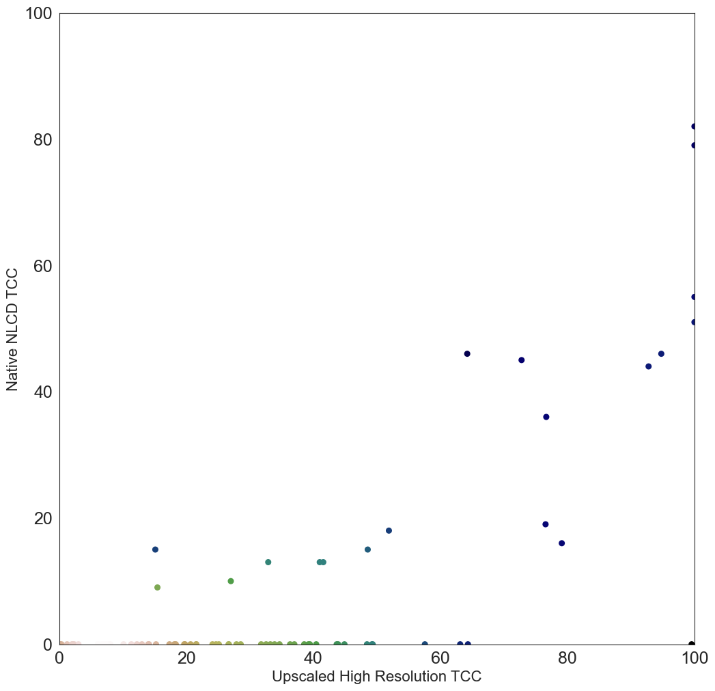


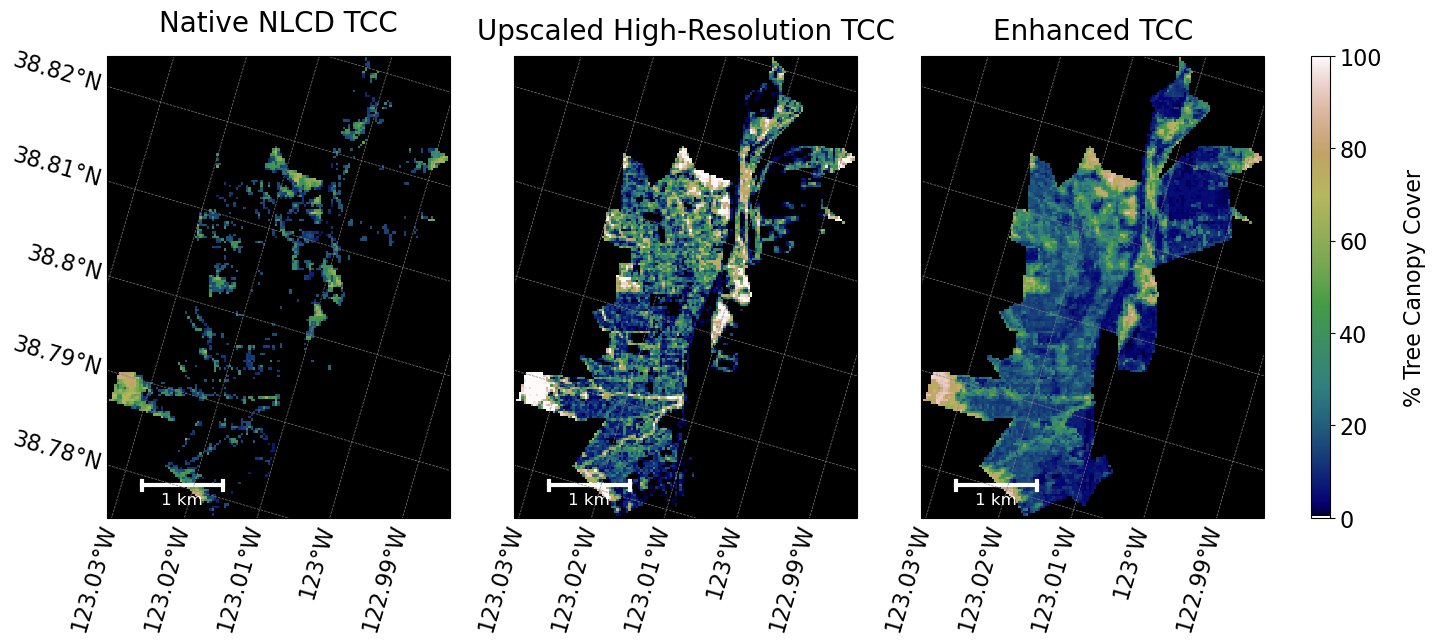


Denver, Colorado (city)

R-Squared 0.5029

RMSE: 11.6805

MAE: 8.473


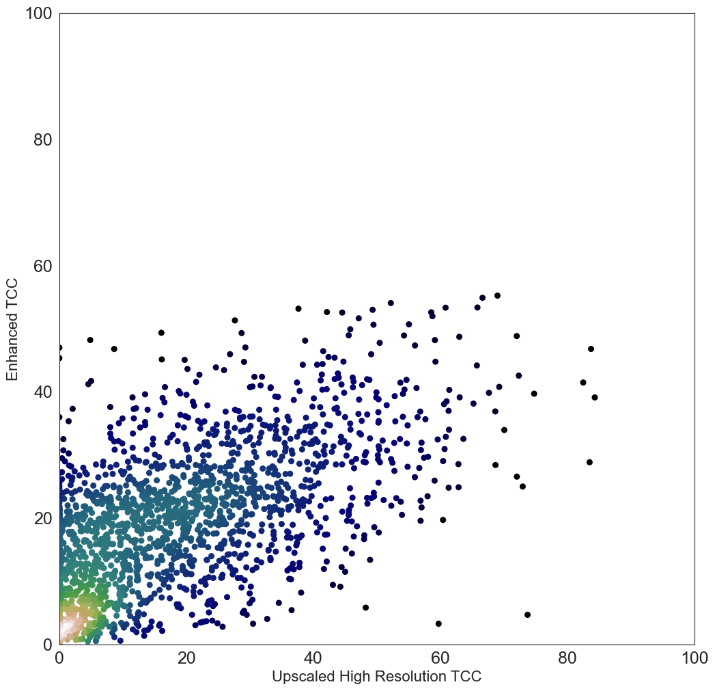

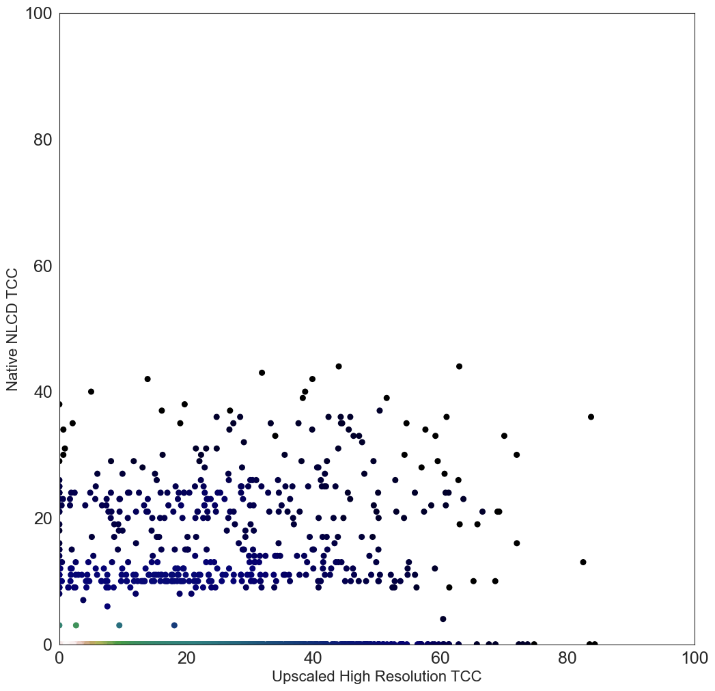


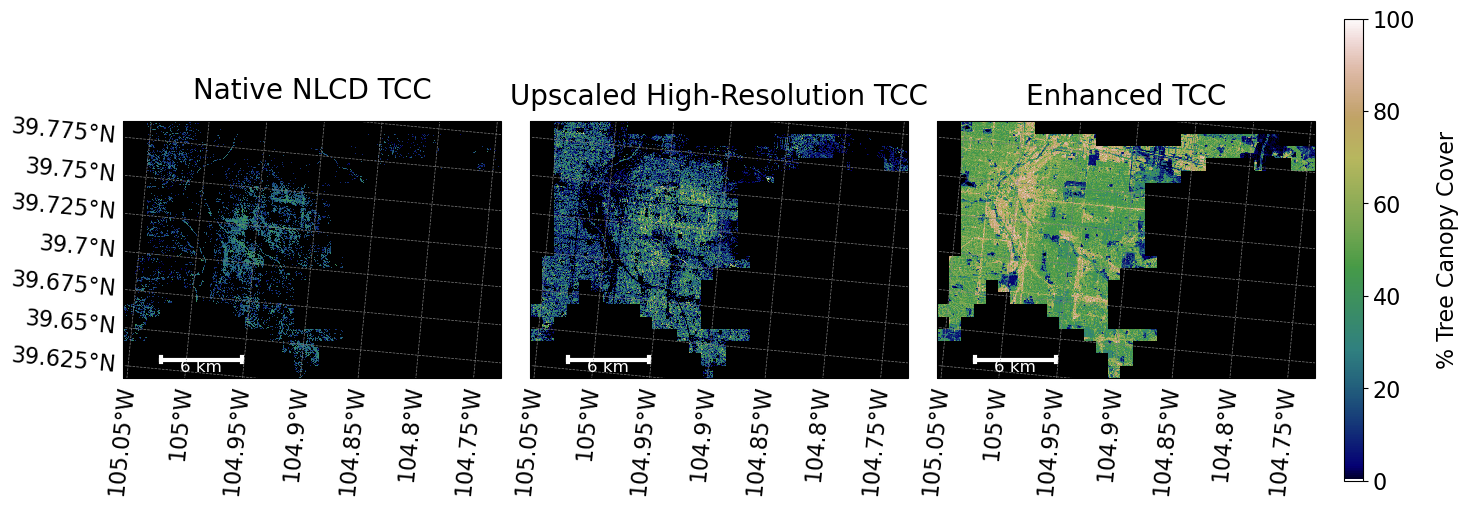


Des Moines, Iowa (city)

R-Squared 0.7066

RMSE: 15.7822

MAE: 11.221


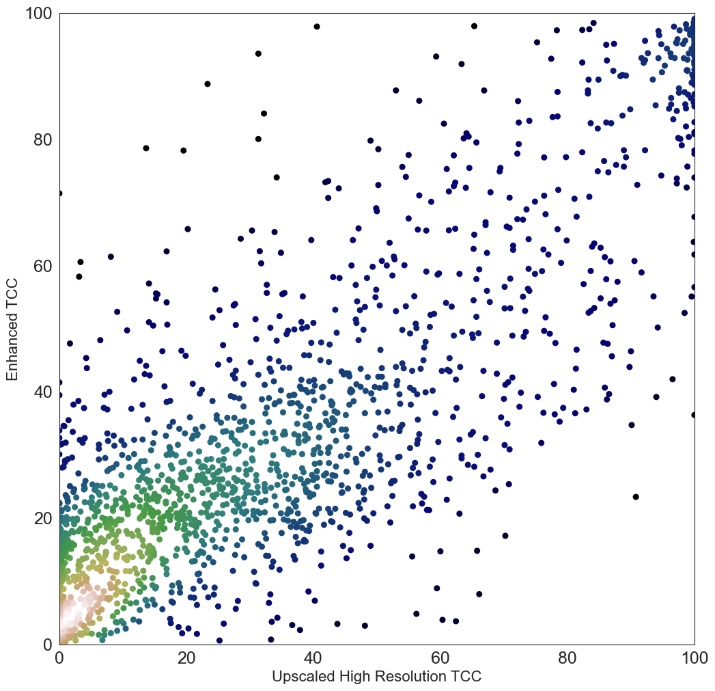

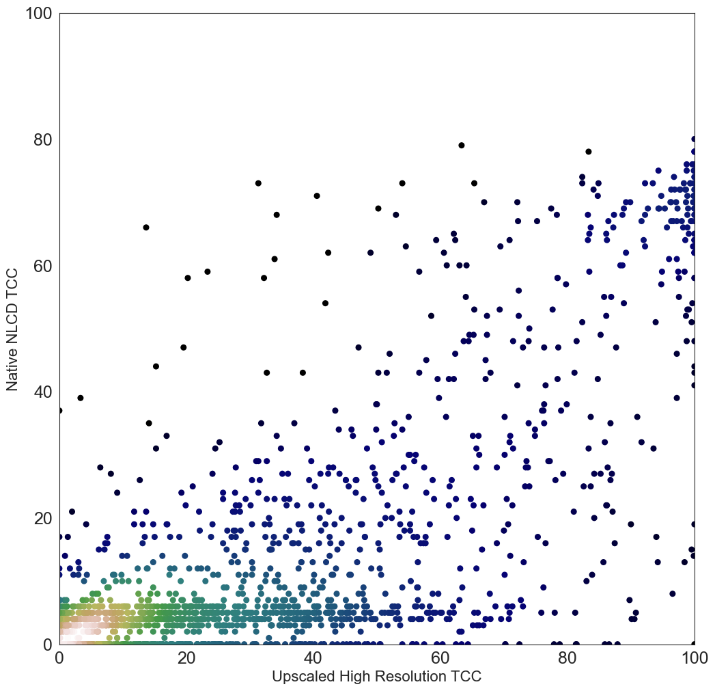


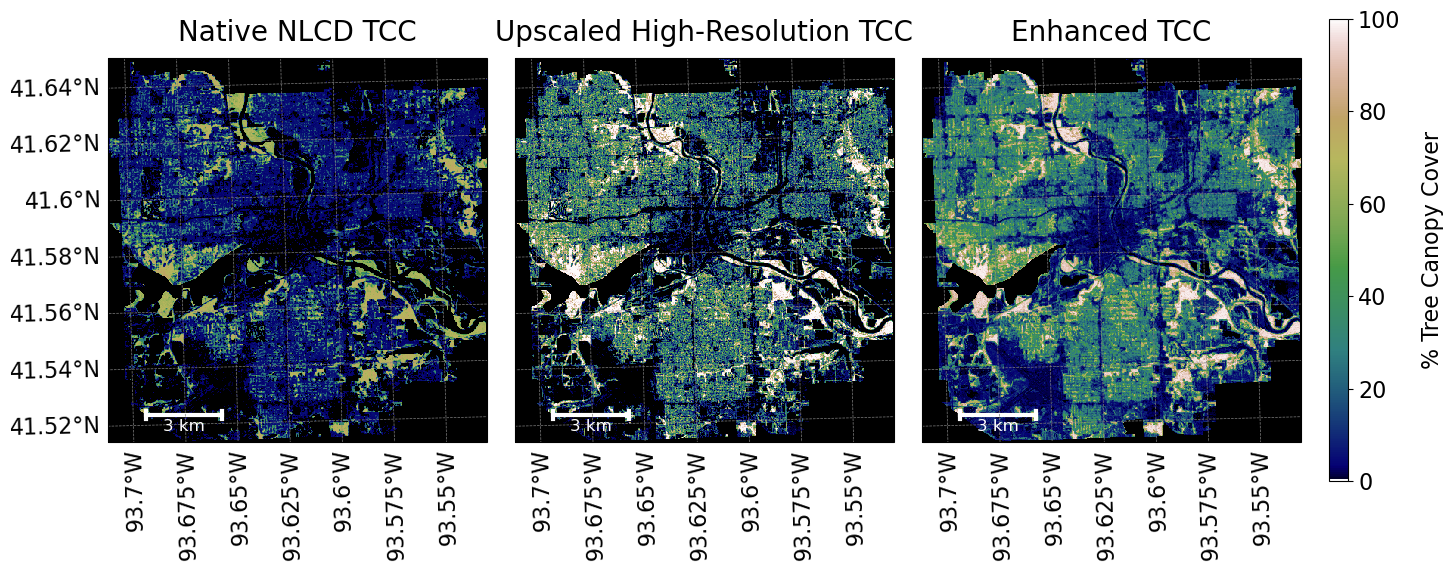


Des Moines, Iowa (urban area)

R-Squared 0.7198

RMSE: 16.024

MAE: 10.8045


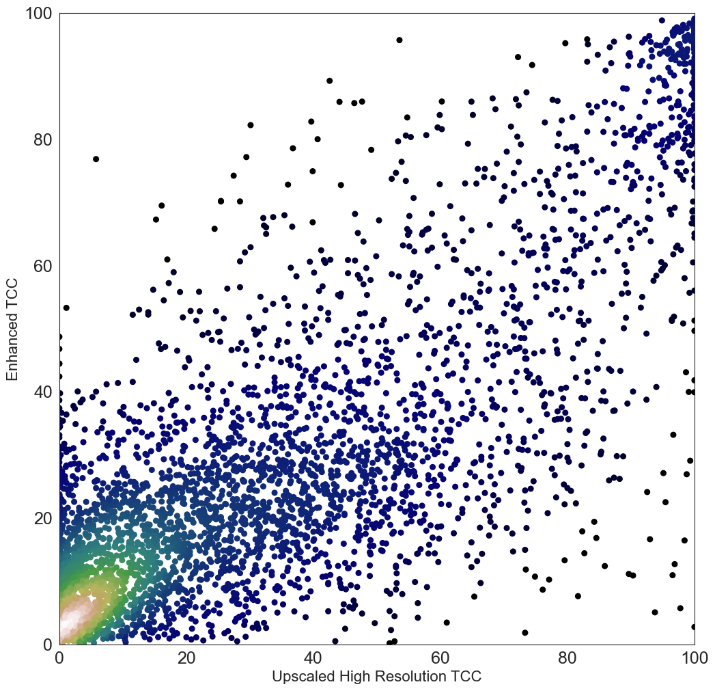

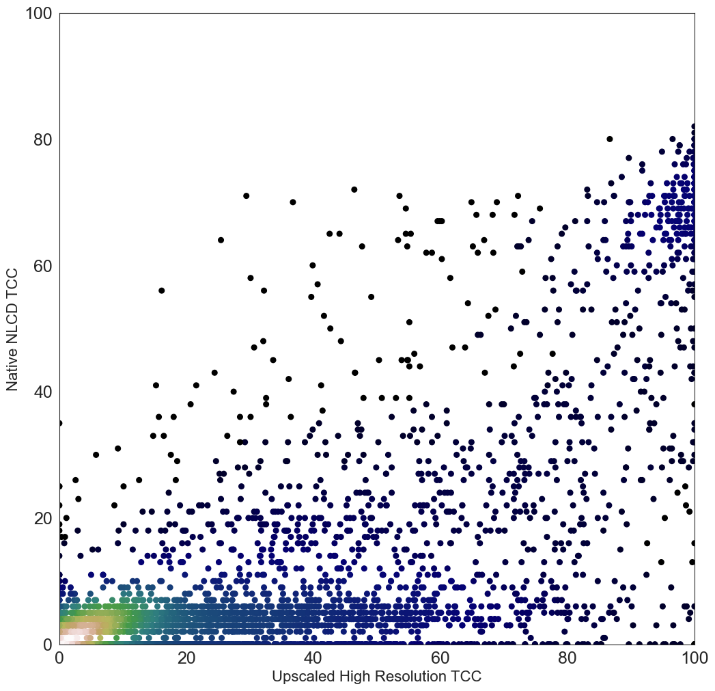


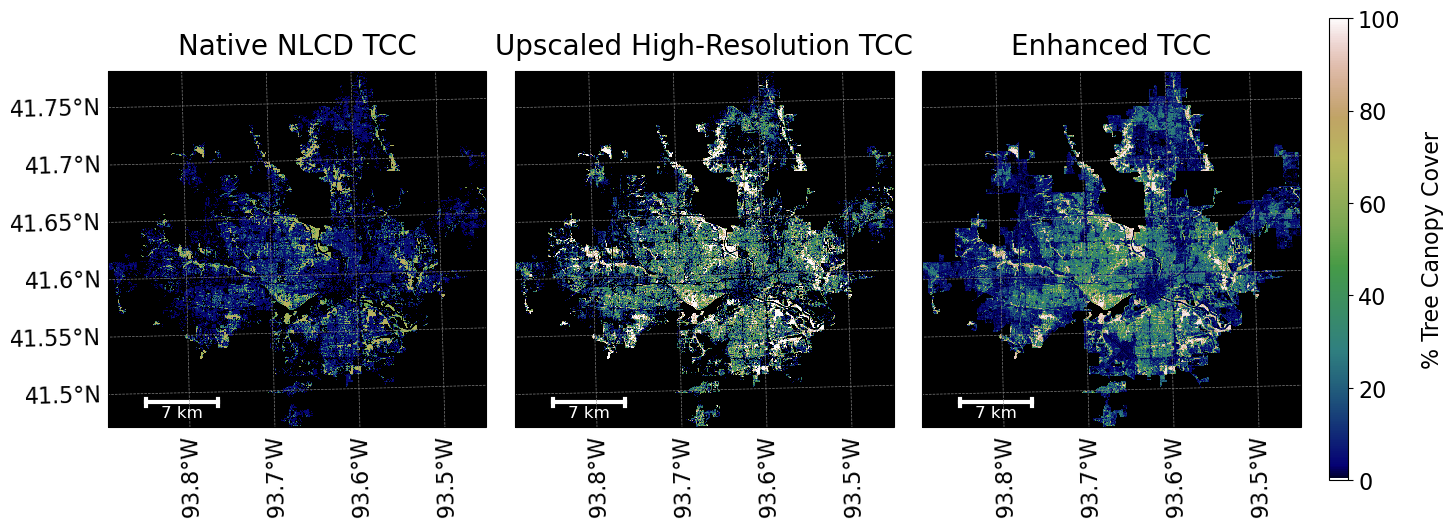


Durham, North Carolina (city)

R-Squared 0.7494

RMSE: 18.4785

MAE: 13.0178


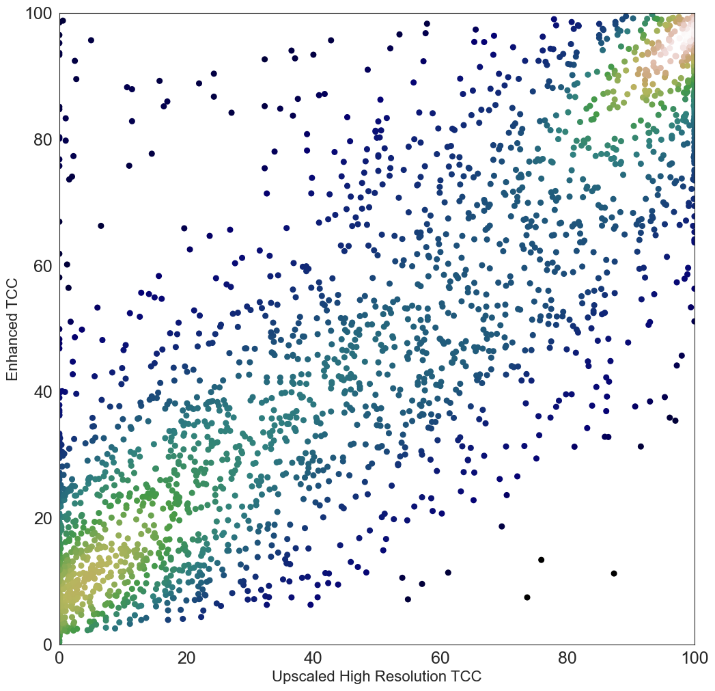

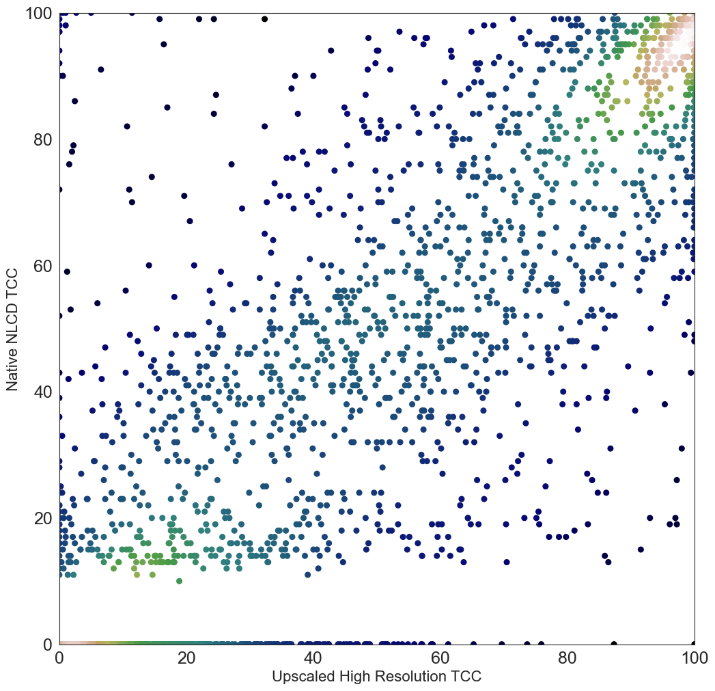


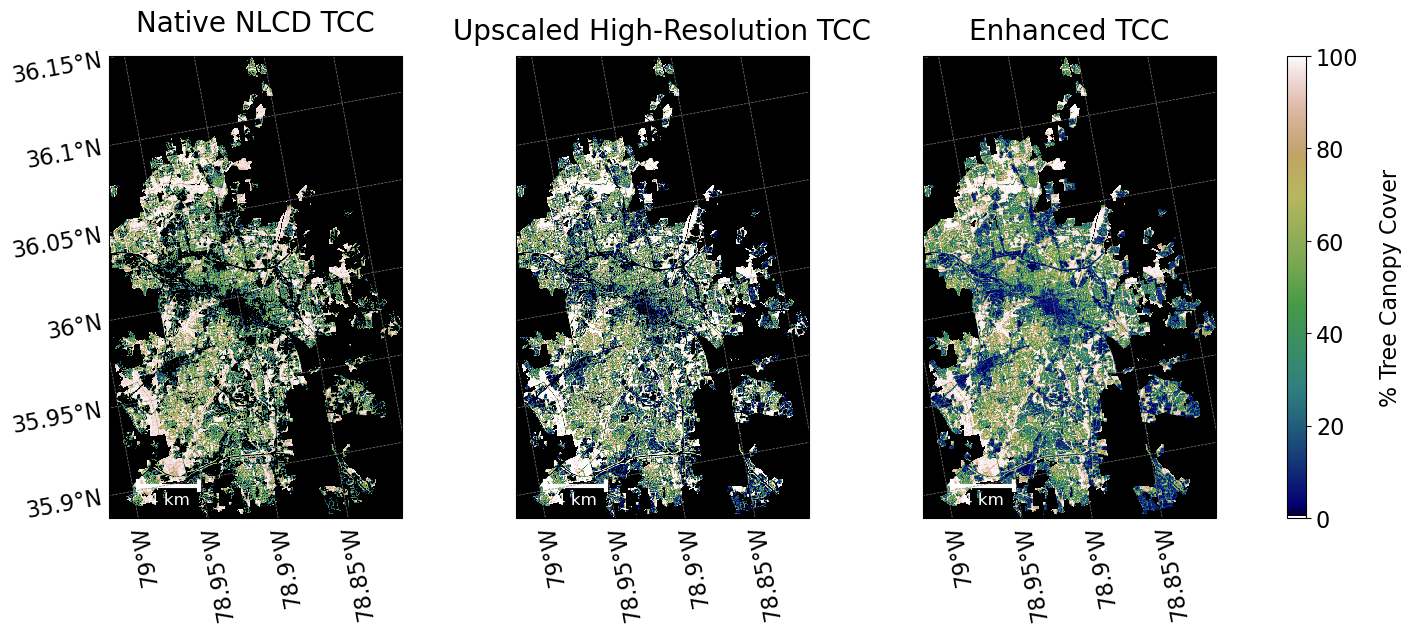


Durham, North Carolina (urban area)

R-Squared 0.7613

RMSE: 18.9464

MAE: 13.6925


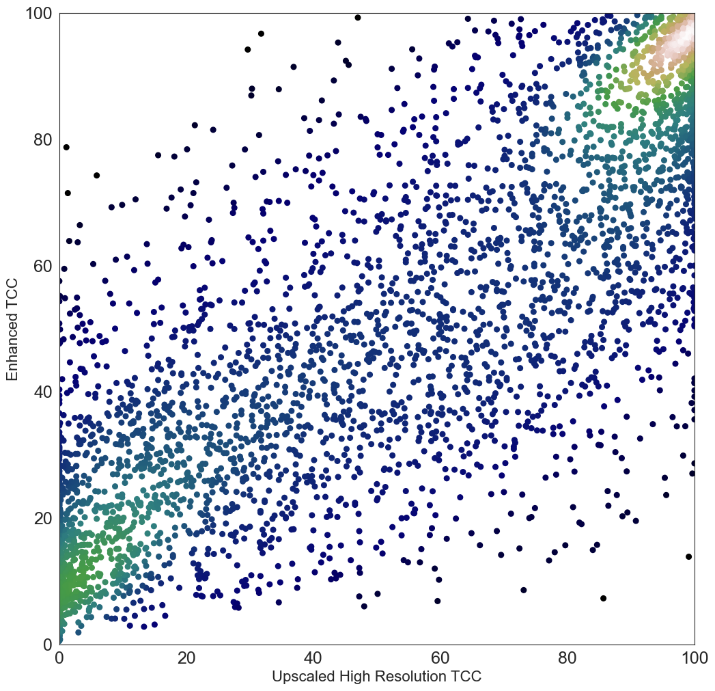

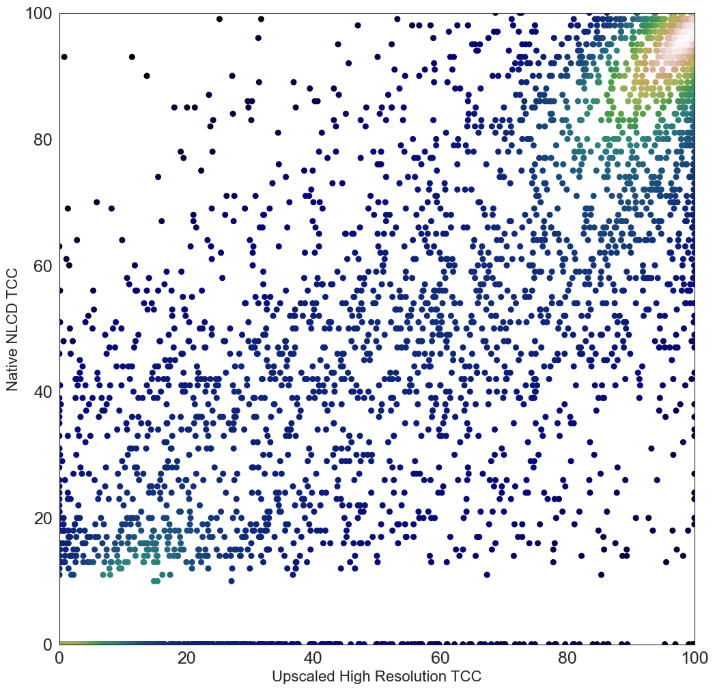


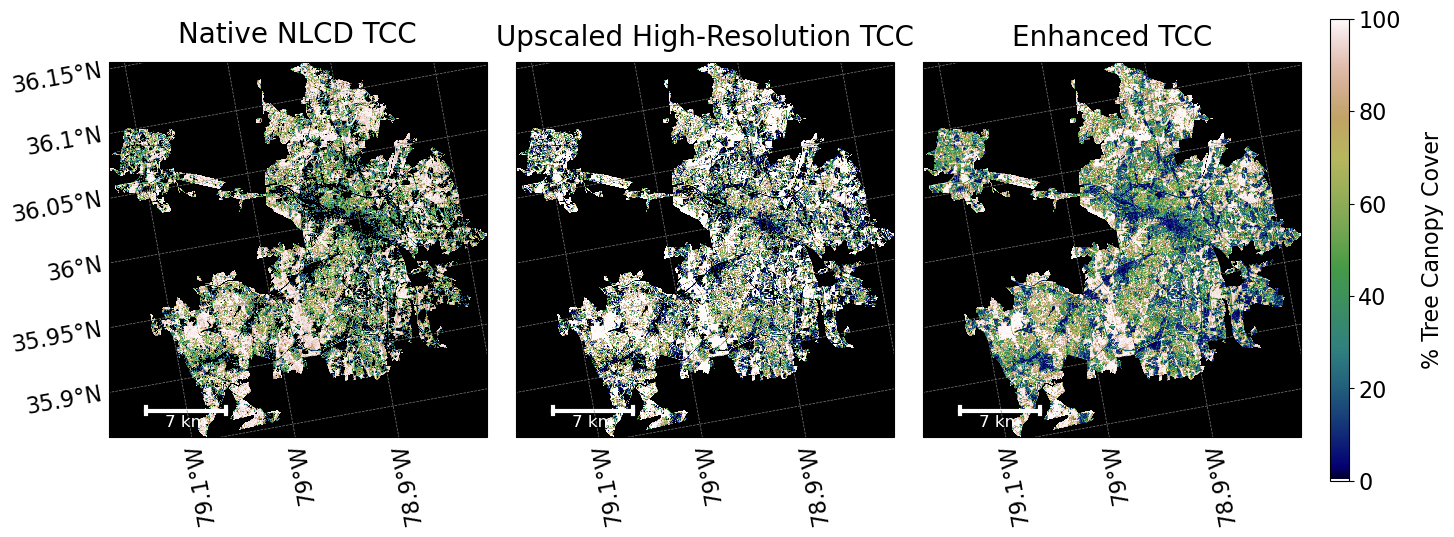


Forestville, California (urban area)

R-Squared 0.7364

RMSE: 18.5575

MAE: 14.2705


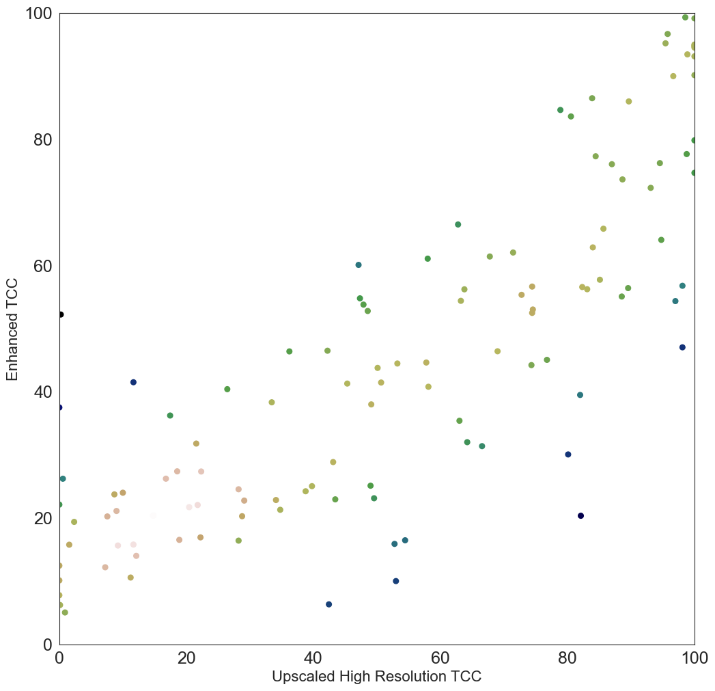

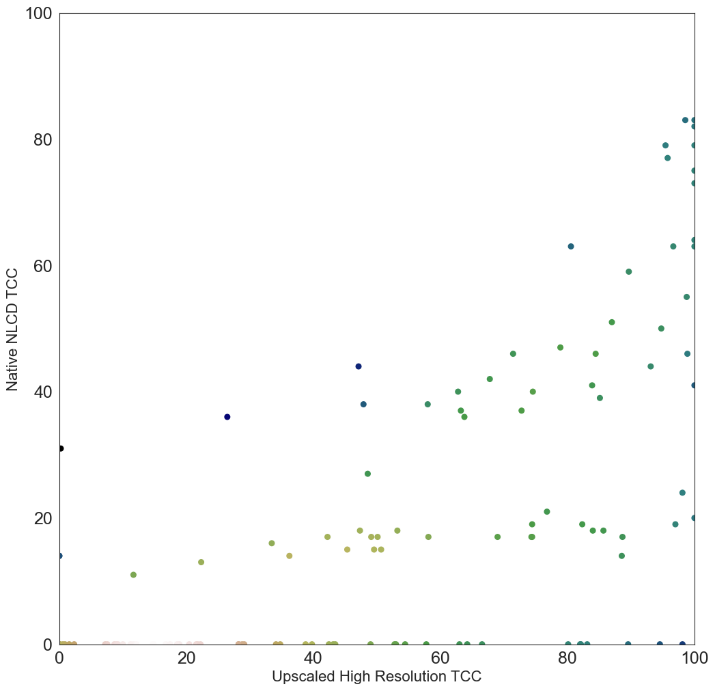


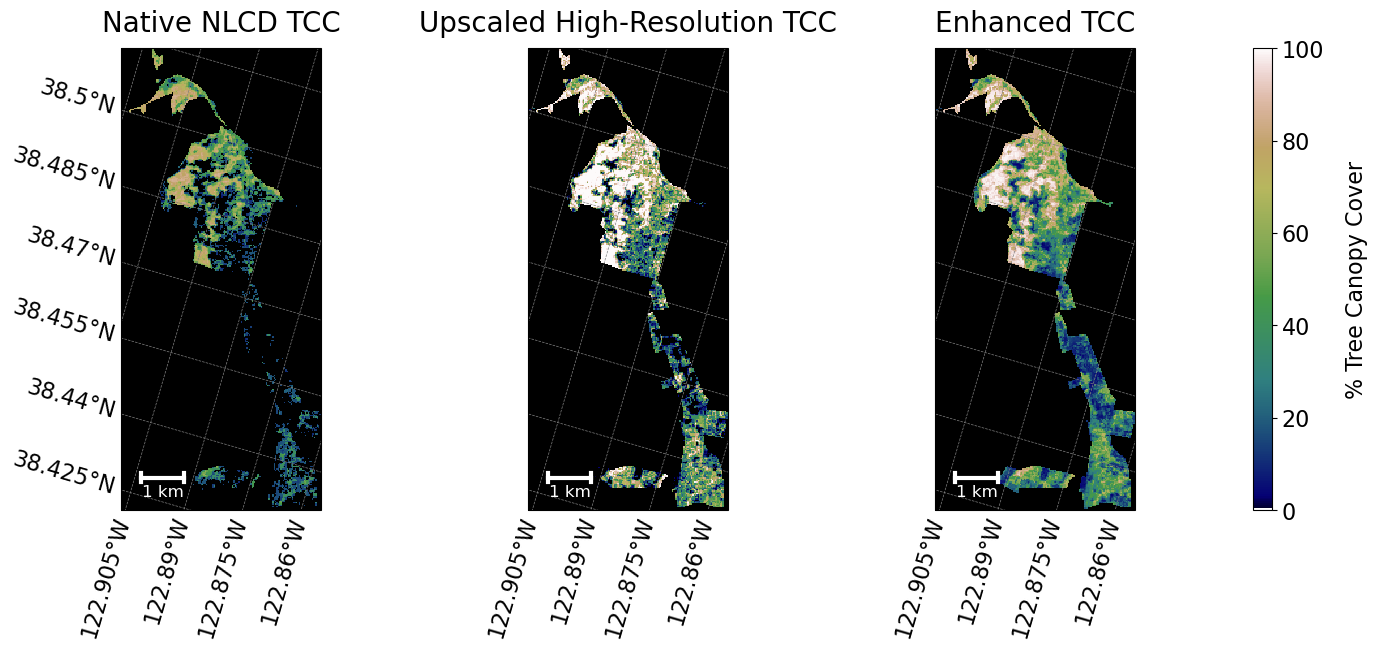


Fresno, California (urban area)

R-Squared 0.4655

RMSE: 12.7365

MAE: 8.3489


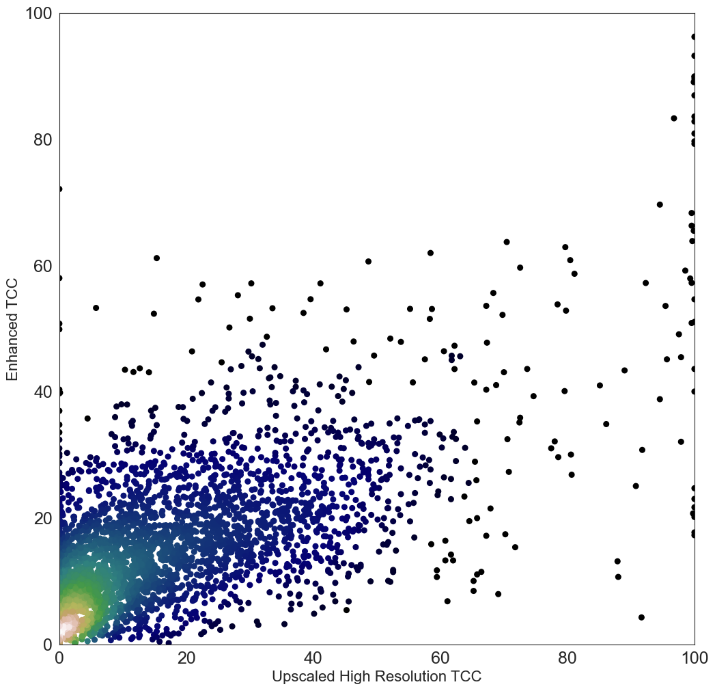

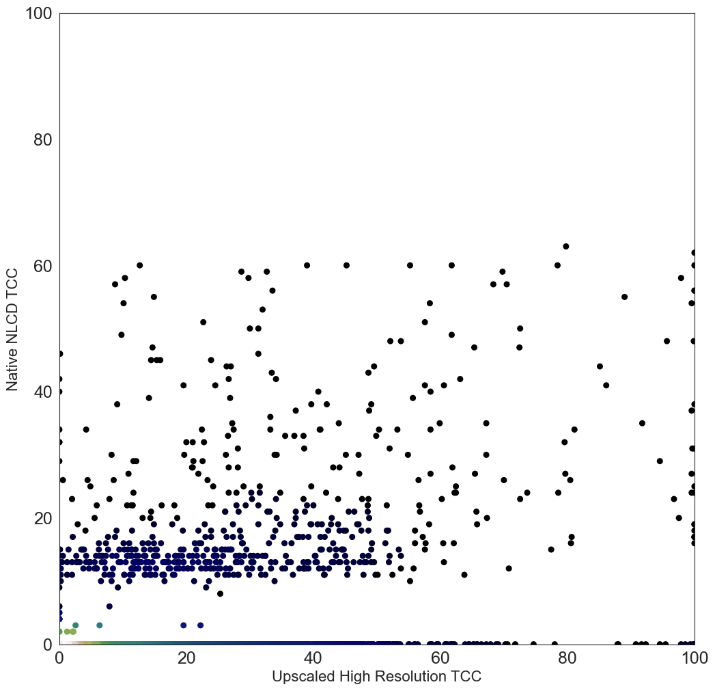


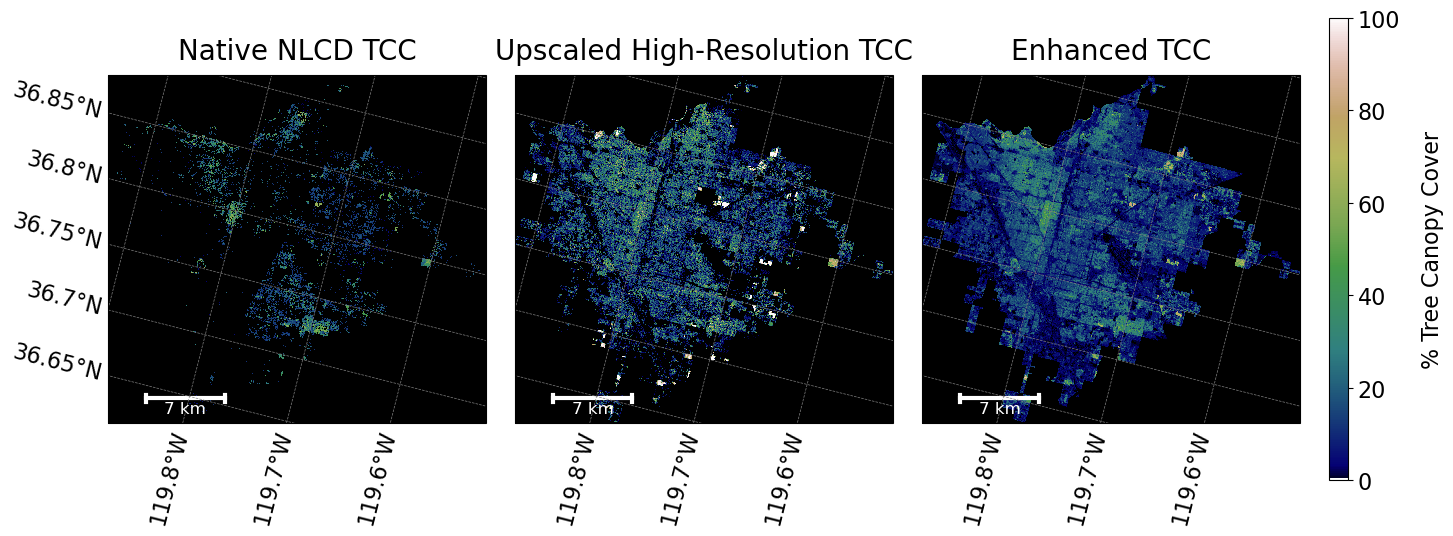


Green Bay, Wisconsin (urban area)

R-Squared 0.7372

RMSE: 17.0414

MAE: 12.0317


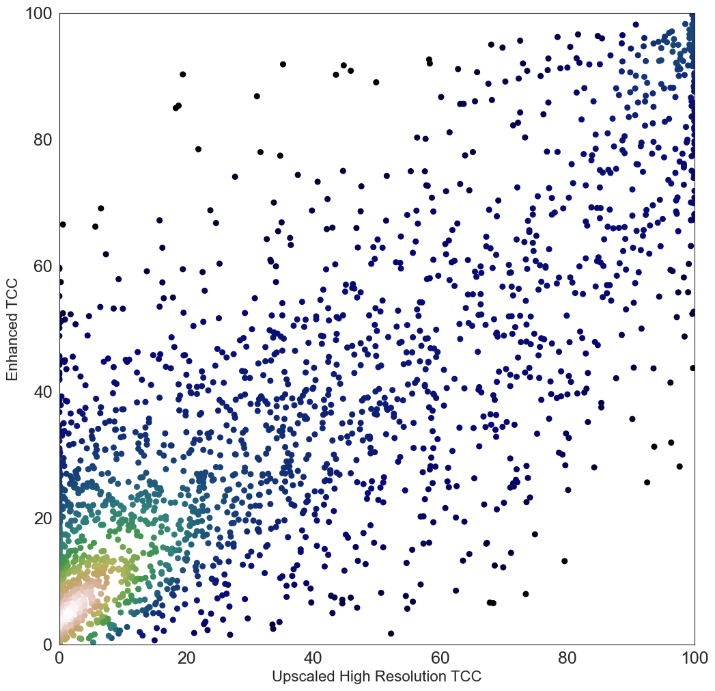

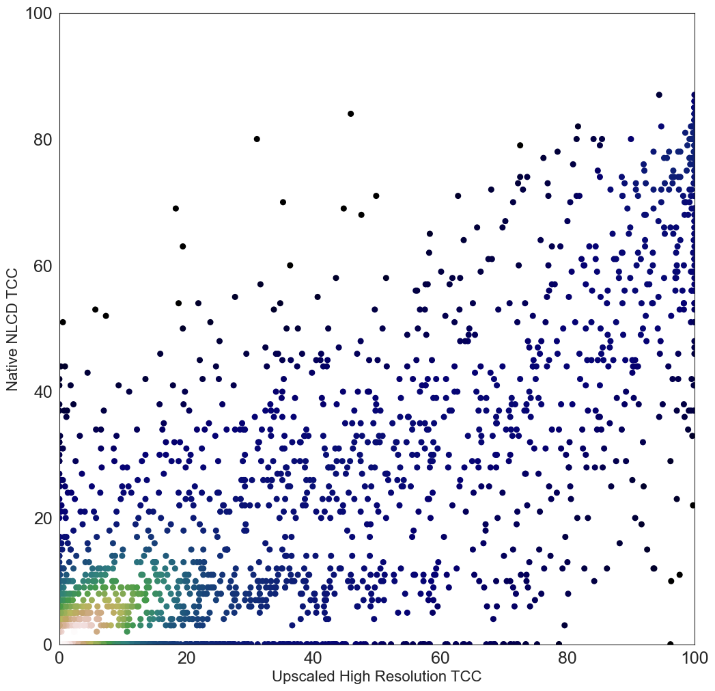


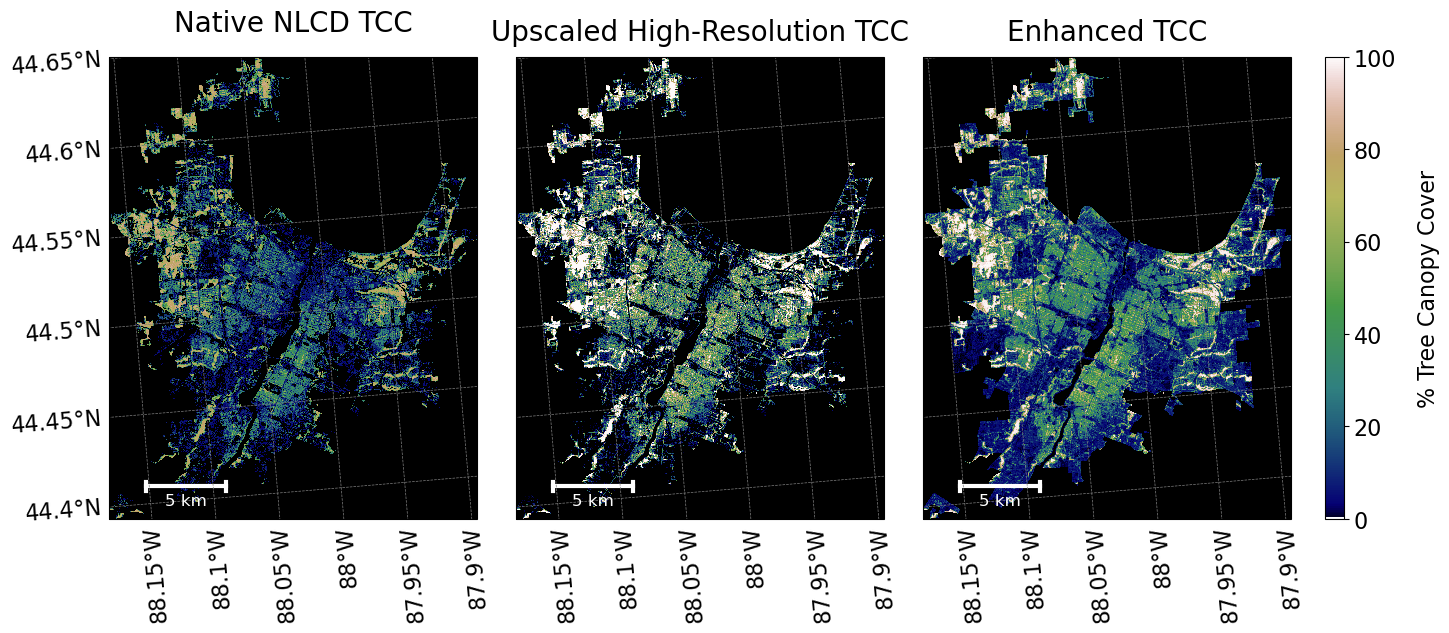


Guerneville, California (urban area)

R-Squared 0.8092

RMSE: 16.1164

MAE: 11.6993


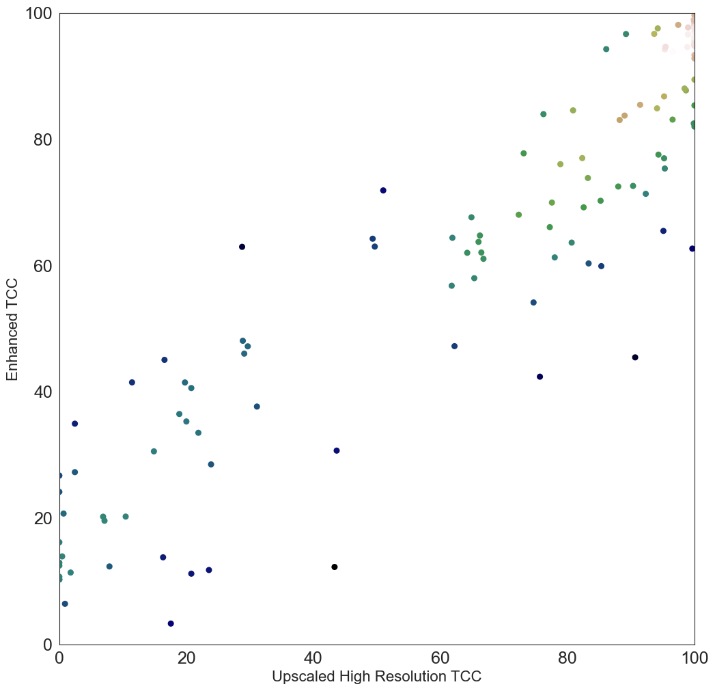

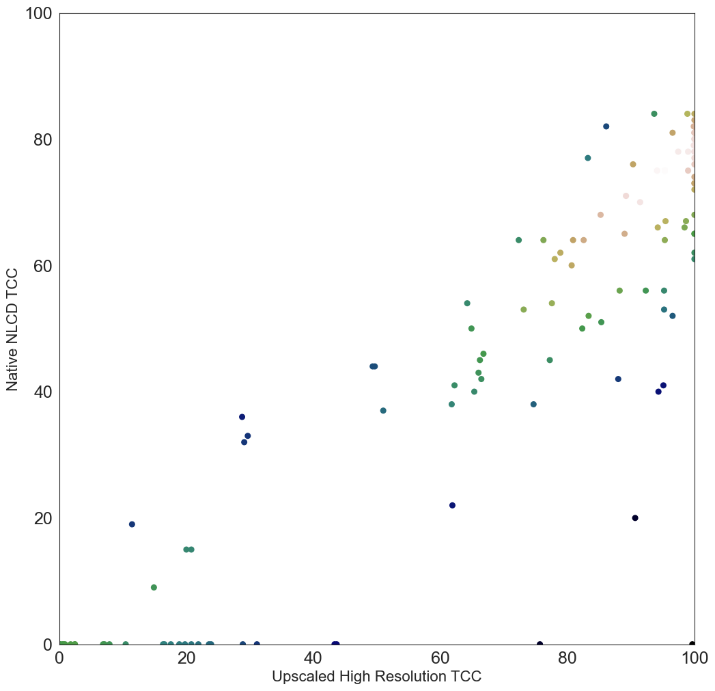


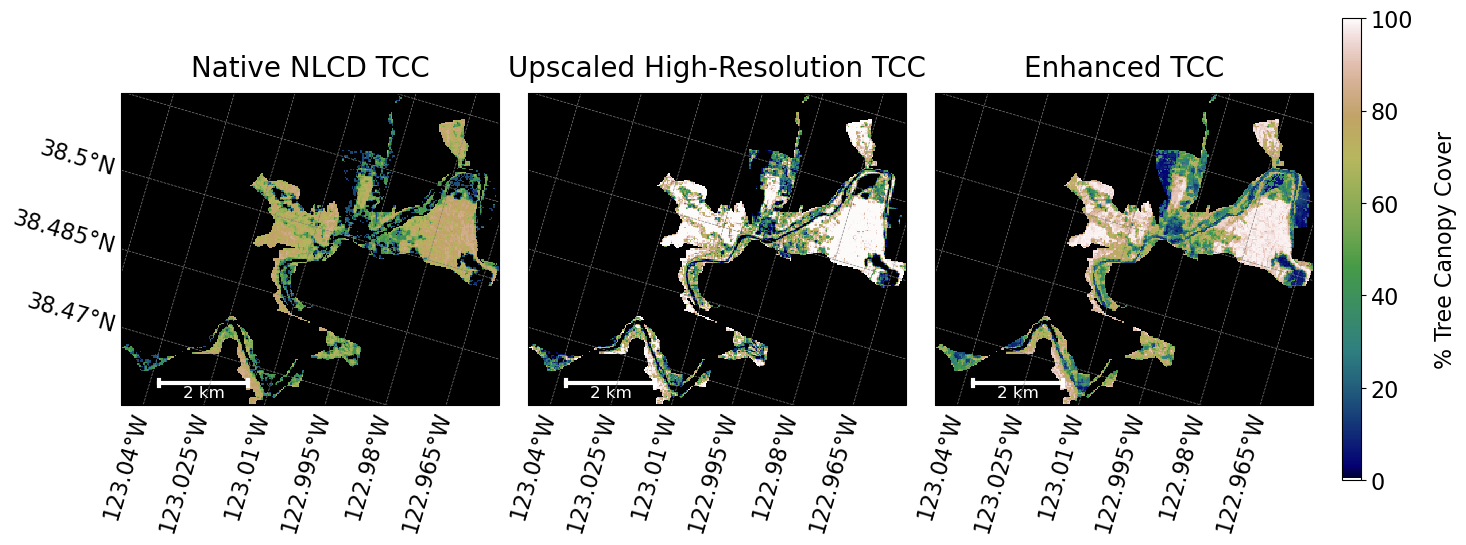


Harford County, Maryland (Aberdeen-Bel Air urban area)

R-Squared 0.7709

RMSE: 18.9001

MAE: 13.5118


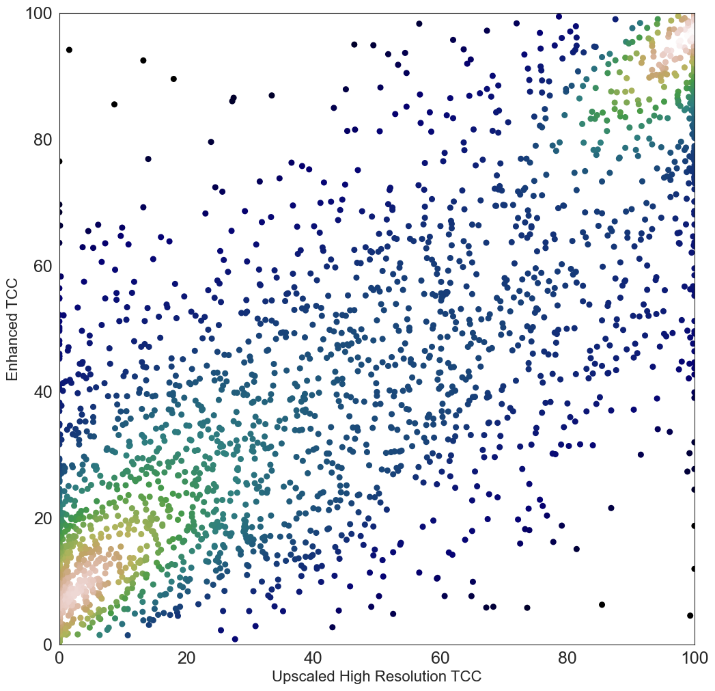

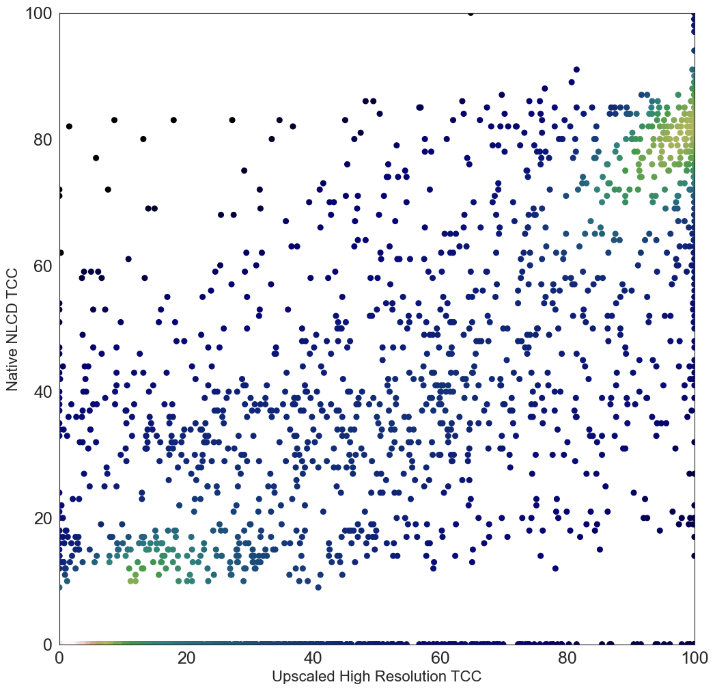


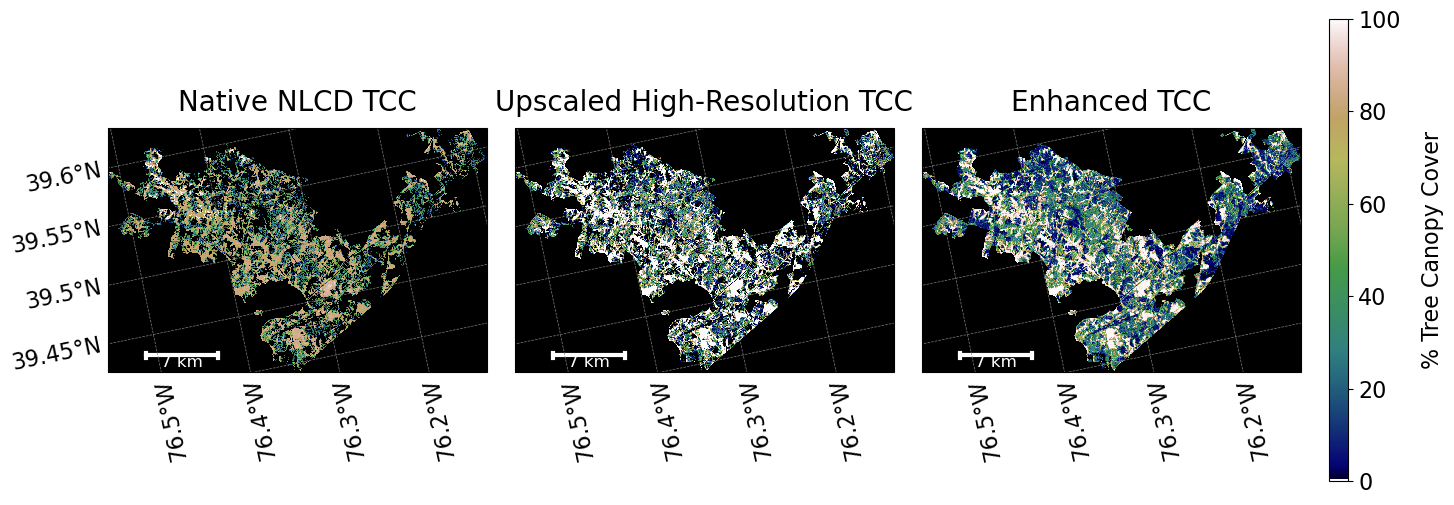


Howard County, Maryland (Baltimore urban area)

R-Squared 0.7104

RMSE: 21.0227

MAE: 15.196


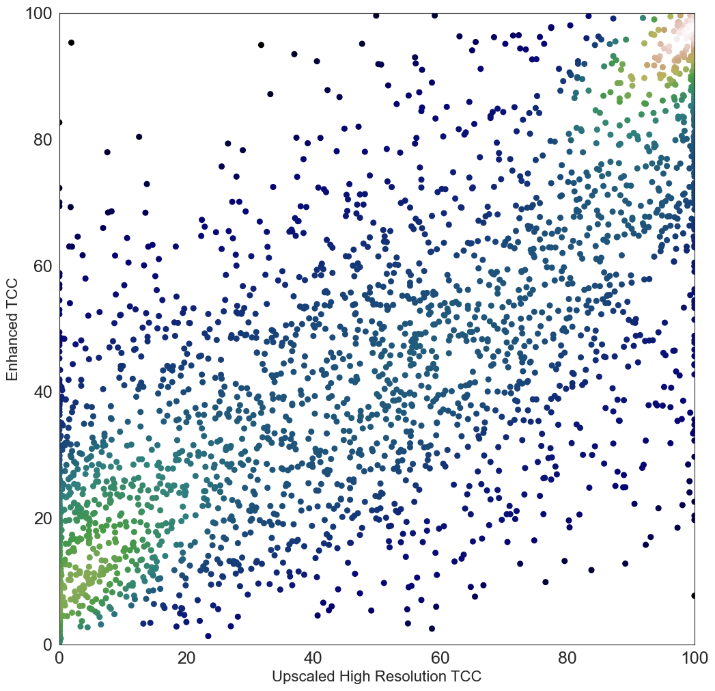

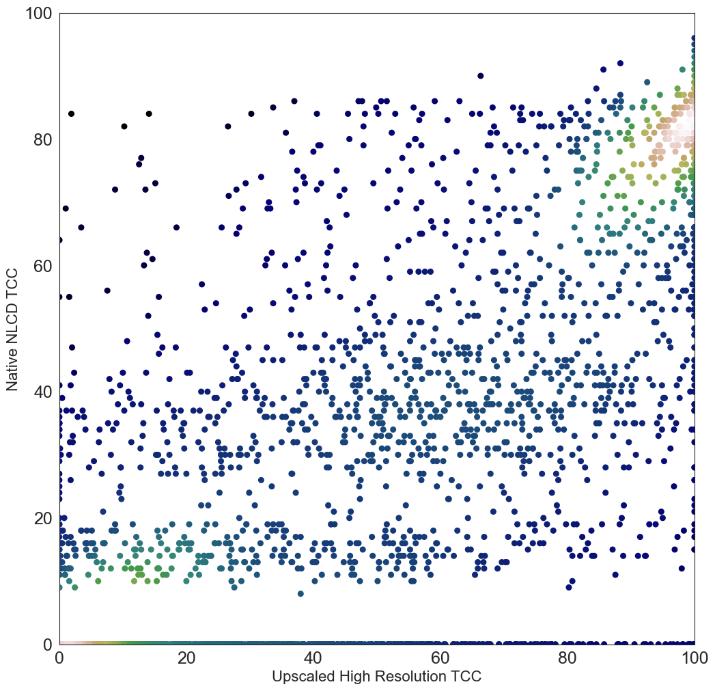


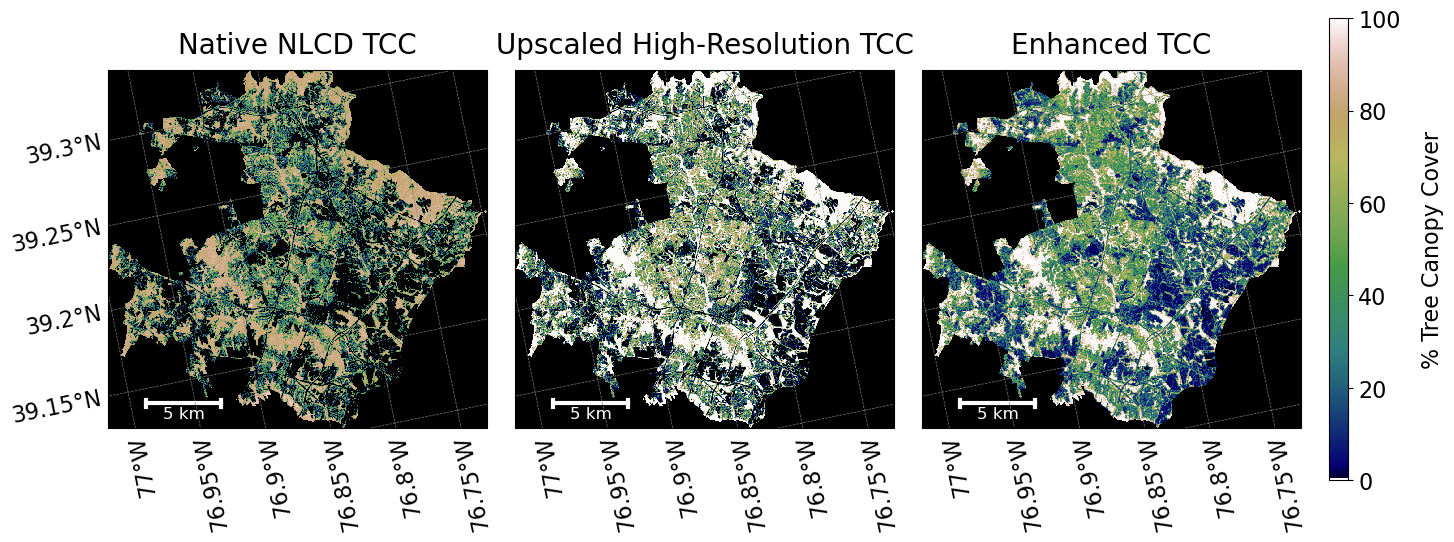


Kenton County, Kentucky (urban area)

R-Squared 0.8048

RMSE: 16.899

MAE: 12.162


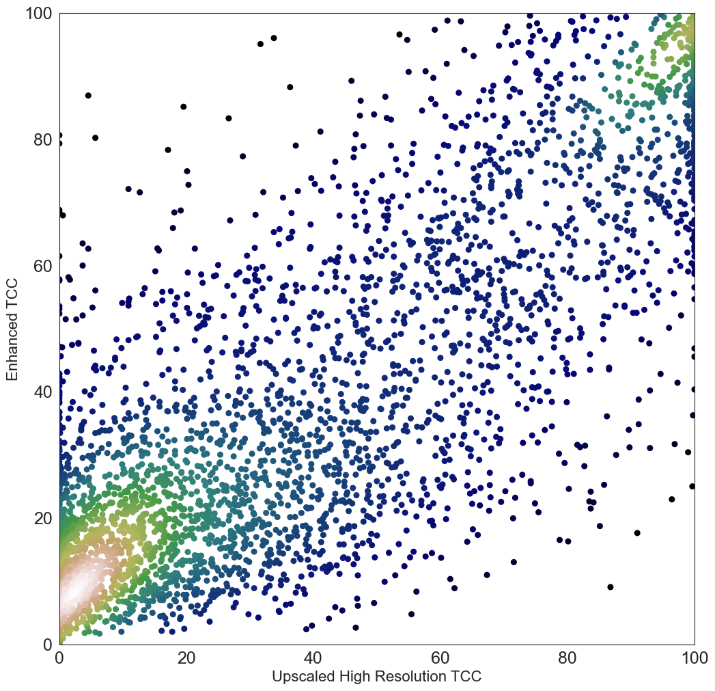

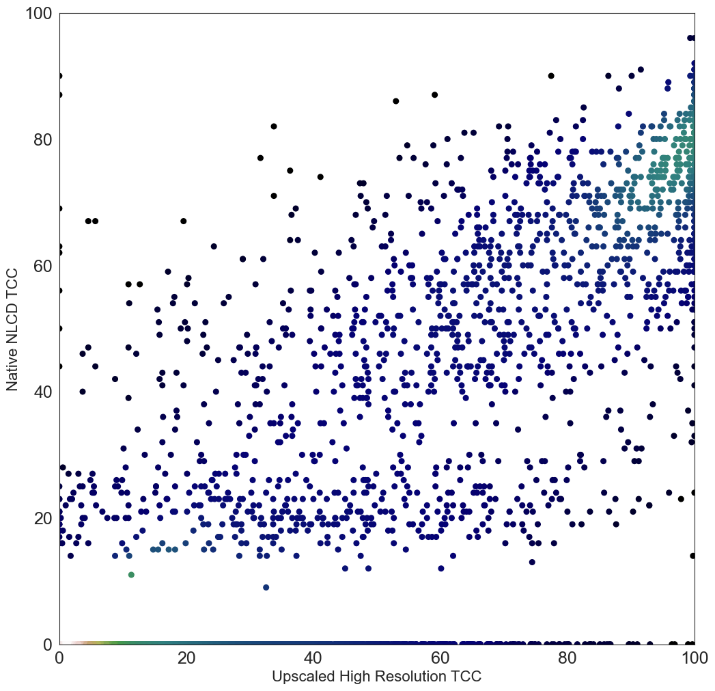


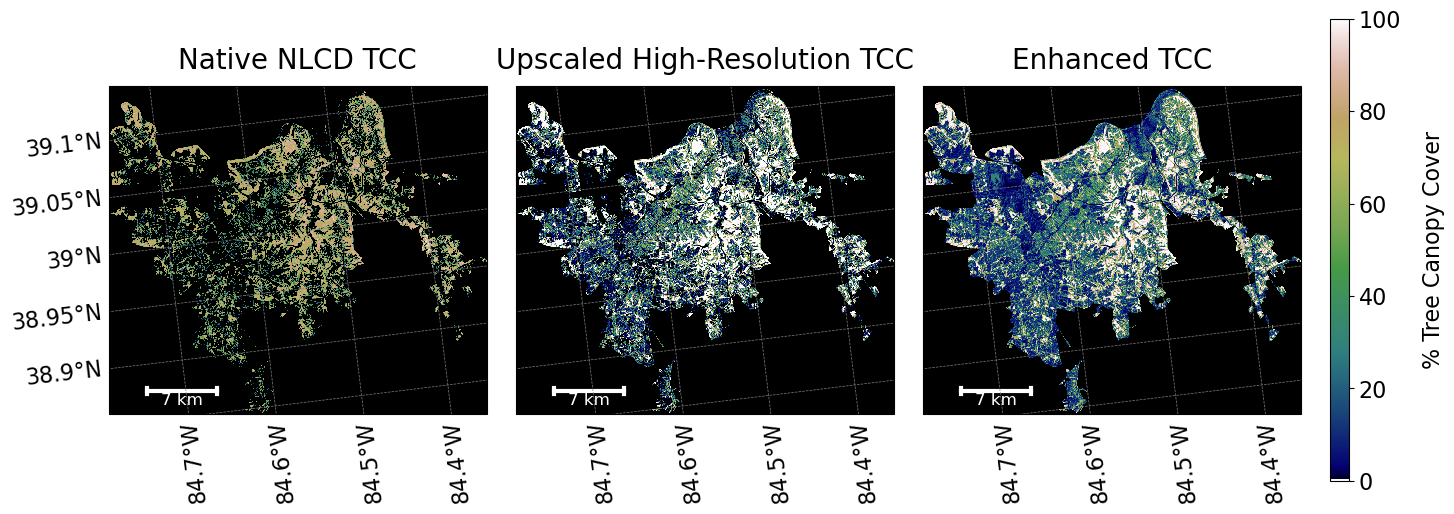


Lake Los Angeles, California (urban area)

R-Squared 0.3178

RMSE: 9.0787

MAE: 5.8907


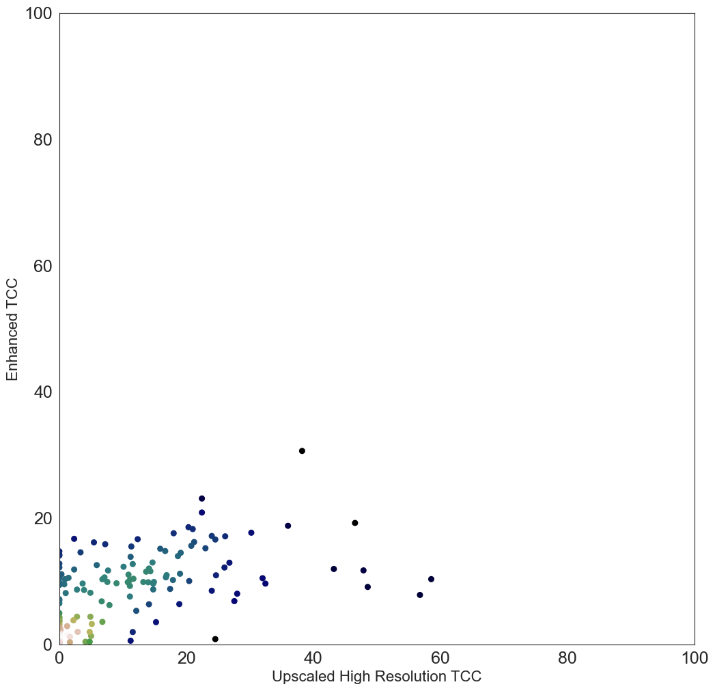

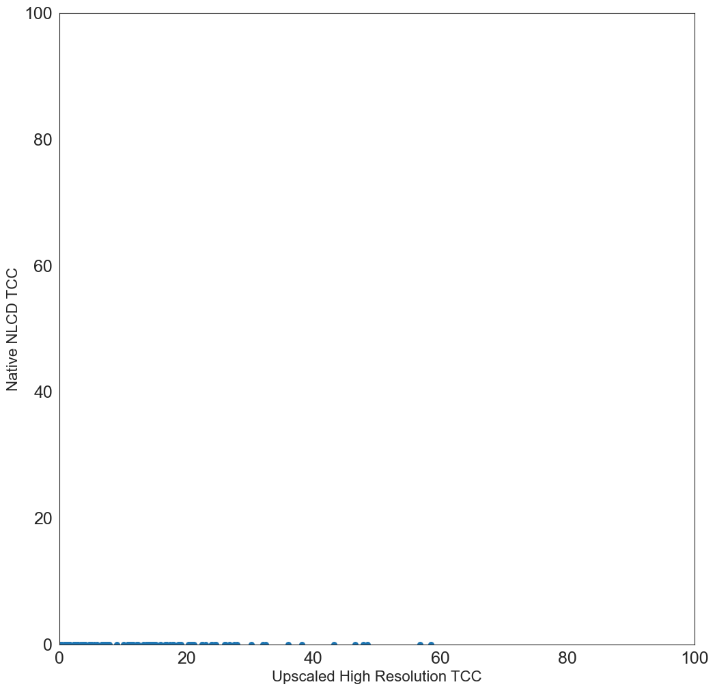


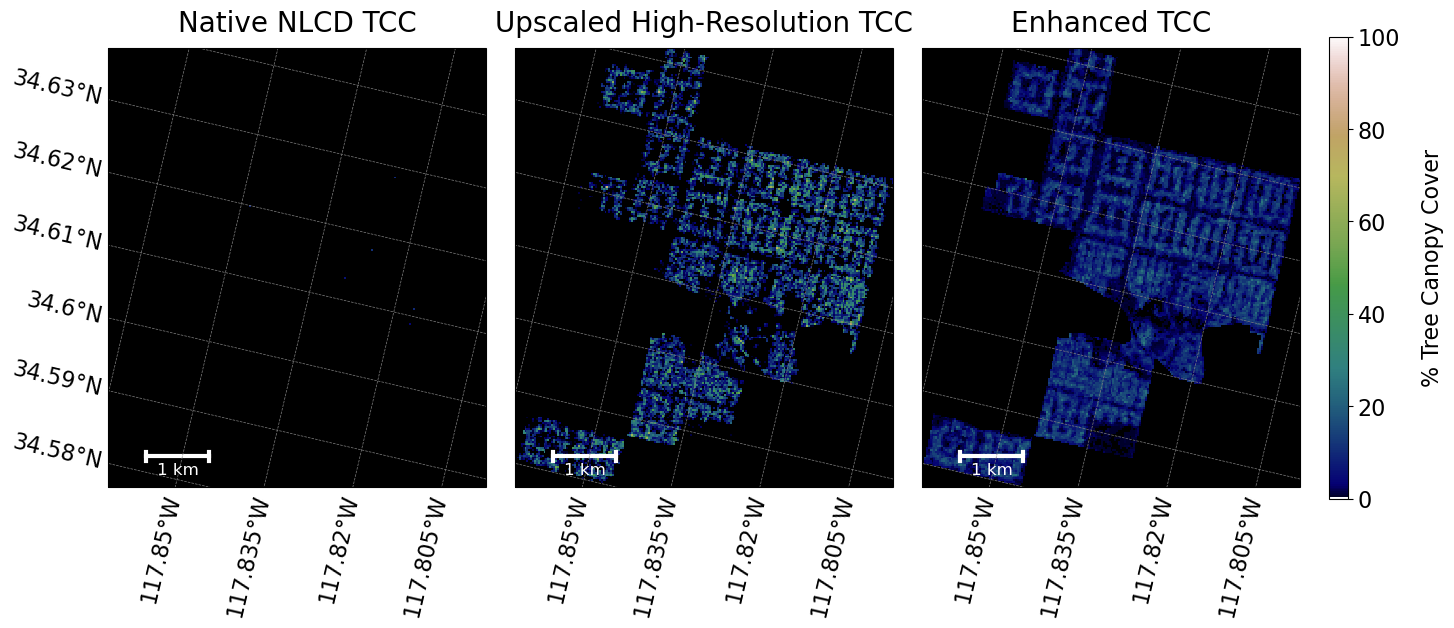


Lancaster--Palmdale, California (urban area)

R-Squared 0.4379

RMSE: 8.3412

MAE: 5.1571


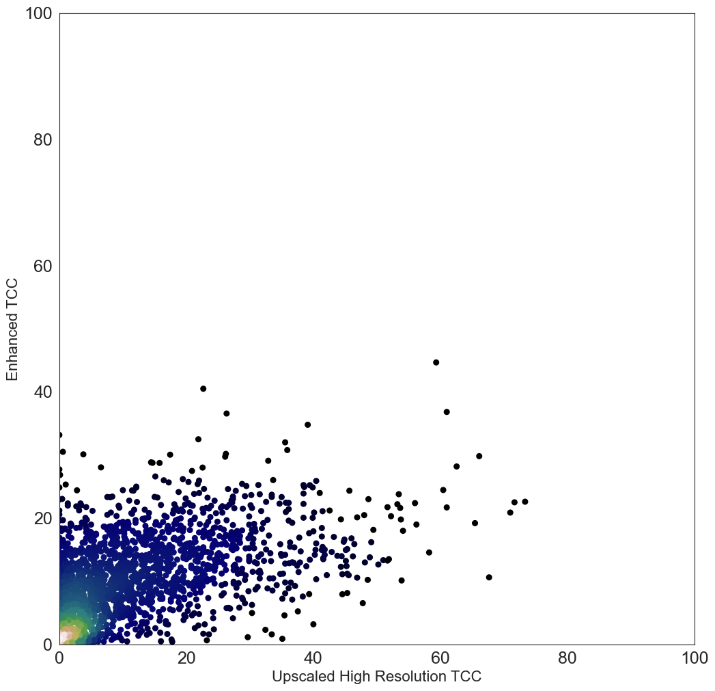

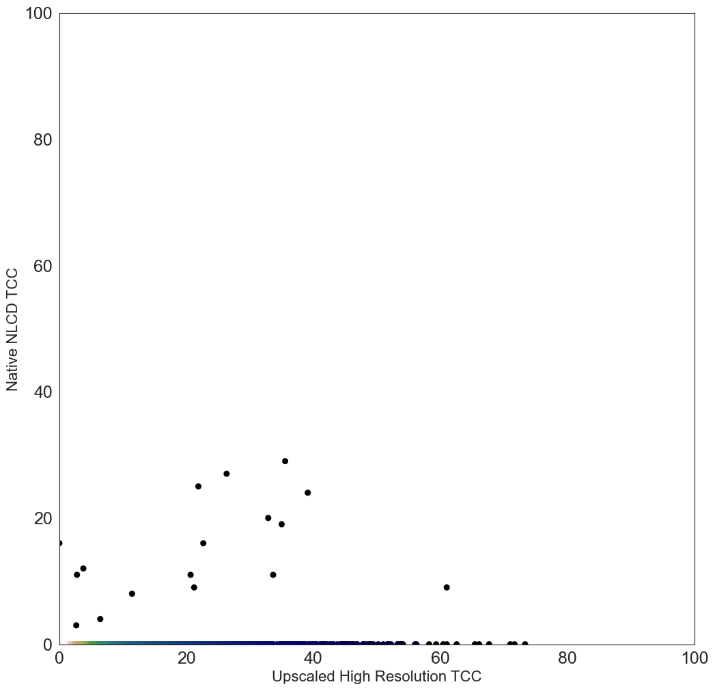


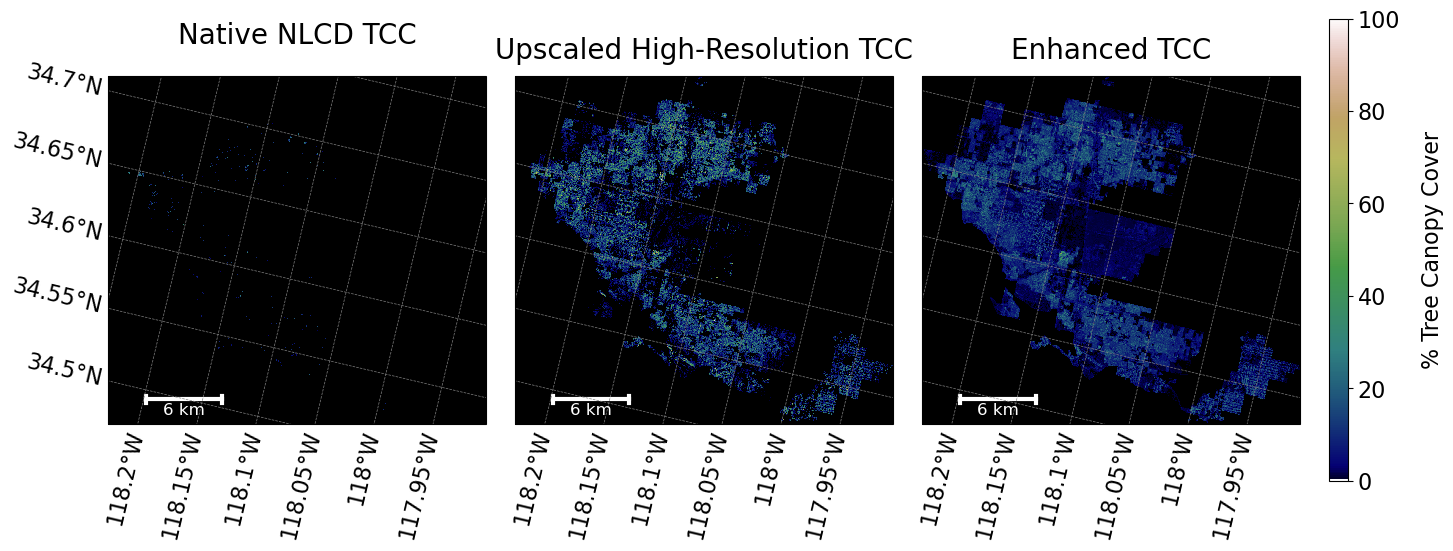


Los Angeles, California (urban area)

R-Squared 0.5138

RMSE: 14.9284

MAE: 10.8867


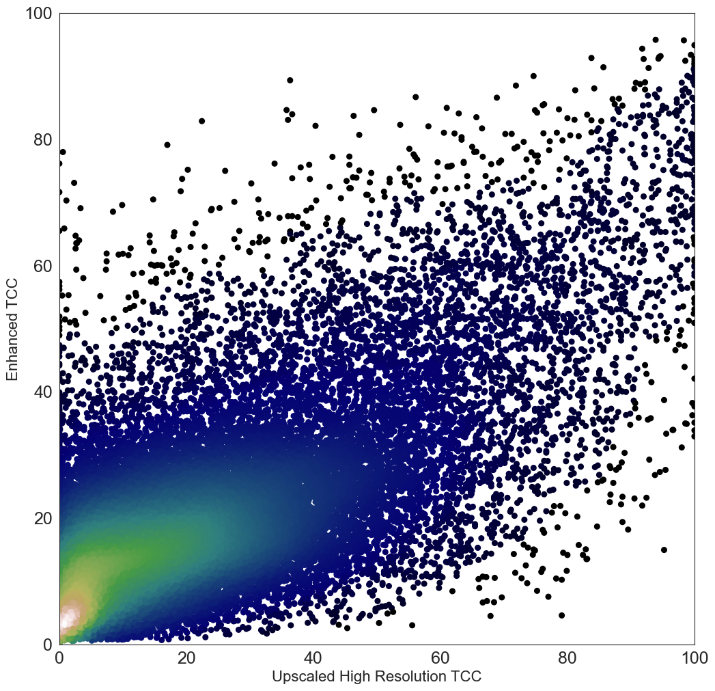


Memphis, Tennessee-Mississippi-Arkansas (urban area)

R-Squared 0.7389

RMSE: 18.8491

MAE: 13.3111

Milwaukee, Wisconsin (urban area)

R-Squared 0.6239

RMSE: 21.9501

MAE: 16.0765

Minneapolis-St. Paul, Minnesota-Wisconsin (urban area)

R-Squared 0.6445

RMSE: 17.8313

MAE: 12.6066

Montgomery County, Maryland (Washington, D.C., urban area)

R-Squared 0.6899

RMSE: 19.9528

MAE: 14.7625

Nampa, Idaho (city)

R-Squared 0.3304

RMSE: 10.3971

MAE: 7.4499

New Bedford, Massachusetts (urban area)

R-Squared 0.79

RMSE: 18.5292

MAE: 12.7289

New Haven, Connecticut (urban area)

R-Squared 0.7757

RMSE: 17.8399

MAE: 12.6352

New York, New York (city)

R-Squared 0.6432

RMSE: 14.8443

MAE: 10.2757

New York, New York (city)

R-Squared 0.6294

RMSE: 15.3515

MAE: 10.9903

Patterson, New Jersey (city)

R-Squared 0.6647

RMSE: 17.2248

MAE: 12.5837

Petaluma, California (urban area)

R-Squared 0.416

RMSE: 15.4141

MAE: 11.3874

Philadelphia, Pennsylvania (city

R-Squared 0.7308

RMSE: 14.5548

MAE: 9.7001

Philadelphia, Pennsylvania-New Jersey-Delaware-Maryland (urban area)

R-Squared 0.7203

RMSE: 19.8823

MAE: 14.2952

Phoenix, Arizona (urban area)

R-Squared 0.2932

RMSE: 9.2535

MAE: 6.2484

Pittsburgh, Pennsylvania (city)

R-Squared 0.7663

RMSE: 17.8438

MAE: 12.7854

Pittsburgh, Pennsylvania (urban area)

R-Squared 0.7808

RMSE: 18.5934

MAE: 13.0223

Poolesville, Maryland (Montgomery County urban area)

R-Squared 0.616

RMSE: 22.0883

MAE: 15.7465

Portland, Maine (urban area)

R-Squared 0.8142

RMSE: 17.316

MAE: 11.9322

Portland, Oregon (urban area)

R-Squared 0.7528

RMSE: 15.3257

MAE: 10.7192

Salt Lake City, Utah (urban area)

R-Squared 0.5015

RMSE: 13.0226

MAE: 8.656

San Diego, California (city)

R-Squared 0.4596

RMSE: 13.7914

MAE: 9.5339

San Jose, California (city)

R-Squared 0.4439

RMSE: 13.314

MAE: 9.5009

Santa Clarita, California (urban area)

R-Squared 0.3723

RMSE: 14.0906

MAE: 9.9089

Santa Rosa, California (urban area)

R-Squared 0.6296

RMSE: 16.6189

MAE: 12.2209

Seattle, Washington (city)

R-Squared 0.6391

RMSE: 15.9104

MAE: 11.6074

Shady Side --Deale, Maryland (urban area)

R-Squared 0.5716

RMSE: 24.6967

MAE: 17.6185

Shannondale, West Virginia (Jefferson County urban area)

R-Squared 0.4327

RMSE: 15.1471

MAE: 10.5068

Sonoma, California (urban area)

R-Squared 0.5998

RMSE: 18.4852

MAE: 13.7741

St. Louis, Missouri-Illinois (urban area)

R-Squared 0.7495

RMSE: 18.168

MAE: 12.804

Syracuse, New York (city)

R-Squared 0.708

RMSE: 15.3865

MAE: 11.6041

Tampa--St. Petersburg, Florida (urban area)

R-Squared 0.613

RMSE: 21.4337

MAE: 16.0429

Virginia Beach, Virginia (city)

R-Squared 0.723

RMSE: 18.9645

MAE: 14.0306

Virginia Beach, Virginia (urban area)

R-Squared 0.6642

RMSE: 21.0522

MAE: 15.3192

Washington, D.C. (city

R-Squared 0.7161

RMSE: 19.1376

MAE: 13.3245

Washington, D.C.-Virginia-Maryland (urban area)

R-Squared 0.6784

RMSE: 21.3321

MAE: 15.7144

Wicomico County, Maryland (Salisbury urban area)

R-Squared 0.672

RMSE: 22.0025

MAE: 15.6852

Williamsburg, Virginia (urban area)

R-Squared 0.6709

RMSE: 21.8732

MAE: 15.7274

# References

1. Housman, I. *et al.* *National Land Cover Database Tree Canopy Cover Methods*. 26 https://data.fs.usda.gov/geodata/rastergateway/treecanopycover/docs/TCC_v2021-4_Methods.pdf (2023).

### Disclaimer

Any use of trade, firm, or product names is for descriptive purposes only and does not imply endorsement by the U.S. Government.
